# Association of tumor microbiome with survival in resected early-stage PDAC

**DOI:** 10.1128/msystems.01229-24

**Published:** 2025-02-27

**Authors:** Yixuan Meng, Chan Wang, Mykhaylo Usyk, Soyoung Kwak, Chengwei Peng, Kenneth S. Hu, Paul E. Oberstein, Michelle Krogsgaard, Huilin Li, Richard B. Hayes, Jiyoung Ahn

**Affiliations:** 1 Department of Population Health, NYU Grossman School of Medicine, New York, New York, USA; 2 NYU Laura and Isaac Perlmutter Cancer Center, New York, New York, USA; 3 Northwestern University Feinberg School of Medicine, Chicago, Illinois, USA; 4 Department of Radiation Oncology, NYU Grossman School of Medicine, New York, New York, USA; 5 Department of Pathology, NYU Grossman School of Medicine, New York, New York, USA; University of Michigan, Ann Arbor, Michigan, USA

**Keywords:** tumor tissue microbiome, early stage, pancreatic cancer, survival

## Abstract

The pancreas tumor microbiota may influence tumor microenvironment and influence survival in early-stage pancreatic ductal adenocarcinoma (PDAC); however, current studies are limited and small. We investigated the relationship of tumor microbiota to survival in 201 surgically resected patients with localized PDAC (Stages I–II), from The Cancer Genome Atlas (TCGA) and International Cancer Genome Consortium (ICGC) cohorts. We characterized the tumor microbiome using RNA-sequencing data. We examined the association of the tumor microbiome with overall survival (OS), via meta-analysis with the Cox PH model. A microbial risk score (MRS) was calculated from the OS-associated microbiota. We further explored whether the OS-associated microbiota is related to host tumor immune infiltration. PDAC tumor microbiome α- and β-diversities were not associated with OS; however, 11 bacterial species, including species of *Gammaproteobacteria*, confirmed by extensive resampling, were significantly associated with OS (all *Q* < 0.05). The MRS summarizing these bacteria was related to a threefold change in OS (hazard ratio = 2.96 per standard deviation change in the MRS, 95% confidence interval = 2.26–3.86). This result was consistent across the two cohorts and in stratified analyses by adjuvant therapy (chemotherapy/radiation). Identified microbiota and the MRS also exhibited association with memory B cells and naïve CD4^+^ T cells, which may be related to the immune landscape through BCR and TCR signaling pathways. Our study shows that a unique tumor microbiome structure, potentially affecting the tumor immune microenvironment, is associated with poorer survival in resected early-stage PDAC. These findings suggest that microbial mechanisms may be involved in PDAC survival, potentially informing PDAC prognosis and guiding personalized treatment strategies.

**IMPORTANCE:**

Much of the available data on the PDAC tumor microbiome and survival are derived from relatively small and heterogeneous studies, including those involving patients with advanced stages of pancreatic cancer. There is a critical knowledge gap in terms of the tumor microbiome and survival in early-stage patients treated by surgical resection; we expect that advancements in survival may initially be best achieved in these patients who are treated with curative intent.

## INTRODUCTION

Pancreatic ductal adenocarcinoma (PDAC) ranks third in global cancer-related mortality, exhibiting a disheartening 13% 5-year overall survival (OS) (1). Surgical intervention is a potentially curative treatment option for early-stage PDAC (Stages I to II) (2, 3); yet, the 5-year survival rate after surgical resection of these tumors remains low, varying from 10% to 40% (4–6). Differences in mortality cannot be completely explained by disease stage or clinicopathological factors (7, 8), suggesting that other factors may contribute to survival. Thus, there is a pressing need to identify factors that enable prognostic stratification and potentially guide risk-stratified treatment strategies for pancreatic cancer (9).

Growing evidence suggests that the tumor microbiome exerts an influence on pancreatic cancer survival. Investigations in tumors of pancreatic cancer patients identified a distinct tumor microbiome composition and predominant bacterial communities in long-term PDAC survivors (>5 years), compared to short-term survivors (<5 years) (10, 11). Studies by Ghaddar et al. (12) also identified a subset of pancreas tumors with bacteria that were associated with cell type-specific gene expression patterns and pathways, T-cell immune activity, and poorer prognosis, whereas investigations by Chakladar et al. (13) demonstrated an overlap between smoking-related and metastasis-related tumor microbiota, both linking to immunosuppressive pathways in PDAC patients. Much of the available data on the PDAC tumor microbiome and survival are derived from relatively small and heterogeneous studies, including those involving patients with advanced stages of pancreatic cancer. There is a critical knowledge gap in terms of the tumor microbiome and survival in early-stage patients treated by surgical resection; we expect that advancements in survival may initially be best achieved in these patients who are treated with curative intent.

We investigated the relationships of the tumor microbiome with OS (the primary outcome) and relapse-free survival (RFS, the secondary outcome) in 201 Stages I–II PDAC patients treated by tumor resection and with no prior treatment. We also assessed whether the survival-associated microbiota is linked to tumor immune infiltration and gene expression in these patients, using data from The Cancer Genome Atlas (TCGA) and the International Cancer Genome Consortium (ICGC).

## RESULTS

### Patient characteristics

The two cohorts consisted of 201 early-stage PDAC participants with a median age of 66 years (range: 34–90 years, Table 1); a high proportion of patients were male (55.2%), White (89.6%), and current or former smokers (49.7%) and had Stage IIB (70.1%) or histological Grade 2 (56.7%) tumors. During the follow-up period (median: 15.3 months), 144 patients (71.6%) experienced a relapse event, and 118 patients (58.7%) died. Older age at diagnosis and Stage IIB were associated with poorer prognosis in the two cohorts (Table 1). In TCGA, 103 patients (73.6%) and 39 patients (27.9%) were treated, respectively, with adjuvant chemotherapy and radiation therapy (treatment data were unavailable for ICGC; Table S1).

**TABLE 1:** OS and RFS of Stages I–II PDAC patients in the meta-analysis of TCGA and ICGC cohorts, by selected patient characteristics

Characteristics	Combined cohorts (*N* = 201)						
	N (%)	OS			RFS		
		HR (95% CI)^*c*^	*P*-meta^*d*^	*P*-het	HR (95% CI)^*c*^	*P*-meta^*d*^	*P*-het
Age^*a*^ (range)	66 (34, 90)	1.03 (1.01–1.05)	0.004	0.772	1.02 (1.00–1.03)	0.049	0.499
Gender							
Female	90 (44.8%)	Reference			Reference		
Male	111 (55.2%)	0.89 (0.62–1.28)	0.530	0.748	1.02 (0.73–1.41)	0.927	0.807
Race							
White	180 (89.6%)	Reference			Reference		
Others	17 (8.5%)	0.64 (0.29–1.42)	0.273	0.997	0.74 (0.40–1.34)	0.317	0.973
Not reported	4 (2.0%)						
Cigarette smoking							
Never	75 (37.3%)	Reference			Reference		
Current	23 (11.4%)	1.06 (0.50–2.24)	0.880	0.231	1.03 (0.51–2.10)	0.932	0.214
Former	77 (38.3%)	0.85 (0.56–1.31)	0.467	0.745	0.90 (0.61–1.32)	0.582	0.926
Not reported	26 (12.9%)						
Tumor stage							
Resectable (I/IIA)	60 (29.9%)	Reference			Reference		
Borderline resectable (IIB)	141 (70.1%)	1.73 (1.12–2.69)	0.014	0.877	1.50 (1.02–2.19)	0.039	0.486
Tumor grade							
G1	20 (10.0%)						
G2	114 (56.7%)	Reference^*f*^			Reference^*f*^		
G3	64 (31.8%)	1.29 (0.87–1.91)	0.203	0.635	1.34 (0.94–1.92)	0.104	0.998
G4	3 (1.5%)						
Chemotherapy^*b*^							
No	29 (14.4%)	Reference			Reference		
Yes	103 (51.2%)	0.27 (0.16–0.46)	7.50E-07		0.40 (0.25–0.64)	1.42E-04	
Not reported	69 (34.3%)						
Radiation therapy^*b*^							
No	88 (43.8%)	Reference			Reference		
Yes	39 (19.4%)	0.41 (0.23–0.73)	0.002		0.60 (0.38–0.93)	0.024	
Not reported	74 (36.8%)						
Vital status							
Alive	83 (41.3%)						
Deceased	118 (58.7%)						
Relapse status							
No	57 (28.4%)						
Yes	144 (71.6%)						
OS in months^*e*^ (range)	15.3 (0–76.2)						
RFS in months^*e*^ (range)	12.1 (0–73.0)						

^*a*^ For age as a continuous variable: HR per 1 year of age.

^*b*^ Chemotherapy and radiation therapy data were available for TCGA only.

^*c*^ HR, Hazard ratio calculated by Cox regression. If the HR is >1, it indicates that the group of interest has a shorter survival than the reference group, and if the HR is <1, it indicates that the group of interest has a longer survival than the reference group.

^*d*^ *P*-meta, *P* value from random effects meta-analysis of the TCGA and ICGC cohorts shows the association between selected patient characteristics and PDAC survival; separate cohort results for these characteristics are presented in Table S1.

^*e*^ Follow-up duration months for OS and RFS in combined cohorts.

^*f*^ HR (95% CI) compared G3 and G4 combined as G2 as reference group.

### Tumor microbiome and OS and RFS

There were no significant differences observed in the α-diversity or β-diversity of the tumor microbiome with respect to cigarette smoking status, tumor stage (I/IIA vs. IIB), tumor grade (G1, G2, and G3), administration of chemotherapy, or radiation therapy (Fig. S1 to S4). Furthermore, no significant differences were observed in α-diversity or β-diversity with respect to survival (OS, Fig. S5) or relapse (RFS, Fig. S6).

**Fig 1:**
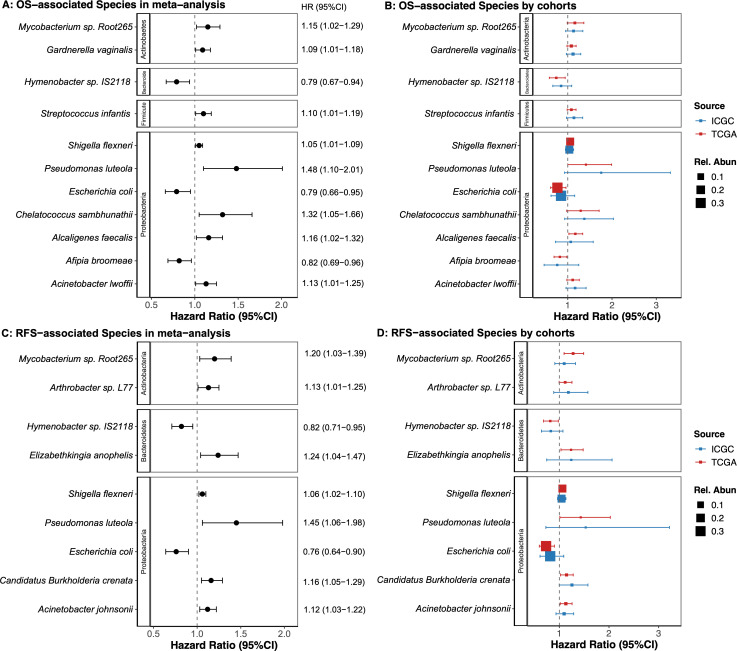
Tumor microbial species and survival in early-stage PDAC. The association of tumor microbial species with early-stage PDAC overall survival (OS) in (A) random-effects meta-analysis of the TCGA and ICGC cohorts, and (B) by individual cohort. The association of tumor microbial species with early-stage PDAC relapse-free survival (RFS) in (C) random-effects meta-analysis of the TCGA and ICGC cohorts, and (D) by individual cohort. All standard Cox hazard ratios (HR) were adjusted for age, sex, tumor stage, and cigarette smoking (HR <1, better survival; HR >1 poorer survival). Complete results for all tumor microbial species are presented in Supplemental Table 2. The size of the points indicates relative abundance, and the color of the points, in B and D, indicates each study cohort.

**Fig 2:**
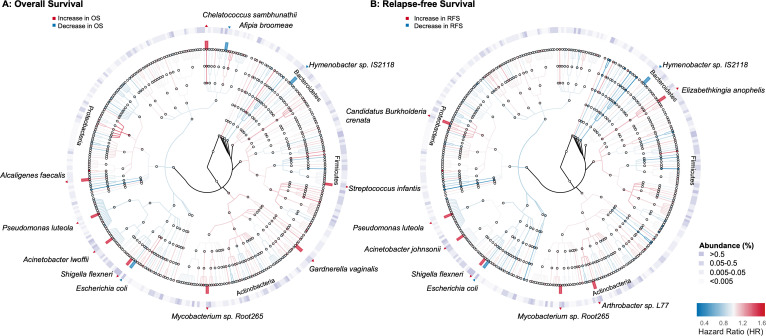
Cladogram representation of tumor microbiome species associated with PDAC Overall and Relapse-free Survival. The color of nodes and branches represents estimates of PDAC survival, based on the Hazard Ratio (HR) in Cox regressions, ranging from HR <1, in blue, to HR>1, in red. Eleven OS-associated species (Panel A) and nine RFS-associated species (Panel B) are highlighted and labeled on the outer rings, respectively. The intensity of magenta shading corresponds to the relative abundance of bacterial species. A total of 355 species are included in the cladogram, representing taxa with at least two sequences in at least 10% of participants and with mean relative abundance ≥0.001%. The cladogram was created using GraPhlAn (14).

By applying Cox proportional hazard (PH) models with repeated subsampling, we identified 11 bacterial taxa that are associated with OS, with >60% confirmation in repeated iterations of the meta-analysis (all *Q* < 0.05, Fig. 1A and B; Table S2). A greater abundance of several bacterial species was associated with poorer OS (hazard ratio [HR] > 1); these species included *Acinetobacter lwoffii*, *Pseudomonas luteola*, and *Shigella flexneri* in the *Gammaproteobacteria* class, *Alcaligenes faecalis* in the *Betaproteobacteria* class, *Chelatococcus sambhunathii* in the *Alphaproteobacteria* class, *Gardnerella vaginalis* and *Mycobacterium* sp. Root265 in the *Actinobacteria* phylum, and *Streptococcus infantis* in the *Firmicutes* phylum. Conversely, a higher abundance of *Escherichia coli* in the *Gammaproteobacteria* class, *Afipia broomeae* in the *Alphaproteobacteria* class, and *Hymenobacter* sp. IS2118 in the *Bacteroidetes* phylum was associated with better OS (HR < 1). Similarly, the abundance of nine bacterial species was associated with RFS (Fig. 1C and D). Notably, *H*. sp. IS2118 and *E. coli* were associated with better OS and RFS, whereas *M*. sp. Root265, *P. luteola*, and *S. flexneri* were associated with poorer OS and RFS. The phylogenetic relationships among bacteria associated with OS and RFS are shown in Fig. 2; Table S2. Additional adjustment for treatment (in TCGA only) revealed no substantial differences, with respect to treatment, in tumor bacteria-associated OS or RFS (Fig. S7).

### MRS and overall and relapse-free survival

**Fig 3:**
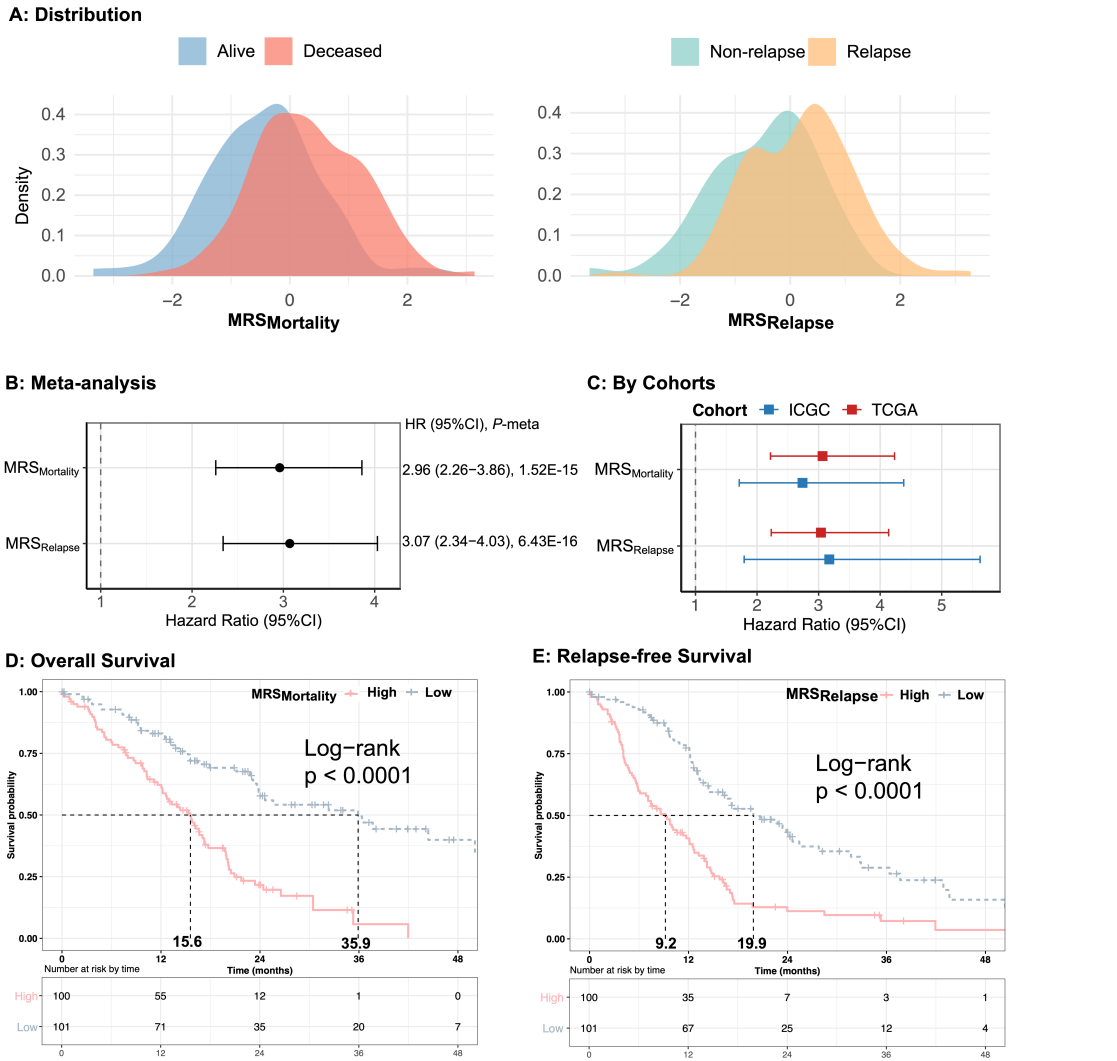
Microbial Risk Score (MRS) with overall survival and relapse-free survival in early-stage PDAC. (A) The density plot visualizes the distribution of z-score normalized MRSs. For MRSMortality, red represents alive patients, blue represents deceased patients; For MRSRelapse, yellow represents relapsed patients, and green represents non-relapsed patients in combined cohorts. Association of the MRSs with early-stage PDAC OS and RFS in the randomeffects meta-analysis (B) of the TCGA and ICGC cohorts, and (C) by individual cohort. The color of the points in C indicates each study cohort. MRSs were calculated as a weighted sum of clrnormalized abundance of OS-associated and RFS-associated bacterial species separately, with weights assigned according to their effect sizes determined via the Cox proportional hazard model with an adjustment for age sex, tumor stage, and cigarette smoking. Kaplan-Meier estimates the survival probability of MRSMortality with overall survival (D) and MRSRelapse with relapse-free survival (E) in combined cohorts. Pink represents the high-risk patients, and blue represents the low-risk patients, based on the median summary-weighted MRS indexes for OS-associated species and RFS-associate species, separately. The dotted line represents the median survival time of each group. The P-value was calculated from analysis of the deviance tables for two Cox model fits by log partial likelihood.

Recognizing that the microbiota are a complex ecosystem often composed of various subcommunities related to different traits, we constructed a microbial risk score (MRS) (15) to summarize the disease-specific microbial profile and to investigate its association with survival. Specifically, our MRS consists of the 11 bacteria for OS and nine species for RFS that showed association with OS and RFS, respectively, in >60% of the repeated iterations of the meta-analysis (all *Q* < 0.05). The MRS distributions differed among groups with differences in survival and relapse (Fig. 3A); a per unit increase in the MRS was associated with threefold increases in death (HR = 2.96) and relapse (HR = 3.07) (Fig. 3B). The MRS findings were consistent in both cohorts (HR = 3.06, 95% confidence interval [CI]: 2.22–4.23, *P* = 1.10E−11 in TCGA; HR = 2.74, 95% CI: 1.71–4.38, *P* = 2.70E−05 in ICGC; Fig. 3C), as also shown by Kaplan–Meier analysis (log-rank *P* < 0.0001; Fig. 3D and E), with a median survival time of 20**‒**36 months for patients with MRS less than the median versus 9**‒**16 months for patients with MRS greater than the median. The results of the Kaplan–Meier analyses were also similar for the separate cohorts, although not all findings remained individually statistically significant (Fig. S8). Associations of MRSs with survival remained consistent across the strata of cigarette smoking, tumor stage, and tumor grade (Fig. S9 and S10), as well as treatment (in TCGA only; Fig. S11).

### Tumor tissue microbiome and the immune microenvironment

**Fig 4:**
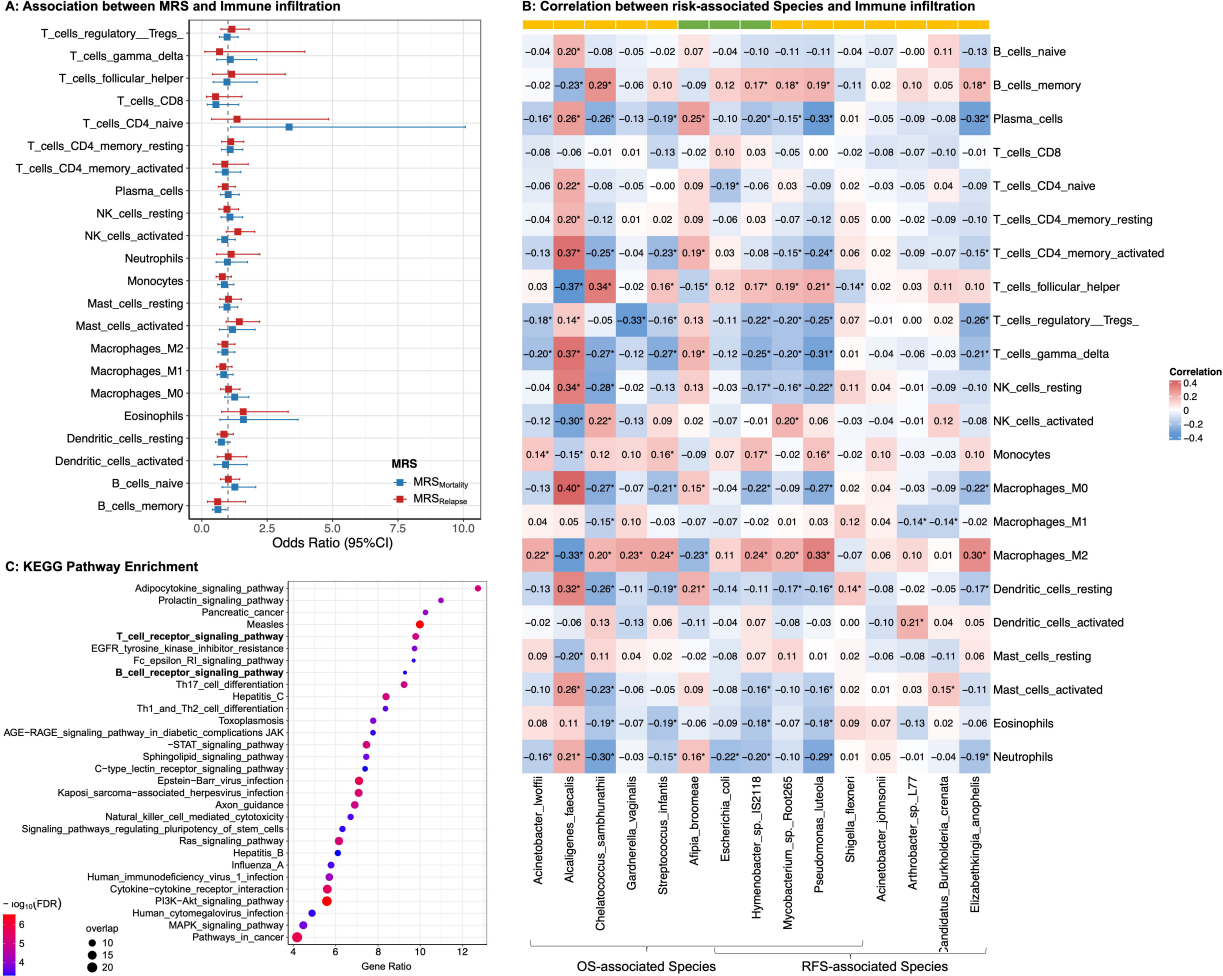
Tumor tissue microbiome and immune infiltration in early-stage PDAC. (A) Association analyses on MRSs and 22 immune cells. A generalized logistic regression was constructed for the binary quantification (low as 0, high as 1) of immune infiltrates (based on median) and the continuous variables of MRSs adjusted for age, sex, tumor stage, and cigarette smoking in random-effects meta-analysis of TCGA and ICGC cohorts. All measures were categorized based on their median of immune variables. Blue represents MRSMortality and Red represents MRSRelapse. (B) Correlation analyses on tumor tissue microbiota and 22 immune cells. The X-axis represents the OS- /RFS-associated species, and the Y-axis represents the immune infiltration. The color of the square indicates the Spearman correlation between the microbial node and the immune node. *p<0.05. The color of the bar indicates the Hazard Ratio (HR) of Species in Cox proportional hazards models on survival, ranging from HR <1, in green, to HR>1, in yellow. (C) KEGG pathway enrichment for microbial-correlated genes. The color and size of each bubble denote enrichment significance, and the number of genes enriched in the functional category, respectively.

The 11 OS-associated bacteria tended to belong to modules and pathways in energy metabolism and environmental information processing (Fig. S12; Table S3), suggesting the potential for functional differences of microbial association with survival. We further found that the MRS_Mortality_ was associated with an increased presence in tumors of naïve CD4^+^ T cells (OR = 3.34, 95% CI: 1.11–10.07, *P* = 0.033) and a lower presence of memory B cells (OR = 0.62, 95% CI: 0.41–0.92, *P* = 0.019; Fig. 4A). Notably, OS-associated *A. faecalis* (HR > 1) correlated positively with naïve CD4^+^ T cells and inversely with memory B cells (Spearman correlation: *r* = 0.22, *P* = 2.05E−03; *r* = −0.23, *P* = 8.47E−04; Fig. 4B). We also observed that the abundance of OS-associated bacterial species such as *A. faecalis* was inversely correlated with the expression levels of immunity genes in the B-cell receptor (BCR) signaling pathway, such as *SYK* (*r* = 0.22, *P* = 8.59E−03 in TCGA; *r* = 0.37, *P* = 4.44E−03 in ICGC, Table S4). Moreover, we identified microbial-correlated genes exhibiting significant enrichment in the BCR, T-cell receptor (TCR), and adipocytokine signaling pathways (Fig. 4C), reflecting the potential role of the OS-associated microbiome in the natural antibacterial response and host immune pathways.

## DISCUSSION

In this study, we found that survivorship differed by the abundance of 11 bacterial species in the tumors of early-stage, resected, PDAC patients. MRS constructed from 11 and nine bacteria, respectively, were associated with threefold increases in mortality and relapse, per unit increase in the MRS. Differentials in the MRS were related to differences of, on average, many months of survival for this high-fatality disease, suggesting that the tumor microbiome may have potential, with further refinement, to serve as a robust prognostic marker in early-stage PDAC. High-risk patients, defined by microbial profiles, may also benefit from targeted measures and personalized treatment strategies to enhance survival after localized PDAC surgery (16), with potential implications for clinical decision-making.

The nearly threefold differentials in survival revealed by the microbial community-based MRS (15) suggests that the ecologic characteristics of the tumor microbiome may be more important for prognosis than the presence of single bacterial species or compared to traditional tumor staging and other patient characteristics. Since the highest-risk patients will relapse shortly after surgery, it may be advantageous to adopt a neoadjuvant therapy approach or intensive chemotherapy in this subcohort. A microbial imbalance, or dysbiosis, is related to several disease conditions or poor health outcomes (17–23), including cancers, obesity, diabetes, and inflammatory diseases. Our results reflect that summary MRS leads to significant survival differentials in early-stage PDAC, which may advance the clinical relevance of specific microbial communities at the primary tumor site.

Notably, our study revealed that some species of the class *Alphaproteobacteria*, *Betaproteobacteria*, and *Gammaproteobacteria* were associated with OS. These findings are generally consistent with previous studies indicating that the tumor microbiome in *Gammaproteobacteria* class is related to PDAC response to chemotherapeutic gemcitabine (24), metastasis (13), and oncogenesis in both human and animal models (25). Furthermore, our study identifies new tumor microbial species associated with early-stage PDAC survival that were not observed in previous PDAC research (26). Of these taxa, unfavorable species relevant to poor prognosis comprised *S. infantis* (27, 28), members in *Acinetobacter* genus (*A. johnsonii*, *A. lwoffii*) (29), *and P. luteola* (30), which are potential opportunistic pathogenic bacteria that may cause serious infections. Further connections between identified species and microbial functional modules in energetic metabolism are aligned with previous observations on tumor progression (31) and long-term survival of PDAC (11).

Mechanistically, certain microbial taxa may influence PDAC through persistent inflammation, alterations of host immune response, metabolism regulation, or tumor microenvironment modulation (32). Pushalkar et al. demonstrated that microorganisms migrate from the gut to the pancreas in both human and mouse models (25); similarly, stool transfer from long-term survivors was shown to impede tumor growth in mouse models (11), suggesting that components of the gut microbiome might shape at a distance the tumor microbial community to impact PDAC outcomes (33).

Our observations concur with the growing body of evidence highlighting the significance of CD4^+^ T cells and B cells in PDAC (34–36). Specific bacteria, mainly *Gammaproteobacteria*, which affect human health worldwide, can manipulate B cells to their preference (37). In accordance, our findings suggest that unfavorable OS-associated *A. faecalis* is potentially linked to immune-suppressed T- and B-cell infiltrations, with implications for PDAC treatment strategies. This contrasts with the role of *Alcaligenes* residing in gut-associated lymphoid tissues (38), where bacteria can activate immune responses via their derived lipid A, a core part of lipopolysaccharides (LPS) that has been shown to activate dendritic cells and naïve B cells in the gut (39). However, our observations align with studies on extensively drug-resistant *A. faecalis* infections (40), where its cell wall component hyperactivates immune variants, leading to immunodeficiency and systemic inflammation in humans and mice (41). B-cell infiltration has been associated with favorable outcomes of PDAC through antitumor effects mediated by antibody secretion (42) and, conversely, shorter survival via immune suppression (36). The spatial distribution of cytotoxic T cells (i.e., CD4^+^ T cells) in proximity to PDAC cells correlates with increased overall patient survival (43). This emerging evidence underscores the complex role of T and B cells in PDAC and aligns with our findings, which showed that MRS and novel unfavorable OS-associated microbial species, including *A. faecalis*, were associated with increased naïve CD4^+^ T-cell and reduced memory B cell infiltration.

We provide additional evidence supporting previous research that the TCR and BCR signaling pathways are part of a supportive microenvironment in pancreatic cancer tumors (44). Survival-associated tumor microbiota may influence the PDAC immune landscape through key signaling molecules such as *SYK* (45) in the BCR and TCR signaling pathway, relating potentially to a role of tumor microbiome-linked immunity by TCR-mediated CD4^+^ T-cell activation (46) and BCR-regulated immune response (47) in the pathogenesis of PDAC (12). Previous studies have demonstrated that microbiota-derived LPS can increase the invasiveness of PDAC cells through metabolite signaling pathways (48, 49), leading to the production of inflammatory cytokines and immune suppression (25, 50). Our data also point to enrichment in adipocytokine and prolactin signaling pathways, highlighting the potential role of the tumor microbiome in inflammation and metabolism, with implications for T-cell and B cell-specific immunotherapy in clinical settings.

There are limitations in our study. First, from our observational study, limited mechanistic information on causal relationships could be obtained for the tumor microbiome, warranting further human interventional studies and experimental models (51). Second, there has been discussion on the human genomic contamination for the tumor microbiome characterization; however, these issues have been comprehensively addressed with an additional rigorous “exhaustive” filtering process (52) using GRCh38 (53), T2T-CHM13 (including chromosome Y) (54), and human pangenome (Human Pangenome Reference Consortium [HPRC]) (55). Lastly, the treatment data from the ICGC cohort could not be accessed, limiting the comparison of the cohorts on this factor; however, primary pancreas treatment is standardized in general by tumor stage (56). The methodological rigor of this research is also evidenced by (i) our large pooled sample size, allowing for the study of early-stage resected PDAC, without the interference of heterogeneity from prior neoadjuvant therapy, and interplay with clinicopathological factors; (ii) two multicenter well-structured cohort studies, TCGA and ICGC, relying on tumor tissue samples collected during surgery for subsequent follow-up for overall and RFS; and (iii) the RNA-sequencing (RNA-seq) approach for comprehensive assignment of taxonomy and for assessment of host gene pathways and immune modules.

Our study provides insights into a unique tumor microbiome structure and survival in resected early-stage PDAC from two well-structured international cohorts and for understanding the role of the T-cell and B-cell immune microenvironment in this process. By identifying prognostic tumor microbial markers, we indicate avenues for understanding underlying mechanisms and guiding personalized treatment strategies in the clinical management of early-stage resected PDAC.

## MATERIALS AND METHODS

### Study population and inclusion criteria

#### Cohort

This study is based on two international multi-center, well-established cohorts (seeFig. S13 for workflow).

TCGA (57) is a comprehensive resource and coordinated effort funded and backed by the NCI; it contains molecular characteristic information on over 20,000 primary cancer and matched normal samples spanning 33 cancer types to explore genomic changes and molecular basis (58). From 2006 to 2015, 11,160 patients from 20 collaborating institutions generated standard clinical data resources with high-quality survival outcomes (59). All data for 178 pancreatic cancer patients are available in TCGA cohort (60).

The ICGC (61) is a global initiative from 84 worldwide cancer projects, which aims to elucidate genomic changes by coordinating large-scale cancer genome studies (62). Diverse participants from 20 countries engaged between 2008 and 2013 and concluded the 25K Project in 2018 with clinical outcomes (63). All data from 78 pancreatic cancer patients collected from the Australian Pancreatic Cancer Genome Initiative are available in the ICGC Cohort (64).

#### Inclusion criteria

The following restricted criteria were used to identify studies included in our analysis: (i) the presence of surgically resected primary PDAC, (ii) classification within Stages I to II according to the American Joint Committee on Cancer Cancer Staging Manual, (iii) no prior neoadjuvant treatment before surgery, (iv) evaluable primary tumors were obtained via surgical resection, and (v) comprehensive clinical documentation. This resulted in a total of 140 and 63 Stages I/II primary surgical resected, non-pretreated PDAC patients in TCGA and ICGC, respectively, for further microbiome analysis.

#### Outcome

The primary outcome was OS, and the secondary outcome was RFS. OS was the period from the date of diagnosis until the date of death from any cause. Patients who were alive and free of these events were censored at the last follow-up (59). RFS was the time interval between the date of diagnosis and the date of local relapse, regional relapse, distant metastases (liver or non-liver), or death (all death), whichever occurs first (65).

### Tumor microbiome RNA-seq assay and data processing

#### RNA-seq assay

RNA was extracted from tumor tissue specimens, quantified and converted to mRNA libraries. Libraries were sequenced, and FASTQ files were generated according to methods described in previous TCGA (60) and ICGC (66) studies.

#### Microbiome data processing

##### Data filtering

Consistent processing was performed for all raw sequencing data using SHOGUN (67). FASTQ RNA sequencing reads were trimmed to remove bases that had a PHRED quality score of 25 or lower using Trim Galore.

##### Exhaustive *host depletion*

The quality-controlled paired-end data were processed with an additional rigorous “Exhaustive” filtering process (52) to identify all genomic regions shared with GRCh38 (53), T2T-CHM13 (including chromosome Y) (54), and human pangenome (HPRC) (55) references of 47 phased, diploid assemblies from a cohort of genetically diverse individuals. The total reads for all samples are shown in Fig. S14. The human sequence removal data were subsequently aligned with Bowtie2 with the National Center for Biotechnology Information (NCBI) RefSeq representative prokaryotic genome collection (release 82) (68).

##### Taxonomic assignment

Per strain coverage was calculated via the default pipeline SHOGUN profiles for the microbiome quantification (67). Sufficient reads mapped to a single reference genome and multiple reference genomes were assigned at the species level and lowest-common ancestor according to the NCBI taxonomy (69), respectively.

##### Batch correction

Finally, batch effect removal was performed for two tumor microbiome data in TCGA and ICGC via conditional quantile regression (ConQuR) (70).

### Gene and immune data processing

Available PDAC-normalized gene expression patterns were obtained from the Xena platform (71) and were initially derived from RNA-seq assays described in TCGA and ICGC (60, 66). The infiltration levels of 22 immune cell types were further estimated via a robust enumeration method (cell-type identification by estimating relative subsets of RNA transcripts [CIBERSORT]) for both cohorts (72, 73).

### Statistical analysis

Baseline characteristics were compared in terms of α-diversity and β-diversity. The α-diversity values (Chao1, Shannon, and Simpson indices) were computed to examine the differences via the “vegan” R package and were compared via the Wilcoxon rank test (two groups) or Kruskal‒Wallis test (more than two groups) and linear regression adjusted for age, sex, tumor stage, and cigarette smoking. Cox PH models were used to determine whether α-diversity was associated with OS and RFS, adjusting for age, sex, tumor stage, and cigarette smoking. Moreover, β-diversity (Jensen‒Shannon divergence) was calculated via the “phyloseq” R package as the average distance in 10,000 permutations of random pairings of tumor samples, where the thresholds were determined on the basis of rarefaction curves (Fig. S15), and significance was assessed via permutational multivariate analysis of variance, adjusting for the covariates listed above on time-to-event data.

Taxa with a prevalence of less than 10% in at least two sequences and a mean relative abundance of less than 0.001% were removed, resulting in the inclusion of 355 species. Taxonomic abundance was normalized via scale-invariant centered log-ratio (clr) transformation after adding a pseudo count in microbiome data analysis. Standard Cox PH models for all tested taxa were performed for OS and RFS, adjusting for the covariates listed above on time-to-event data. First, cohort-specific estimates of differential abundance by species were obtained. Then, cohort-specific estimates and their corresponding standard errors were used to perform random-effects meta-analysis to generate the pooled estimates for the two cohorts.

To mitigate the randomness in species selection, we employed a stability selection strategy using a repeated subsampling approach. A repeated subsampling procedure was also performed so that for each of 100 iterations, 90% of the data were randomly subsampled (74), and a meta-analysis of the Cox PH model was performed. Species significantly associated with OS or RFS were identified if they appeared in at least 60% of the iterations, with the recommended threshold (75, 76), confirming the stability of our findings (Fig. S13). We further confirmed the robustness of selected bacterial species based on repeated a subsampling procedure, using a permutation test with 500 iterations. The *P* value for *j*th species was calculated as $\frac{\sum_{i}^{500}I\left(P_{ij}\geq O_{j}\right)+1}{501}$, where I(⋅) is the indicator function, $O_{j}$ was the frequency of *j*th species identified (i.e., with a *P* meta of <0.05 in Cox PH model) using the original cohorts, and $P_{ij}$ was the frequency of *j*th species identified using the permuted data in *i*th iteration. The *Q* value was then calculated adjusting for the false discovery rate across all species.

Furthermore, by building on the concept of a polygenic risk score used productively in the genomic research (77), we constructed MRSs (15) to summarize the microbial profiles for PDAC prognosis prediction in terms of OS and RFS. For each outcome, its MRS was calculated as a weighted sum of the clr-normalized abundance of selected bacterial species, with weights assigned according to their effect sizes for individual species. Random-effects meta-analysis and Cox PH models were performed to assess the effects of MRS on OS and RFS. Patients were stratified into high-risk and low-risk groups based on the median MRSs. Kaplan–Meier curves and the log-rank test were then used to estimate the OS and RFS curves and test their differences between the high-risk and low-risk patients, respectively.

A generalized logistic regression was constructed for the binary abundance (low as 0, high as 1) of immune variables and the continuous variables of MRSs adjusted for age, sex, tumor stage, cigarette smoking, and cohort in two combined cohorts. All measures were categorized based on their median of immune variables. We used Spearman’s correlation to examine the relation between clr-normalized taxa, microbial modules, microbial pathways, immune infiltrations, and immune genes. These immune genes and survival-associated microbiota were calculated with *P* < 0.05. Kyoto Encyclopedia of Genes and Genomes (KEGG) enrichment analysis was performed using WebGestalt (78). All statistical analyses were conducted using R version 3.6.1. Figures were generated using the ggplot2 packages. For all statistical tests, a two-sided *P* < 0.05 was considered statistically significant.

## Data Availability

The data are available in a public, open-access repository. All raw RNA-sequencing data and demographic and clinical patient variables used in TCGA study can be downloaded directly from the GDC data portal (https://portal.gdc.cancer.gov/). The raw sequencing data for ICGC samples are available from the ICGC 25K Data Portal and the European Nucleotide Archive under the study identifier PRJEB42013 . All the study samples were from the “pancreatic ductal adenocarcinoma” datasets with “RNA-seq” as the experimental strategy.

## SUPPLEMENTAL MATERIAL

Supplemental Tables and Figures msystems.01229-24-s0001.docx Tables S1-S4 and Figures S1-S15.

ASM does not own the copyrights to Supplemental Material that may be linked to, or accessed through, an article. The authors have granted ASM a non-exclusive, world-wide license to publish the Supplemental Material files. Please contact the corresponding author directly for reuse.

## References

[1] Siegel RL, Giaquinto AN, Jemal A. 2024. Cancer statistics, 2024. CA A Cancer J Clinicians 74:12–49. doi:10.3322/caac.21820 38230766

[2] Siegel RL, Miller KD, Wagle NS, Jemal A. 2023. Cancer statistics, 2023. CA A Cancer J Clinicians 73:17–48. doi:10.3322/caac.21763 36633525

[3] Daamen LA, Groot VP, Besselink MG, Bosscha K, Busch OR, Cirkel GA, van Dam RM, Festen S, Groot Koerkamp B, Haj Mohammad N, et al.. 2022. Detection, treatment, and survival of pancreatic cancer recurrence in the Netherlands: a nationwide analysis. Ann Surg 275:769–775. doi:10.1097/SLA.0000000000004093 32773631

[4] Khorana AA, Mangu PB, Berlin J, Engebretson A, Hong TS, Maitra A, Mohile SG, Mumber M, Schulick R, Shapiro M, Urba S, Zeh HJ, Katz MHG. 2017. Potentially curable pancreatic cancer: American society of clinical oncology clinical practice guideline update. J Clin Oncol 35:2324–2328. doi:10.1200/JCO.2017.72.4948 28398845

[5] Moletta L, Serafini S, Valmasoni M, Pierobon ES, Ponzoni A, Sperti C. 2019. Surgery for recurrent pancreatic cancer: is it effective? Cancers (Basel) 11:991. doi:10.3390/cancers11070991 31315222 PMC6679234

[6] Huang L, Jansen L, Balavarca Y, Babaei M, van der Geest L, Lemmens V, Van Eycken L, De Schutter H, Johannesen TB, Primic-Žakelj M, Zadnik V, Besselink MG, Schrotz-King P, Brenner H. 2018. Stratified survival of resected and overall pancreatic cancer patients in Europe and the USA in the early twenty-first century: a large, international population-based study. BMC Med 16:125. doi:10.1186/s12916-018-1120-9 30126408 PMC6102804

[7] Neoptolemos JP, Kleeff J, Michl P, Costello E, Greenhalf W, Palmer DH. 2018. Therapeutic developments in pancreatic cancer: current and future perspectives. Nat Rev Gastroenterol Hepatol 15:333–348. doi:10.1038/s41575-018-0005-x 29717230

[8] Kamisawa T, Wood LD, Itoi T, Takaori K. 2016. Pancreatic cancer. Lancet 388:73–85. doi:10.1016/S0140-6736(16)00141-0 26830752

[9] Singhi AD, Koay EJ, Chari ST, Maitra A. 2019. Early detection of pancreatic cancer: opportunities and challenges. Gastroenterology 156:2024–2040. doi:10.1053/j.gastro.2019.01.259 30721664 PMC6486851

[10] Huang Y, Zhu N, Zheng X, Liu Y, Lu H, Yin X, Hao H, Tan Y, Wang D, Hu H, Liang Y, Li X, Hu Z, Yin Y. 2022. Intratumor microbiome analysis identifies positive association between megasphaera and survival of chinese patients with pancreatic ductal adenocarcinomas. Front Immunol 13:doi doi:10.3389/fimmu.2022.785422 PMC8821101 35145519

[11] Riquelme E, Zhang Y, Zhang L, Montiel M, Zoltan M, Dong W, Quesada P, Sahin I, Chandra V, San Lucas A, et al.. 2019. Tumor microbiome diversity and composition influence pancreatic cancer outcomes. Cell 178:795–806. doi:10.1016/j.cell.2019.07.008 31398337 PMC7288240

[12] Ghaddar B, Biswas A, Harris C, Omary MB, Carpizo DR, Blaser MJ, De S. 2022. Tumor microbiome links cellular programs and immunity in pancreatic cancer. Cancer Cell 40:1240–1253. doi:10.1016/j.ccell.2022.09.009 36220074 PMC9556978

[13] Chakladar J, Kuo SZ, Castaneda G, Li WT, Gnanasekar A, Yu MA, Chang EY, Wang XQ, Ongkeko WM. 2020. The pancreatic microbiome is associated with carcinogenesis and worse prognosis in males and smokers. Cancers (Basel) 12:doi doi:10.3390/cancers12092672 PMC7565819 32962112

[14] Asnicar F, Weingart G, Tickle TL, Huttenhower C, Segata N. 2015. Compact graphical representation of phylogenetic data and metadata with GraPhlAn. PeerJ 3:e1029. doi:10.7717/peerj.1029 26157614 PMC4476132

[15] Wang C, Segal LN, Hu J, Zhou B, Hayes RB, Ahn J, Li H. 2022. Microbial risk score for capturing microbial characteristics, integrating multi-omics data, and predicting disease risk. Microbiome 10:121. doi:10.1186/s40168-022-01310-2 35932029 PMC9354433

[16] Bateni SB, Gingrich AA, Hoch JS, Canter RJ, Bold RJ. 2019. Defining value for pancreatic surgery in early-stage pancreatic cancer. JAMA Surg 154:e193019. doi:10.1001/jamasurg.2019.3019 31433465 PMC6704743

[17] Han MK, Zhou Y, Murray S, Tayob N, Noth I, Lama VN, Moore BB, White ES, Flaherty KR, Huffnagle GB, Martinez FJ, COMET Investigators. 2014. Lung microbiome and disease progression in idiopathic pulmonary fibrosis: an analysis of the COMET study. Lancet Respir Med 2:548–556. doi:10.1016/S2213-2600(14)70069-4 24767767 PMC4142525

[18] Nguyen CL, Markey KA, Miltiadous O, Dai A, Waters N, Sadeghi K, Fei T, Shouval R, Taylor BP, Liao C, et al.. 2023. High-resolution analyses of associations between medications, microbiome, and mortality in cancer patients. Cell 186:2705–2718. doi:10.1016/j.cell.2023.05.007 37295406 PMC10390075

[19] Leitao Filho FS, Alotaibi NM, Ngan D, Tam S, Yang J, Hollander Z, Chen V, FitzGerald JM, Nislow C, Leung JM, Man SFP, Sin DD. 2019. Sputum microbiome is associated with 1-year mortality after chronic obstructive pulmonary disease hospitalizations. Am J Respir Crit Care Med 199:1205–1213. doi:10.1164/rccm.201806-1135OC 30376356

[20] Peled JU, Gomes ALC, Devlin SM, Littmann ER, Taur Y, Sung AD, Weber D, Hashimoto D, Slingerland AE, Slingerland JB, et al.. 2020. Microbiota as predictor of mortality in allogeneic hematopoietic-cell transplantation. N Engl J Med 382:822–834. doi:10.1056/NEJMoa1900623 32101664 PMC7534690

[21] Qiao H, Tan X-R, Li H, Li J-Y, Chen X-Z, Li Y-Q, Li W-F, Tang L-L, Zhou G-Q, Zhang Y, Liang Y-L, He Q-M, Zhao Y, Huang S-Y, Gong S, Li Q, Ye M-L, Chen K-L, Sun Y, Ma J, Liu N. 2022. Association of intratumoral microbiota with prognosis in patients with nasopharyngeal carcinoma from 2 hospitals in China. JAMA Oncol 8:1301–1309. doi:10.1001/jamaoncol.2022.2810 35834269 PMC9284409

[22] Forslund K, Hildebrand F, Nielsen T, Falony G, Le Chatelier E, Sunagawa S, Prifti E, Vieira-Silva S, Gudmundsdottir V, Pedersen HK, et al.. 2015. Disentangling type 2 diabetes and metformin treatment signatures in the human gut microbiota. Nature New Biol 528:262–266. doi:10.1038/nature15766 PMC4681099 26633628

[23] Belda E, Voland L, Tremaroli V, Falony G, Adriouch S, Assmann KE, Prifti E, Aron-Wisnewsky J, Debédat J, Le Roy T, et al.. 2022. Impairment of gut microbial biotin metabolism and host biotin status in severe obesity: effect of biotin and prebiotic supplementation on improved metabolism. Gut 71:2463–2480. doi:10.1136/gutjnl-2021-325753 35017197 PMC9664128

[24] Geller LT, Barzily-Rokni M, Danino T, Jonas OH, Shental N, Nejman D, Gavert N, Zwang Y, Cooper ZA, Shee K, et al.. 2017. Potential role of intratumor bacteria in mediating tumor resistance to the chemotherapeutic drug gemcitabine. Science 357:1156–1160. doi:10.1126/science.aah5043 28912244 PMC5727343

[25] Pushalkar S, Hundeyin M, Daley D, Zambirinis CP, Kurz E, Mishra A, Mohan N, Aykut B, Usyk M, Torres LE, et al.. 2018. The pancreatic cancer microbiome promotes oncogenesis by induction of innate and adaptive immune suppression. Cancer Discov 8:403–416. doi:10.1158/2159-8290.CD-17-1134 29567829 PMC6225783

[26] Sexton RE, Uddin MH, Bannoura S, Khan HY, Mzannar Y, Li Y, Aboukameel A, Al-Hallak MN, Al-Share B, Mohamed A, Nagasaka M, El-Rayes B, Azmi AS. 2022. Connecting the human microbiome and pancreatic cancer. Cancer Metastasis Rev 41:317–331. doi:10.1007/s10555-022-10022-w 35366155 PMC8976105

[27] Pimenta F, Gertz RE, Park SH, Kim E, Moura I, Milucky J, Rouphael N, Farley MM, Harrison LH, Bennett NM, Bigogo G, Feikin DR, Breiman R, Lessa FC, Whitney CG, Rajam G, Schiffer J, da Gloria Carvalho M, Beall B. 2018. Streptococcus infantis, Streptococcus mitis, and Streptococcus oralis strains with highly similar cps5 loci and antigenic relatedness to serotype 5 pneumococci. Front Microbiol 9. doi:10.3389/fmicb.2018.03199 PMC6332807 30671034

[28] Li H, Wu X, Zeng H, Chang B, Cui Y, Zhang J, Wang R, Ding T. 2023. Unique microbial landscape in the human oropharynx during different types of acute respiratory tract infections. Microbiome 11:157. doi:10.1186/s40168-023-01597-9 37482605 PMC10364384

[29] Munoz-Price LS, Weinstein RA. 2008. Acinetobacter infection. N Engl J Med 358:1271–1281. doi:10.1056/NEJMra070741 18354105

[30] Doublet B, Robin F, Casin I, Fabre L, Le Fleche A, Bonnet R, Weill F-X. 2010. Molecular and biochemical characterization of the natural chromosome-encoded class A beta-lactamase from Pseudomonas luteola. Antimicrob Agents Chemother 54:45–51. doi:10.1128/AAC.00427-09 19884377 PMC2798517

[31] Guo W, Zhang Y, Guo S, Mei Z, Liao H, Dong H, Wu K, Ye H, Zhang Y, Zhu Y, Lang J, Hu L, Jin G, Kong X. 2021. Tumor microbiome contributes to an aggressive phenotype in the basal-like subtype of pancreatic cancer. Commun Biol 4:1019. doi:10.1038/s42003-021-02557-5 34465850 PMC8408135

[32] Cruz MS, Tintelnot J, Gagliani N. 2024. Roles of microbiota in pancreatic cancer development and treatment. Gut Microbes 16:2320280. doi:10.1080/19490976.2024.2320280 38411395 PMC10900280

[33] Panebianco C, Ciardiello D, Villani A, Maiorano BA, Latiano TP, Maiello E, Perri F, Pazienza V. 2022. Insights into the role of gut and intratumor microbiota in pancreatic ductal adenocarcinoma as new key players in preventive, diagnostic and therapeutic perspective. Semin Cancer Biol 86:997–1007. doi:10.1016/j.semcancer.2021.11.007 34838957

[34] Mahajan UM, Langhoff E, Goni E, Costello E, Greenhalf W, Halloran C, Ormanns S, Kruger S, Boeck S, Ribback S, et al.. 2018. Immune cell and stromal signature associated with progression-free survival of patients with resected pancreatic ductal adenocarcinoma. Gastroenterology 155:1625–1639. doi:10.1053/j.gastro.2018.08.009 30092175

[35] Hilmi M, Delaye M, Muzzolini M, Nicolle R, Cros J, Hammel P, Cardot-Ruffino V, Neuzillet C. 2023. The immunological landscape in pancreatic ductal adenocarcinoma and overcoming resistance to immunotherapy. Lancet Gastroenterol Hepatol 8:1129–1142. doi:10.1016/S2468-1253(23)00207-8 37866368

[36] Takahashi R, Macchini M, Sunagawa M, Jiang Z, Tanaka T, Valenti G, Renz BW, White RA, Hayakawa Y, Westphalen CB, Tailor Y, Iuga AC, Gonda TA, Genkinger J, Olive KP, Wang TC. 2021. Interleukin-1β-induced pancreatitis promotes pancreatic ductal adenocarcinoma via B lymphocyte-mediated immune suppression. Gut 70:330–341. doi:10.1136/gutjnl-2019-319912 32393543

[37] Nothelfer K, Sansonetti PJ, Phalipon A. 2015. Pathogen manipulation of B cells: the best defence is a good offence. Nat Rev Microbiol 13:173–184. doi:10.1038/nrmicro3415 25659322

[38] Obata T, Goto Y, Kunisawa J, Sato S, Sakamoto M, Setoyama H, Matsuki T, Nonaka K, Shibata N, Gohda M, Kagiyama Y, Nochi T, Yuki Y, Fukuyama Y, Mukai A, Shinzaki S, Fujihashi K, Sasakawa C, Iijima H, Goto M, Umesaki Y, Benno Y, Kiyono H. 2010. Indigenous opportunistic bacteria inhabit mammalian gut-associated lymphoid tissues and share a mucosal antibody-mediated symbiosis. Proc Natl Acad Sci U S A 107:7419–7424. doi:10.1073/pnas.1001061107 20360558 PMC2867693

[39] Liu Z, Hosomi K, Kunisawa J. 2022. Utilization of gut environment-mediated control system of host immunity in the development of vaccine adjuvants. Vaccine (Auckl) 40:5399–5403. doi:10.1016/j.vaccine.2022.07.031 35918205

[40] Li Y, Zhu Y, Zhou W, Chen Z, Moran RA, Ke H, Feng Y, van Schaik W, Shen H, Ji J, Ruan Z, Hua X, Yu Y. 2022. Alcaligenes faecalis metallo-β-lactamase in extensively drug-resistant Pseudomonas aeruginosa isolates. Clin Microbiol Infect 28:880. doi:10.1016/j.cmi.2021.11.012 34826621

[41] Wang L, Aschenbrenner D, Zeng Z, Cao X, Mayr D, Mehta M, Capitani M, Warner N, Pan J, Wang L, et al.. 2021. Gain-of-function variants in SYK cause immune dysregulation and systemic inflammation in humans and mice. Nat Genet 53:500–510. doi:10.1038/s41588-021-00803-4 33782605 PMC8245161

[42] Castino GF, Cortese N, Capretti G, Serio S, Di Caro G, Mineri R, Magrini E, Grizzi F, Cappello P, Novelli F, Spaggiari P, Roncalli M, Ridolfi C, Gavazzi F, Zerbi A, Allavena P, Marchesi F. 2016. Spatial distribution of B cells predicts prognosis in human pancreatic adenocarcinoma. Oncoimmunology 5:e1085147. doi:10.1080/2162402X.2015.1085147 27141376 PMC4839336

[43] Carstens JL, Correa de Sampaio P, Yang D, Barua S, Wang H, Rao A, Allison JP, LeBleu VS, Kalluri R. 2017. Spatial computation of intratumoral T cells correlates with survival of patients with pancreatic cancer. Nat Commun 8:15095. doi:10.1038/ncomms15095 28447602 PMC5414182

[44] Burger JA, Wiestner A. 2018. Targeting B cell receptor signalling in cancer: preclinical and clinical advances. Nat Rev Cancer 18:148–167. doi:10.1038/nrc.2017.121 29348577

[45] Vendel AC, Calemine-Fenaux J, Izrael-Tomasevic A, Chauhan V, Arnott D, Eaton DL. 2009. B and T lymphocyte attenuator regulates B cell receptor signaling by targeting Syk and BLNK. J Immunol 182:1509–1517. doi:10.4049/jimmunol.182.3.1509 19155498

[46] Alam MS, Gaida MM, Bergmann F, Lasitschka F, Giese T, Giese NA, Hackert T, Hinz U, Hussain SP, Kozlov SV, Ashwell JD. 2015. Selective inhibition of the p38 alternative activation pathway in infiltrating T cells inhibits pancreatic cancer progression. Nat Med 21:1337–1343. doi:10.1038/nm.3957 26479921 PMC4636461

[47] Pylayeva-Gupta Y, Das S, Handler JS, Hajdu CH, Coffre M, Koralov SB, Bar-Sagi D. 2016. IL35-producing B cells promote the development of pancreatic neoplasia. Cancer Discov 6:247–255. doi:10.1158/2159-8290.CD-15-0843 26715643 PMC5709038

[48] Dey P, Kimmelman AC, DePinho RA. 2021. Metabolic codependencies in the tumor microenvironment. Cancer Discov 11:1067–1081. doi:10.1158/2159-8290.CD-20-1211 33504580 PMC8102306

[49] Kiss B, Mikó E, Sebő É, Toth J, Ujlaki G, Szabó J, Uray K, Bai P, Árkosy P. 2020. Oncobiosis and microbial metabolite signaling in pancreatic adenocarcinoma. Cancers (Basel) 12:doi doi:10.3390/cancers12051068 PMC7281526 32344895

[50] Chen Y, Yang S, Tavormina J, Tampe D, Zeisberg M, Wang H, Mahadevan KK, Wu C-J, Sugimoto H, Chang C-C, Jenq RR, McAndrews KM, Kalluri R. 2022. Oncogenic collagen I homotrimers from cancer cells bind to α3β1 integrin and impact tumor microbiome and immunity to promote pancreatic cancer. Cancer Cell 40:818–834. doi:10.1016/j.ccell.2022.06.011 35868307 PMC9831277

[51] Sethi V, Vitiello GA, Saxena D, Miller G, Dudeja V. 2019. The role of the microbiome in immunologic development and its implication for pancreatic cancer immunotherapy. Gastroenterology 156:2097–2115. doi:10.1053/j.gastro.2018.12.045 30768986

[52] Sepich-Poore GD, McDonald D, Kopylova E, Guccione C, Zhu Q, Austin G, Carpenter C, Fraraccio S, Wandro S, Kosciolek T, Janssen S, Metcalf JL, Song SJ, Kanbar J, Miller-Montgomery S, Heaton R, Mckay R, Patel SP, Swafford AD, Korem T, Knight R. 2024. Robustness of cancer microbiome signals over a broad range of methodological variation. Oncogene 43:1127–1148. doi:10.1038/s41388-024-02974-w 38396294 PMC10997506

[53] Schneider VA, Graves-Lindsay T, Howe K, Bouk N, Chen H-C, Kitts PA, Murphy TD, Pruitt KD, Thibaud-Nissen F, Albracht D, et al.. 2017. Evaluation of GRCh38 and de novo haploid genome assemblies demonstrates the enduring quality of the reference assembly. Genome Res 27:849–864. doi:10.1101/gr.213611.116 28396521 PMC5411779

[54] Rhie A, Nurk S, Cechova M, Hoyt SJ, Taylor DJ, Altemose N, Hook PW, Koren S, Rautiainen M, Alexandrov IA, et al.. 2023. The complete sequence of a human Y chromosome. Nature New Biol 621:344–354. doi:10.1038/s41586-023-06457-y PMC10752217 37612512

[55] Liao W-W, Asri M, Ebler J, Doerr D, Haukness M, Hickey G, Lu S, Lucas JK, Monlong J, Abel HJ, et al.. 2023. A draft human pangenome reference. Nature New Biol 617:312–324. doi:10.1038/s41586-023-05896-x PMC10172123 37165242

[56] Wood LD, Canto MI, Jaffee EM, Simeone DM. 2022. Pancreatic cancer: pathogenesis, screening, diagnosis, and treatment. Gastroenterology 163:386–402. doi:10.1053/j.gastro.2022.03.056 35398344 PMC9516440

[57] Cancer Genome Atlas Research Network, Weinstein JN, Collisson EA, Mills GB, Shaw KRM, Ozenberger BA, Ellrott K, Shmulevich I, Sander C, Stuart JM. 2013. The Cancer Genome Atlas Pan-Cancer analysis project. Nat Genet 45:1113–1120. doi:10.1038/ng.2764 24071849 PMC3919969

[58] Cancer Genome Atlas Research Network. 2008. Comprehensive genomic characterization defines human glioblastoma genes and core pathways. Nature New Biol 455:1061–1068. doi:10.1038/nature07385 PMC2671642 18772890

[59] Liu J, Lichtenberg T, Hoadley KA, Poisson LM, Lazar AJ, Cherniack AD, Kovatich AJ, Benz CC, Levine DA, Lee AV, et al.. 2018. An integrated TCGA pan-cancer clinical data resource to drive high-quality survival outcome analytics. Cell 173:400–416. doi:10.1016/j.cell.2018.02.052 29625055 PMC6066282

[60] Cancer Genome Atlas Research Network, Cancer Genome Atlas Research Network. Electronic address: andrew_aguirre@dfci.harvard.edu. 2017. Integrated genomic characterization of pancreatic ductal adenocarcinoma. Cancer Cell 32:185–203. doi:10.1016/j.ccell.2017.07.007 28810144 PMC5964983

[61] International Cancer Genome Consortium, Hudson TJ, Anderson W, Artez A, Barker AD, Bell C, Bernabé RR, Bhan MK, Calvo F, Eerola I, et al.. 2010. International network of cancer genome projects. Nature New Biol 464:993–998. doi:10.1038/nature08987 PMC2902243 20393554

[62] Creixell P, Reimand J, Haider S, Wu G, Shibata T, Vazquez M, Mustonen V, Gonzalez-Perez A, Pearson J, Sander C, Raphael BJ, Marks DS, Ouellette BFF, Valencia A, Bader GD, Boutros PC, Stuart JM, Linding R, Lopez-Bigas N, Stein LD, Mutation Consequences and Pathway Analysis Working Group of the International Cancer Genome Consortium. 2015. Pathway and network analysis of cancer genomes. Nat Methods 12:615–621. doi:10.1038/nmeth.3440 26125594 PMC4717906

[63] Zhang J, Bajari R, Andric D, Gerthoffert F, Lepsa A, Nahal-Bose H, Stein LD, Ferretti V. 2019. The International Cancer Genome Consortium data portal. Nat Biotechnol 37:367–369. doi:10.1038/s41587-019-0055-9 30877282

[64] Scarlett CJ, Salisbury EL, Biankin AV, Kench J. 2011. Precursor lesions in pancreatic cancer: morphological and molecular pathology. Pathology (Phila) 43:183–200. doi:10.1097/PAT.0b013e3283445e3a 21436628

[65] Bonnetain F, Bonsing B, Conroy T, Dousseau A, Glimelius B, Haustermans K, Lacaine F, Van Laethem JL, Aparicio T, Aust D, et al.. 2014. Guidelines for time-to-event end-point definitions in trials for pancreatic cancer. Results of the DATECAN initiative (Definition for the Assessment of Time-to-event End-points in CANcer trials). Eur J Cancer 50:2983–2993. doi:10.1016/j.ejca.2014.07.011 25256896

[66] Bailey P, Chang DK, Nones K, Johns AL, Patch A-M, Gingras M-C, Miller DK, Christ AN, Bruxner TJC, Quinn MC, et al.. 2016. Genomic analyses identify molecular subtypes of pancreatic cancer. Nature New Biol 531:47–52. doi:10.1038/nature16965 26909576

[67] Hillmann B, Al-Ghalith GA, Shields-Cutler RR, Zhu Q, Knight R, Knights D. 2020. SHOGUN: a modular, accurate and scalable framework for microbiome quantification. Bioinformatics 36:4088–4090. doi:10.1093/bioinformatics/btaa277 32365167 PMC7359755

[68] Langmead B, Salzberg SL. 2012. Fast gapped-read alignment with Bowtie 2. Nat Methods 9:357–359. doi:10.1038/nmeth.1923 22388286 PMC3322381

[69] Hanson NW, Konwar KM, Hallam SJ. 2016. LCA*: an entropy-based measure for taxonomic assignment within assembled metagenomes. Bioinformatics 32:3535–3542. doi:10.1093/bioinformatics/btw400 27515739 PMC5181528

[70] Ling W, Lu J, Zhao N, Lulla A, Plantinga AM, Fu W, Zhang A, Liu H, Song H, Li Z, Chen J, Randolph TW, Koay WLA, White JR, Launer LJ, Fodor AA, Meyer KA, Wu MC. 2022. Batch effects removal for microbiome data via conditional quantile regression. Nat Commun 13:doi doi:10.1038/s41467-022-33071-9 PMC9477887 36109499

[71] Goldman MJ, Craft B, Hastie M, Repečka K, McDade F, Kamath A, Banerjee A, Luo Y, Rogers D, Brooks AN, Zhu J, Haussler D. 2020. Visualizing and interpreting cancer genomics data via the Xena platform. Nat Biotechnol 38:675–678. doi:10.1038/s41587-020-0546-8 32444850 PMC7386072

[72] Newman AM, Liu CL, Green MR, Gentles AJ, Feng W, Xu Y, Hoang CD, Diehn M, Alizadeh AA. 2015. Robust enumeration of cell subsets from tissue expression profiles. Nat Methods 12:453–457. doi:10.1038/nmeth.3337 25822800 PMC4739640

[73] Thorsson V, Gibbs DL, Brown SD, Wolf D, Bortone DS, Ou Yang T-H, Porta-Pardo E, Gao GF, Plaisier CL, Eddy JA, et al.. 2018. The immune landscape of cancer. Immunity 48:812–830. doi:10.1016/j.immuni.2018.03.023 29628290 PMC5982584

[74] Capanu M, Giurcanu M, Begg CB, Gönen M. 2023. Subsampling based variable selection for generalized linear models. Comput Stat Data Anal 184:107740. doi:10.1016/j.csda.2023.107740 37090139 PMC10118238

[75] Hofner B, Boccuto L, Göker M. 2015. Controlling false discoveries in high-dimensional situations: boosting with stability selection. BMC Bioinformatics 16:144. doi:10.1186/s12859-015-0575-3 25943565 PMC4464883

[76] Meinshausen N, Bühlmann P. 2010. Stability selection. J R Stat Soc Series B Stat Methodol 72:417–473. doi:10.1111/j.1467-9868.2010.00740.x

[77] Choi SW, Mak TS-H, O’Reilly PF. 2020. Tutorial: a guide to performing polygenic risk score analyses. Nat Protoc 15:2759–2772. doi:10.1038/s41596-020-0353-1 32709988 PMC7612115

[78] Liao Y, Wang J, Jaehnig EJ, Shi Z, Zhang B. 2019. WebGestalt 2019: gene set analysis toolkit with revamped UIs and APIs. Nucleic Acids Res 47:W199–W205. doi:10.1093/nar/gkz401 31114916 PMC6602449

