# Supplementary material for: Association of tumor microbiome with survival in resected early-stage PDAC
Source: mSystems. 2025 Feb 27;10(3):e01229-24. doi: 10.1128/msystems.01229-24 (PMC11915875; doi:10.1128/msystems.01229-24)
Supplement: Supplemental Tables and Figures — Tables S1-S4 and Figures S1-S15. [file msystems.01229-24-s0001.docx]

**Supplemental Online Content**

**Supplemental Table 1. Overall survival (OS) and Relapse-free survival (RFS) of stage I-II PDAC patients in the TCGA and ICGC cohorts, by selected patient characteristics.**

**Supplemental Table 2. Association of tumor microbial species with early-stage PDAC OS and RFS in the meta-analysis of TCGA and ICGC cohorts.**

**Supplemental Table 3. Association of tumor microbial modules and pathways with early-stage PDAC OS and RFS in the meta-analysis of TCGA and ICGC cohorts.**

**Supplemental Table 4. Spearman correlation of the clr-corrected abundance of survival-associated bacterial species and the differentially immunologically expressed genes with *P* < 0.05.**

**Supplemental Figure 1. Microbial community composition, by cigarette smoking.**

**Supplemental Figure 2. Microbial community composition, by tumor stage.**

**Supplemental Figure 3. Microbial community composition, by tumor grade.**

**Supplemental Figure 4. Microbial community composition, by chemotherapy** **and radiation therapy.**

**Supplemental Figure 5. Microbial community composition, by mortality.**

**Supplemental Figure 6. Microbial community composition, by relapse.**

**Supplemental Figure 7. Comparison of tumor microbial species with survival in TCGA cohort with adjustment of standard and additional covariates.**

**Supplemental Figure 8. Microbial Risk Score (MRS) with overall survival and relapse-free survival in early-stage PDAC by TCGA and ICGC.**

**Supplemental Figure 9. Associations between selected tumor microbial species and MRSs with early-stage PDAC OS and RFS, by cigarette smoking.**

**Supplemental Figure 10. Associations between selected tumor microbial species and MRSs with early-stage PDAC OS and RFS, by tumor stage and grade.**

**Supplemental Figure 11. Associations between selected tumor microbial species and MRSs with early-stage PDAC OS and RFS, by therapy strategies in TCGA.**

**Supplemental Figure 12. Contribution of bacterial species and functional modules and pathways in PDAC early-stage patients.**

**Supplemental Figure 13. The workflow for identifying prognostic microbial species across TCGA and ICGC cohorts.**

**Supplemental Figure 14. Bacterial sequence reads before and after host depletion.**

**Supplemental Figure 15. Bacterial rarefaction analysis curves between PDAC early-stage patients.**

This supplemental material has been provided by the authors to give readers additional information about their work.

**Supplemental Table 1. Overall survival (OS) and Relapse-free survival (RFS) of stage I-II PDAC patients in the TCGA and ICGC cohorts, by selected patient characteristics.**

| Characteristics | TCGA^1^ (N=140) | | | | | |  | ICGC^2^ (N=61) | | | | | |
| --- | --- | --- | --- | --- | --- | --- | --- | --- | --- | --- | --- | --- | --- |
|  | N (%) | Overall Survival (OS) | |  | Relapse-free Survival (RFS) | |  | N (%) | Overall Survival (OS) | |  | Relapse-free Survival (RFS) | |
|  |  | HR (95%CI)^3^ | *P* |  | HR (95%CI)^3^ | *P* |  |  | HR (95%CI)^3^ | *P* |  | HR (95%CI)^3^ | *P* |
| Age^4^ (Range) | 65 (35, 88) | 1.03 (1-1.05) | 0.026 |  | 1.01 (0.99-1.03) | 0.183 |  | 69 (34, 90) | 1.03 (1-1.07) | 0.064 |  | 1.03 (0.99-1.06) | 0.110 |
| Gender |  |  |  |  |  |  |  |  |  |  |  |  |  |
| Female | 63 (45.0%) | Reference |  |  | Reference |  |  | 27 (44.3%) | Reference |  |  | Reference |  |
| Male | 77 (55.0%) | 0.85 (0.54-1.33) | 0.485 |  | 0.99 (0.66-1.47) | 0.953 |  | 34 (55.7%) | 0.97 (0.52-1.81) | 0.917 |  | 1.08 (0.6-1.96) | 0.800 |
| Race |  |  |  |  |  |  |  |  |  |  |  |  |  |
| White | 122 (87.1%) | Reference |  |  | Reference |  |  | 58 (95.1%) | Reference |  |  | Reference |  |
| Others | 14 (10.0%) | 0.64 (0.29-1.41) | 0.273 |  | 0.73 (0.38-1.42) | 0.357 |  | 3 (4.9%) | --- |  |  | 0.75 (0.18-3.12) | 0.695 |
| Not Reported | 4 (2.9%) |  |  |  |  |  |  |  |  |  |  |  |  |
| Cigarette Smoking |  |  |  |  |  |  |  |  |  |  |  |  |  |
| Never | 49 (35.0%) | Reference |  |  | Reference |  |  | 26 (42.6%) | Reference |  |  |  |  |
| Current | 68 (49.5%) | 1.41 (0.69-2.85) | 0.344 |  | 1.36 (0.71-2.58) | 0.351 |  | 7 (11.5%) | 0.63 (0.21-1.9) | 0.415 |  | 0.64 (0.23-1.74) | 0.38 |
| Former | 23 (16.4%) | 0.81 (0.47-1.39) | 0.440 |  | 0.91 (0.57-1.45) | 0.693 |  | 25 (41.0%) | 0.93 (0.47-1.86) | 0.846 |  | 0.88 (0.45-1.69) | 0.693 |
| Not Reported |  |  |  |  |  |  |  | 3 (4.9%) |  |  |  |  |  |
| Tumor Stage |  |  |  |  |  |  |  |  |  |  |  |  |  |
| Resectable (I/IIA) | 36 (25.7%) | Reference |  |  | Reference |  |  | 24 (39.3%) | Reference |  |  | Reference |  |
| Borderline Resectable (IIB) | 104 (74.3%) | 1.68 (0.96-2.96) | 0.070 |  | 1.36 (0.85-2.17) | 0.197 |  | 37 (60.7%) | 1.81 (0.9-3.64) | 0.097 |  | 1.82 (0.93-3.54) | 0.079 |
| Tumor Grade |  |  |  |  |  |  |  |  |  |  |  |  |  |
| G1 | 19 (13.6%) |  |  |  |  |  |  | 1 (1.6%) |  |  |  |  |  |
| G2 | 78 (55.7%) | Reference |  |  | Reference |  |  | 36 (59.0%) | Reference |  |  | Reference |  |
| G3 | 42 (30.0%) | 1.2 (0.73-1.97) | 0.469 |  | 1.34 (0.87-2.07) | 0.184 |  | 22 (36.1%) | 1.46 (0.77-2.8) | 0.250 |  | 1.34 (0.72-2.49) | 0.349 |
| G4 | 1 (0.7%) |  |  |  |  |  |  | 2 (3.3%) |  |  |  |  |  |
| Chemotherapy |  |  |  |  |  |  |  |  |  |  |  |  |  |
| No | 29 (20.7%) | Reference |  |  | Reference |  |  | --- |  |  |  | --- |  |
| Yes | 103 (73.6%) | 0.27 (0.16-0.46) | 7.50E-7 |  | 0.4 (0.25-0.64) | 1.42E-4 |  | --- |  |  |  | --- |  |
| Not Reported | 8 (5.7%) |  |  |  |  |  |  | --- |  |  |  | --- |  |
| Radiation Therapy |  |  |  |  |  |  |  |  |  |  |  |  |  |
| No | 88 (62.9%) | Reference |  |  | Reference |  |  | --- |  |  |  | --- |  |
| Yes | 39 (27.9%) | 0.41 (0.23-0.73) | 0.002 |  | 0.6 (0.38-0.93) | 0.024 |  | --- |  |  |  | --- |  |
| Not Reported | 13 (9.3%) |  |  |  |  |  |  | --- |  |  |  | --- |  |

^1^ TCGA, The Cancer Genome Atlas.

^2^ ICGC, International Cancer Genome Consortium.

^3^ HR, Hazard ratio calculated by Cox proportional hazards model. If the hazard ratio is >1, it indicates that the group of interest has a shorter survival than the reference group, and if the hazard ratio is <1, it indicates that the group of interest has a longer survival than the reference group.

^4^ For age as a continuous variable, HR per 1 year of age.

**Supplemental Table 2. Association of tumor microbial species with early-stage PDAC OS and RFS in the meta-analysis of TCGA and ICGC cohorts.**

| Species | OS | | | | | | | RFS^a^ | | | | | | |
| --- | --- | --- | --- | --- | --- | --- | --- | --- | --- | --- | --- | --- | --- | --- |
|  | Meta-analyzed estimate^a^ | | | Permutation test^c^ | | Heterogeneity by cohort^d^ | | Meta-analyzed estimate^a^ | | | Permutation test^c^ | | Heterogeneity by cohort^d^ | |
|  | HR (95%CI) | *P*_meta_ | Proportion  (%) | *P*-value | Q-value | *I*^2^ | *P*_het_ | HR (95%CI) | *P*_meta_ | Proportion  (%) | *P*-value | Q-value | *I*^2^ | *P*_het_ |
| *Hymenobacter sp. IS2118* | 0.79 (0.67-0.94) | **7.83E-03** | **91** | 0.002 | 0.016 | 0 | 0.440 | 0.82 (0.71-0.95) | **9.23E-03** | **94** | 0.002 | 0.012 | 0 | 0.936 |
| *Pseudomonas luteola* | 1.48 (1.1-2.01) | **0.011** | **89** | 0.002 | 0.016 | 0 | 0.555 | 1.45 (1.06-1.98) | **0.021** | **76** | 0.002 | 0.012 | 0 | 0.871 |
| *Escherichia coli* | 0.79 (0.66-0.95) | **0.013** | **79** | 0.002 | 0.016 | 0 | 0.605 | 0.76 (0.64-0.9) | **1.29E-03** | **97** | 0.002 | 0.012 | 0 | 0.552 |
| *Afipia broomeae* | 0.82 (0.69-0.96) | **0.015** | **87** | 0.002 | 0.016 | 0 | 0.779 | 0.76 (0.52-1.12) | 0.171 | 7 | 0.002 | 0.012 | 55 | 0.136 |
| *Chelatococcus sambhunathii* | 1.32 (1.05-1.66) | **0.017** | **81** | 0.002 | 0.016 | 0 | 0.799 | 1.18 (0.95-1.46) | 0.133 | 7 | 0.002 | 0.012 | 0 | 0.760 |
| *Shigella flexneri* | 1.05 (1.01-1.09) | **0.019** | **83** | 0.002 | 0.016 | 0 | 0.726 | 1.06 (1.02-1.1) | **2.24E-03** | **100** | 0.002 | 0.012 | 0 | 0.728 |
| *Alcaligenes faecalis* | 1.16 (1.02-1.32) | **0.021** | **67** | 0.002 | 0.016 | 0 | 0.658 | 1.14 (1.01-1.28) | **0.039** | 43 | 0.002 | 0.012 | 0 | 0.619 |
| *Mycobacterium sp. Root265* | 1.15 (1.02-1.29) | **0.023** | **70** | 0.002 | 0.016 | 0 | 0.825 | 1.2 (1.03-1.39) | **0.017** | **65** | 0.002 | 0.012 | 32.9 | 0.222 |
| *Streptococcus infantis* | 1.1 (1.01-1.19) | **0.024** | **71** | 0.002 | 0.016 | 0 | 0.582 | 1.11 (0.97-1.27) | 0.118 | 10 | 0.002 | 0.012 | 54.7 | 0.137 |
| *Acinetobacter lwoffii* | 1.13 (1.01-1.25) | **0.026** | **65** | 0.002 | 0.016 | 0 | 0.686 | 1.11 (0.99-1.25) | 0.083 | 17 | 0.002 | 0.012 | 24.1 | 0.251 |
| *Gardnerella vaginalis* | 1.09 (1.01-1.18) | **0.030** | **64** | 0.002 | 0.016 | 0 | 0.692 | 1.08 (1-1.16) | **0.045** | 49 | 0.002 | 0.012 | 0 | 0.742 |
| *Pseudomonas sp. StFLB209* | 0.7 (0.5-0.98) | **0.036** | 45 | 0.002 | 0.016 | 0 | 0.786 | 0.88 (0.66-1.16) | 0.352 | 0 | 0.002 | 1.000 | 0 | 0.562 |
| *Paucibacter sp. KCTC 42545* | 0.78 (0.61-0.98) | **0.036** | 49 | 0.002 | 0.016 | 0 | 0.422 | 0.93 (0.76-1.14) | 0.479 | 0 | 0.002 | 1.000 | 0 | 0.557 |
| *Azospira oryzae* | 0.79 (0.64-0.99) | **0.038** | 52 | 0.002 | 0.016 | 0 | 0.902 | 0.82 (0.68-0.99) | **0.039** | 40 | 0.002 | 0.012 | 0 | 0.396 |
| *Leifsonia aquatica* | 0.76 (0.58-0.98) | **0.038** | 45 | 0.002 | 0.016 | 0 | 0.662 | 0.78 (0.6-1) | 0.054 | 20 | 0.002 | 0.012 | 6.2 | 0.302 |
| *Pseudomonas fluorescens* | 0.87 (0.77-0.99) | **0.038** | 49 | 0.002 | 0.016 | 0 | 0.889 | 0.87 (0.77-0.98) | **0.022** | 60 | 0.002 | 0.012 | 0 | 0.416 |
| *Helicobacter ailurogastricus* | 1.11 (1.01-1.23) | **0.039** | 39 | 0.002 | 0.016 | 0 | 0.318 | 1.08 (0.99-1.19) | 0.076 | 24 | 0.002 | 0.012 | 0 | 0.512 |
| *Paenisporosarcina sp. TG-14* | 1.34 (1.01-1.76) | **0.040** | 44 | 0.002 | 0.016 | 0 | 0.402 | 1.13 (0.86-1.48) | 0.375 | 2 | 0.002 | 1.000 | 0 | 0.604 |
| *Candidatus Burkholderia crenata* | 1.18 (1-1.39) | **0.049** | 56 | 0.002 | 0.016 | 38.8 | 0.201 | 1.16 (1.05-1.29) | **2.70E-03** | **97** | 0.002 | 0.012 | 0 | 0.483 |
| *Brachybacterium muris* | 0.83 (0.69-1) | **0.049** | 37 | 0.002 | 0.016 | 0 | 0.464 | 0.86 (0.74-1) | 0.052 | 37 | 0.002 | 0.012 | 0 | 0.662 |
| *Hydrogenophaga sp. RAC07* | 0.71 (0.51-1) | 0.051 | 34 | 0.002 | 0.016 | 0 | 0.840 | 0.91 (0.69-1.19) | 0.488 | 0 | 0.002 | 1.000 | 0 | 0.349 |
| *Rhodocyclaceae bacterium Paddy-1* | 0.75 (0.55-1.01) | 0.062 | 19 | 0.002 | 0.016 | 0 | 0.435 | 0.55 (0.16-1.86) | 0.340 | 0 | 0.002 | 1.000 | 82.9 | 0.015 |
| *Gemella haemolysans* | 1.11 (0.99-1.24) | 0.065 | 20 | 0.002 | 0.016 | 0 | 0.410 | 1.07 (0.96-1.18) | 0.212 | 1 | 0.002 | 1.000 | 0 | 0.724 |
| *Sphingobium sp. Leaf26* | 0.83 (0.67-1.01) | 0.069 | 12 | 0.002 | 0.016 | 0 | 0.547 | 0.89 (0.74-1.06) | 0.194 | 1 | 0.002 | 1.000 | 0 | 0.965 |
| *Hymenobacter terrenus* | 1.09 (0.99-1.19) | 0.075 | 17 | 0.004 | 0.031 | 0 | 0.812 | 1.08 (1-1.16) | 0.059 | 34 | 0.004 | 0.012 | 0 | 0.923 |
| *Acidovorax radicis* | 0.76 (0.57-1.03) | 0.076 | 12 | 0.002 | 0.016 | 0 | 0.481 | 0.82 (0.61-1.1) | 0.190 | 4 | 0.002 | 1.000 | 5.2 | 0.304 |
| *Nitratireductor indicus* | 1.11 (0.98-1.26) | 0.086 | 16 | 0.002 | 0.016 | 0 | 0.976 | 1.09 (0.98-1.21) | 0.127 | 3 | 0.002 | 0.012 | 0 | 0.734 |
| *Streptococcus sanguinis* | 0.87 (0.73-1.02) | 0.090 | 11 | 0.002 | 0.016 | 0 | 0.880 | 0.89 (0.77-1.04) | 0.137 | 2 | 0.002 | 1.000 | 0 | 0.532 |
| *Bradyrhizobium sp. LTSP857* | 1.22 (0.97-1.54) | 0.092 | 6 | 1.000 | 1.000 | 1.3 | 0.314 | 1.02 (0.67-1.56) | 0.912 | 0 | 1.000 | 1.000 | 52.9 | 0.145 |
| *Actinomyces oris* | 1.22 (0.97-1.54) | 0.094 | 19 | 0.002 | 0.016 | 55.6 | 0.134 | 1.36 (0.83-2.25) | 0.225 | 0 | 0.002 | 1.000 | 89.9 | 0.002 |
| *Arthrobacter sp. L77* | 1.11 (0.98-1.25) | 0.098 | 6 | 0.004 | 0.031 | 0 | 0.507 | 1.13 (1.01-1.25) | **0.030** | **64** | 0.004 | 0.012 | 0 | 0.721 |
| *Comamonas testosteroni* | 0.85 (0.71-1.03) | 0.100 | 9 | 1.000 | 1.000 | 0 | 0.445 | 0.93 (0.78-1.1) | 0.409 | 0 | 1.000 | 1.000 | 0 | 0.870 |
| *filamentous cyanobacterium ESFC-1* | 1.27 (0.95-1.7) | 0.101 | 19 | 0.012 | 0.087 | 0 | 0.920 | 1.28 (1-1.64) | 0.052 | 32 | 0.012 | 0.012 | 0 | 0.659 |
| *Brevibacterium casei* | 0.94 (0.87-1.01) | 0.102 | 13 | 0.002 | 0.016 | 0 | 0.955 | 0.94 (0.88-1) | 0.061 | 31 | 0.002 | 0.012 | 0 | 0.987 |
| *Afipia birgiae* | 1.17 (0.97-1.42) | 0.104 | 15 | 0.002 | 0.016 | 0 | 0.882 | 1.1 (0.92-1.32) | 0.311 | 2 | 0.002 | 0.527 | 0 | 0.819 |
| *Acinetobacter rudis* | 0.91 (0.81-1.02) | 0.106 | 11 | 0.002 | 0.016 | 0 | 0.955 | 0.92 (0.82-1.02) | 0.123 | 6 | 0.002 | 1.000 | 0 | 0.857 |
| *Tenacibaculum mesophilum* | 1.1 (0.98-1.23) | 0.108 | 3 | 1.000 | 1.000 | 0 | 0.365 | 1.1 (1-1.22) | 0.060 | 16 | 1.000 | 0.012 | 0 | 0.447 |
| *Acinetobacter johnsonii* | 1.08 (0.98-1.19) | 0.119 | 7 | 0.002 | 0.016 | 3.8 | 0.308 | 1.12 (1.03-1.22) | **0.011** | **93** | 0.002 | 0.012 | 0 | 0.753 |
| *Streptococcus parasanguinis* | 1.21 (0.95-1.54) | 0.122 | 5 | 0.002 | 0.016 | 12.4 | 0.285 | 1.21 (0.88-1.64) | 0.237 | 3 | 0.002 | 1.000 | 41.2 | 0.192 |
| *Phycicoccus sp. Soil803* | 1.09 (0.98-1.21) | 0.127 | 4 | 1.000 | 1.000 | 0 | 0.878 | 1.08 (0.99-1.19) | 0.093 | 6 | 1.000 | 0.012 | 0 | 0.636 |
| *Acinetobacter baumannii* | 0.91 (0.81-1.03) | 0.131 | 1 | 0.956 | 1.000 | 0 | 0.616 | 0.96 (0.86-1.07) | 0.470 | 0 | 0.956 | 1.000 | 0 | 0.887 |
| *Pseudomonas alcaligenes* | 0.84 (0.67-1.05) | 0.132 | 4 | 0.222 | 1.000 | 0 | 0.586 | 0.94 (0.77-1.15) | 0.557 | 0 | 0.222 | 1.000 | 0 | 0.646 |
| *Elizabethkingia anophelis* | 1.17 (0.95-1.44) | 0.136 | 6 | 0.014 | 0.100 | 11.6 | 0.287 | 1.24 (1.04-1.47) | **0.014** | **69** | 0.014 | 0.012 | 0 | 0.991 |
| *Herminiimonas arsenicoxydans* | 0.9 (0.78-1.03) | 0.138 | 0 | 1.000 | 1.000 | 0 | 0.920 | 0.91 (0.8-1.03) | 0.151 | 2 | 1.000 | 1.000 | 0 | 0.338 |
| *Streptococcus salivarius* | 1.09 (0.97-1.21) | 0.145 | 9 | 0.457 | 1.000 | 0 | 0.336 | 1.05 (0.94-1.16) | 0.403 | 0 | 0.457 | 1.000 | 0 | 0.406 |
| *Lawsonella clevelandensis* | 1.13 (0.96-1.34) | 0.148 | 5 | 0.094 | 0.616 | 35.4 | 0.214 | 1.17 (0.93-1.46) | 0.179 | 3 | 0.094 | 0.012 | 65 | 0.091 |
| *Atopobium vaginae* | 1.24 (0.93-1.65) | 0.152 | 35 | 0.002 | 0.016 | 24.2 | 0.251 | 1.19 (0.87-1.63) | 0.268 | 20 | 0.002 | 0.012 | 28.7 | 0.236 |
| *Bradyrhizobium sp. AS23.2* | 1.14 (0.95-1.36) | 0.152 | 8 | 0.002 | 0.016 | 0 | 0.589 | 1.11 (0.86-1.42) | 0.430 | 7 | 0.002 | 0.012 | 28.4 | 0.237 |
| *Sphingomonas paucimobilis* | 0.92 (0.81-1.03) | 0.158 | 1 | 0.311 | 1.000 | 0 | 0.320 | 0.95 (0.85-1.06) | 0.378 | 0 | 0.311 | 1.000 | 0 | 0.604 |
| *Richelia intracellularis* | 0.62 (0.32-1.21) | 0.161 | 38 | 0.002 | 0.016 | 34.6 | 0.216 | 0.25 (0.02-3.02) | 0.273 | 0 | 0.002 | 1.000 | 90.6 | 0.001 |
| *Acidovorax ebreus* | 0.8 (0.58-1.1) | 0.164 | 11 | 0.002 | 0.016 | 42.5 | 0.187 | 0.89 (0.77-1.02) | 0.097 | 7 | 0.002 | 0.012 | 0 | 0.379 |
| *Methylobacterium radiotolerans* | 1.16 (0.94-1.43) | 0.167 | 4 | 0.022 | 0.153 | 0 | 0.899 | 1.1 (0.92-1.33) | 0.301 | 1 | 0.022 | 1.000 | 0 | 0.428 |
| *Janthinobacterium sp. CG3* | 1.66 (0.8-3.43) | 0.170 | 3 | 0.992 | 1.000 | 67.5 | 0.080 | 1.61 (0.76-3.39) | 0.211 | 0 | 0.992 | 1.000 | 66.8 | 0.083 |
| *Staphylococcus capitis* | 1.08 (0.97-1.21) | 0.172 | 1 | 0.988 | 1.000 | 0 | 0.463 | 1.02 (0.92-1.13) | 0.714 | 0 | 0.988 | 1.000 | 0 | 0.903 |
| *Serratia liquefaciens* | 1.19 (0.92-1.55) | 0.182 | 0 | 1.000 | 1.000 | 0 | 0.444 | 0.93 (0.63-1.37) | 0.699 | 0 | 1.000 | 1.000 | 29.8 | 0.233 |
| *Alicyclobacillus acidocaldarius* | 1.11 (0.95-1.3) | 0.187 | 3 | 0.363 | 1.000 | 0 | 0.925 | 1.1 (0.73-1.66) | 0.651 | 38 | 0.363 | 0.012 | 34.2 | 0.218 |
| *Variovorax paradoxus* | 0.76 (0.5-1.14) | 0.189 | 3 | 0.146 | 0.887 | 0 | 0.650 | 0.92 (0.65-1.3) | 0.645 | 0 | 0.146 | 1.000 | 0 | 0.382 |
| *Rheinheimera sp. KL1* | 0.84 (0.65-1.09) | 0.191 | 1 | 0.752 | 1.000 | 0 | 0.357 | 0.69 (0.31-1.55) | 0.372 | 0 | 0.752 | 1.000 | 79.3 | 0.028 |
| *Meiothermus silvanus* | 0.89 (0.74-1.06) | 0.196 | 2 | 0.838 | 1.000 | 0 | 0.328 | 0.87 (0.74-1.03) | 0.096 | 15 | 0.838 | 0.012 | 0 | 0.596 |
| *Prevotella melaninogenica* | 1.13 (0.94-1.37) | 0.199 | 4 | 0.010 | 0.074 | 0 | 0.334 | 1.12 (0.93-1.35) | 0.228 | 7 | 0.010 | 0.012 | 8 | 0.297 |
| *Lautropia mirabilis* | 0.83 (0.63-1.11) | 0.205 | 0 | 1.000 | 1.000 | 0 | 0.819 | 0.82 (0.63-1.06) | 0.129 | 8 | 1.000 | 1.000 | 0 | 0.732 |
| *Brevundimonas diminuta* | 1.14 (0.93-1.39) | 0.208 | 5 | 0.946 | 1.000 | 68.6 | 0.075 | 1.13 (0.88-1.45) | 0.347 | 1 | 0.946 | 1.000 | 82.6 | 0.016 |
| *Kocuria palustris* | 0.84 (0.64-1.1) | 0.211 | 3 | 0.359 | 1.000 | 35.6 | 0.213 | 0.87 (0.68-1.11) | 0.250 | 2 | 0.359 | 1.000 | 28.1 | 0.238 |
| *Mycobacterium obuense* | 0.83 (0.61-1.12) | 0.214 | 0 | 1.000 | 1.000 | 0 | 0.574 | 0.86 (0.48-1.53) | 0.598 | 0 | 1.000 | 1.000 | 49.8 | 0.158 |
| *Bifidobacterium aesculapii* | 0.96 (0.9-1.02) | 0.221 | 1 | 1.000 | 1.000 | 0 | 0.659 | 0.95 (0.89-1.02) | 0.168 | 3 | 1.000 | 0.012 | 19.9 | 0.264 |
| *Methylobacterium sp. ARG-1* | 0.9 (0.76-1.07) | 0.226 | 0 | 1.000 | 1.000 | 0 | 0.708 | 0.89 (0.76-1.05) | 0.160 | 4 | 1.000 | 0.012 | 0 | 0.934 |
| *Dysgonomonas sp. HGC4* | 0.95 (0.88-1.03) | 0.229 | 1 | 0.515 | 1.000 | 0 | 0.679 | 0.98 (0.91-1.05) | 0.588 | 0 | 0.515 | 1.000 | 0 | 0.488 |
| *Mesorhizobium sp. UASWS1009* | 0.87 (0.69-1.09) | 0.230 | 6 | 0.006 | 0.046 | 43.4 | 0.184 | 0.87 (0.69-1.09) | 0.234 | 14 | 0.006 | 0.012 | 49.2 | 0.161 |
| *Achromobacter xylosoxidans* | 0.86 (0.67-1.1) | 0.230 | 38 | 0.002 | 0.016 | 22.1 | 0.257 | 0.87 (0.76-1) | 0.055 | 36 | 0.002 | 0.012 | 0 | 0.521 |
| *Erythrobacter sp. JL475* | 1.09 (0.95-1.25) | 0.235 | 1 | 0.250 | 1.000 | 0 | 0.382 | 1.1 (0.93-1.29) | 0.262 | 10 | 0.250 | 0.012 | 18.1 | 0.269 |
| *Rhizobacter sp. Root404* | 0.8 (0.55-1.16) | 0.238 | 0 | 1.000 | 1.000 | 0 | 0.537 | 0.65 (0.29-1.42) | 0.277 | 1 | 1.000 | 0.439 | 62.7 | 0.102 |
| *Acinetobacter junii* | 0.94 (0.84-1.04) | 0.244 | 0 | 1.000 | 1.000 | 0 | 0.586 | 0.97 (0.88-1.06) | 0.519 | 0 | 1.000 | 1.000 | 0 | 0.377 |
| *Bacillus coagulans* | 1.12 (0.93-1.35) | 0.245 | 0 | 1.000 | 1.000 | 0 | 0.479 | 0.69 (0.23-2.05) | 0.502 | 0 | 1.000 | 1.000 | 71.3 | 0.062 |
| *Pseudomonas fragi* | 0.84 (0.63-1.13) | 0.246 | 3 | 0.924 | 1.000 | 0 | 0.921 | 0.86 (0.66-1.11) | 0.247 | 2 | 0.924 | 1.000 | 0 | 0.749 |
| *Nocardia otitidiscaviarum* | 1.1 (0.93-1.3) | 0.247 | 0 | 1.000 | 1.000 | 0 | 0.931 | 1.04 (0.89-1.21) | 0.650 | 0 | 1.000 | 1.000 | 0 | 0.742 |
| *Pantoea rwandensis* | 1.1 (0.94-1.28) | 0.247 | 9 | 0.002 | 0.016 | 25.8 | 0.246 | 1.06 (0.91-1.25) | 0.442 | 3 | 0.002 | 0.067 | 34.3 | 0.217 |
| *Asinibacterium sp. OR53* | 0.88 (0.71-1.09) | 0.252 | 26 | 0.002 | 0.016 | 43.1 | 0.185 | 0.89 (0.72-1.11) | 0.312 | 29 | 0.002 | 0.012 | 45.9 | 0.174 |
| *Pseudomonas entomophila* | 1.15 (0.91-1.44) | 0.253 | 0 | 1.000 | 1.000 | 0 | 0.821 | 1.05 (0.85-1.3) | 0.634 | 0 | 1.000 | 1.000 | 0 | 0.962 |
| *Marmoricola aequoreus* | 1.14 (0.91-1.43) | 0.255 | 1 | 0.966 | 1.000 | 0 | 0.633 | 1.09 (0.88-1.35) | 0.438 | 1 | 0.966 | 1.000 | 0 | 0.817 |
| *Acetivibrio ethanolgignens* | 1.28 (0.84-1.95) | 0.255 | 2 | 0.056 | 0.374 | 0 | 0.425 | 1.46 (0.92-2.31) | 0.108 | 11 | 0.056 | 0.012 | 0 | 0.545 |
| *Pseudomonas aeruginosa* | 0.95 (0.86-1.04) | 0.255 | 0 | 1.000 | 1.000 | 0 | 0.324 | 0.54 (0.17-1.78) | 0.314 | 0 | 1.000 | 1.000 | 84.1 | 0.012 |
| *Bacillus megaterium* | 0.88 (0.7-1.1) | 0.256 | 0 | 1.000 | 1.000 | 0 | 0.944 | 0.76 (0.36-1.58) | 0.463 | 0 | 1.000 | 1.000 | 52.1 | 0.148 |
| *Phycicoccus jejuensis* | 0.92 (0.81-1.06) | 0.257 | 0 | 1.000 | 1.000 | 0 | 0.453 | 0.89 (0.73-1.08) | 0.244 | 1 | 1.000 | 1.000 | 18.7 | 0.267 |
| *Leptotrichia buccalis* | 1.15 (0.9-1.47) | 0.258 | 2 | 0.024 | 0.164 | 0 | 0.370 | 1.21 (0.97-1.52) | 0.085 | 12 | 0.024 | 0.012 | 0 | 0.902 |
| *Anaeromassilibacillus senegalensis* | 0.93 (0.81-1.06) | 0.260 | 0 | 1.000 | 1.000 | 53 | 0.145 | 0.9 (0.81-1.01) | 0.064 | 33 | 1.000 | 0.012 | 31.1 | 0.228 |
| *Nocardioides sp. CF8* | 0.85 (0.64-1.13) | 0.263 | 0 | 1.000 | 1.000 | 0 | 0.404 | 0.87 (0.6-1.26) | 0.471 | 0 | 1.000 | 1.000 | 32.7 | 0.223 |
| *Methylobacterium populi* | 1.15 (0.9-1.47) | 0.266 | 0 | 1.000 | 1.000 | 21.8 | 0.258 | 1.16 (0.87-1.54) | 0.320 | 0 | 1.000 | 1.000 | 44.4 | 0.180 |
| *Caldimonas manganoxidans* | 0.77 (0.48-1.23) | 0.269 | 1 | 1.000 | 1.000 | 72.6 | 0.056 | 0.8 (0.46-1.39) | 0.421 | 0 | 1.000 | 1.000 | 78.7 | 0.030 |
| *[Clostridium] leptum* | 0.92 (0.8-1.06) | 0.275 | 1 | 0.613 | 1.000 | 0 | 0.628 | 0.97 (0.87-1.09) | 0.640 | 0 | 0.613 | 1.000 | 0 | 0.904 |
| *Rhodococcus erythropolis* | 1.13 (0.9-1.42) | 0.279 | 1 | 0.968 | 1.000 | 0 | 0.744 | 1.03 (0.83-1.27) | 0.793 | 0 | 0.968 | 1.000 | 0 | 0.912 |
| *Sphingobium sp. C100* | 0.94 (0.83-1.06) | 0.281 | 0 | 1.000 | 1.000 | 0 | 0.362 | 0.97 (0.78-1.21) | 0.813 | 0 | 1.000 | 1.000 | 69.1 | 0.072 |
| *Staphylococcus warneri* | 1.08 (0.94-1.26) | 0.281 | 1 | 0.774 | 1.000 | 51.5 | 0.151 | 1.08 (0.94-1.24) | 0.252 | 0 | 0.774 | 1.000 | 52.3 | 0.148 |
| *Chryseobacterium indologenes* | 1.11 (0.92-1.35) | 0.281 | 1 | 0.992 | 1.000 | 0 | 0.663 | 1.1 (0.91-1.33) | 0.323 | 0 | 0.992 | 1.000 | 0 | 0.577 |
| *Eggerthellaceae bacterium AT8* | 1.1 (0.92-1.32) | 0.288 | 0 | 1.000 | 1.000 | 0 | 0.346 | 1.09 (0.73-1.64) | 0.663 | 1 | 1.000 | 0.410 | 77.9 | 0.033 |
| *Tolypothrix campylonemoides* | 0.9 (0.74-1.1) | 0.297 | 0 | 1.000 | 1.000 | 0 | 0.339 | 0.85 (0.49-1.45) | 0.543 | 0 | 1.000 | 1.000 | 78.4 | 0.031 |
| *Ralstonia solanacearum* | 1.18 (0.86-1.62) | 0.299 | 1 | 1.000 | 1.000 | 0 | 0.906 | 1.1 (0.82-1.49) | 0.519 | 0 | 1.000 | 1.000 | 0 | 0.930 |
| *Klebsiella pneumoniae* | 1.09 (0.92-1.29) | 0.302 | 2 | 0.982 | 1.000 | 64.3 | 0.094 | 1.07 (0.83-1.38) | 0.587 | 0 | 0.982 | 1.000 | 83.8 | 0.013 |
| *Nocardia brevicatena* | 1.05 (0.96-1.14) | 0.302 | 1 | 0.297 | 1.000 | 0 | 0.839 | 0.96 (0.88-1.05) | 0.354 | 0 | 0.297 | 1.000 | 0 | 0.695 |
| *Thalassobius gelatinovorus* | 1.05 (0.96-1.14) | 0.303 | 0 | 1.000 | 1.000 | 0 | 0.490 | 1.01 (0.92-1.12) | 0.813 | 0 | 1.000 | 1.000 | 26.2 | 0.244 |
| *Cutibacterium acnes* | 0.89 (0.7-1.12) | 0.306 | 0 | 1.000 | 1.000 | 0 | 0.723 | 0.9 (0.71-1.15) | 0.397 | 0 | 1.000 | 1.000 | 19.1 | 0.266 |
| *Leucobacter sp. UCD-THU* | 0.89 (0.71-1.11) | 0.307 | 0 | 1.000 | 1.000 | 47.6 | 0.167 | 0.88 (0.69-1.13) | 0.319 | 0 | 1.000 | 1.000 | 58.2 | 0.122 |
| *Tepidiphilus thermophilus* | 0.43 (0.08-2.23) | 0.312 | 0 | 1.000 | 1.000 | 92.7 | 0.000 | 0.28 (0.02-3.4) | 0.320 | 0 | 1.000 | 1.000 | 95.6 | 0.000 |
| *Bacillus bataviensis* | 1.37 (0.74-2.54) | 0.313 | 0 | 1.000 | 1.000 | 60.9 | 0.110 | 1.11 (0.88-1.41) | 0.362 | 2 | 1.000 | 1.000 | 0 | 0.372 |
| *Acinetobacter towneri* | 1.13 (0.89-1.43) | 0.315 | 0 | 1.000 | 1.000 | 0 | 0.584 | 1.05 (0.85-1.31) | 0.638 | 0 | 1.000 | 1.000 | 0 | 0.383 |
| *Nocardioides sp. Soil797* | 1.05 (0.96-1.15) | 0.317 | 0 | 1.000 | 1.000 | 0 | 0.900 | 1.07 (0.98-1.16) | 0.112 | 10 | 1.000 | 0.012 | 0 | 0.784 |
| *Corynebacterium afermentans* | 0.91 (0.77-1.09) | 0.321 | 0 | 1.000 | 1.000 | 0 | 0.320 | 1.06 (0.92-1.22) | 0.398 | 0 | 1.000 | 1.000 | 0 | 0.731 |
| *Sphingomonas sp. Leaf34* | 1.13 (0.88-1.45) | 0.322 | 0 | 1.000 | 1.000 | 0 | 0.780 | 1.15 (0.87-1.51) | 0.335 | 6 | 1.000 | 0.012 | 30.1 | 0.232 |
| *Mycobacterium nebraskense* | 1.33 (0.75-2.35) | 0.323 | 0 | 1.000 | 1.000 | 64.2 | 0.095 | 1.13 (0.95-1.34) | 0.170 | 1 | 1.000 | 1.000 | 0 | 0.887 |
| *Burkholderia cepacia* | 0.89 (0.71-1.12) | 0.324 | 3 | 0.200 | 1.000 | 42.8 | 0.186 | 0.8 (0.63-1.01) | 0.059 | 43 | 0.200 | 0.012 | 52.7 | 0.146 |
| *Pseudoramibacter alactolyticus* | 0.83 (0.58-1.2) | 0.327 | 0 | 1.000 | 1.000 | 61.2 | 0.108 | 0.84 (0.59-1.18) | 0.313 | 0 | 1.000 | 1.000 | 63.1 | 0.100 |
| *Ralstonia mannitolilytica* | 0.95 (0.84-1.06) | 0.332 | 0 | 1.000 | 1.000 | 0 | 0.908 | 0.97 (0.87-1.07) | 0.518 | 0 | 1.000 | 1.000 | 0 | 0.486 |
| *Agrobacterium fabrum* | 0.9 (0.74-1.11) | 0.333 | 0 | 1.000 | 1.000 | 0 | 0.415 | 0.93 (0.77-1.12) | 0.439 | 0 | 1.000 | 1.000 | 0 | 0.980 |
| *Comamonas aquatica* | 0.86 (0.64-1.16) | 0.334 | 0 | 1.000 | 1.000 | 0 | 0.646 | 0.92 (0.64-1.31) | 0.635 | 0 | 1.000 | 1.000 | 25.6 | 0.246 |
| *Streptococcus oralis* | 0.94 (0.84-1.06) | 0.340 | 0 | 1.000 | 1.000 | 0 | 0.916 | 0.99 (0.9-1.1) | 0.895 | 0 | 1.000 | 1.000 | 0 | 0.682 |
| *Xanthomonas campestris* | 0.94 (0.84-1.06) | 0.342 | 0 | 1.000 | 1.000 | 0 | 0.755 | 0.9 (0.8-1) | 0.060 | 23 | 1.000 | 0.012 | 0 | 0.894 |
| *Sphingomonas sp. Leaf339* | 1.05 (0.95-1.16) | 0.347 | 2 | 0.104 | 0.656 | 0 | 0.507 | 1.03 (0.93-1.13) | 0.590 | 1 | 0.104 | 1.000 | 0 | 0.480 |
| *Exiguobacterium profundum* | 1.06 (0.94-1.19) | 0.349 | 0 | 1.000 | 1.000 | 0 | 0.423 | 1.08 (0.98-1.2) | 0.125 | 5 | 1.000 | 0.012 | 0 | 0.838 |
| *Acidovorax caeni* | 0.75 (0.41-1.37) | 0.355 | 0 | 1.000 | 1.000 | 52.8 | 0.146 | 0.61 (0.2-1.84) | 0.377 | 0 | 1.000 | 1.000 | 77 | 0.037 |
| *Deinococcus geothermalis* | 0.7 (0.33-1.48) | 0.356 | 0 | 1.000 | 1.000 | 52 | 0.149 | 0.38 (0.04-3.6) | 0.397 | 0 | 1.000 | 1.000 | 91 | 0.001 |
| *Hydrogenophaga intermedia* | 0.94 (0.84-1.07) | 0.357 | 0 | 1.000 | 1.000 | 0 | 0.613 | 1.03 (0.94-1.14) | 0.518 | 1 | 1.000 | 1.000 | 0 | 0.864 |
| *Pseudomonas rhizosphaerae* | 1.14 (0.85-1.53) | 0.371 | 0 | 1.000 | 1.000 | 33.4 | 0.220 | 1.13 (0.9-1.42) | 0.305 | 2 | 1.000 | 1.000 | 0 | 0.828 |
| *Catellicoccus marimammalium* | 1.33 (0.71-2.5) | 0.372 | 0 | 1.000 | 1.000 | 0 | 0.947 | 0.91 (0.5-1.65) | 0.744 | 0 | 1.000 | 1.000 | 0 | 0.331 |
| *[Polyangium] brachysporum* | 0.86 (0.61-1.2) | 0.376 | 0 | 1.000 | 1.000 | 0 | 0.978 | 0.78 (0.57-1.08) | 0.130 | 7 | 1.000 | 0.012 | 0 | 0.361 |
| *Shigella dysenteriae* | 1.03 (0.97-1.09) | 0.377 | 0 | 1.000 | 1.000 | 0 | 0.611 | 1.04 (0.99-1.1) | 0.125 | 4 | 1.000 | 0.012 | 0 | 0.618 |
| *Mesorhizobium sp. ORS3324* | 0.97 (0.9-1.04) | 0.379 | 0 | 1.000 | 1.000 | 45.4 | 0.176 | 0.95 (0.9-1) | **0.042** | 31 | 1.000 | 0.012 | 0 | 0.440 |
| *Kocuria polaris* | 0.84 (0.56-1.25) | 0.381 | 0 | 1.000 | 1.000 | 41 | 0.193 | 0.79 (0.42-1.46) | 0.447 | 0 | 1.000 | 1.000 | 71.5 | 0.061 |
| *Stenotrophomonas maltophilia* | 1.09 (0.9-1.32) | 0.385 | 0 | 1.000 | 1.000 | 0 | 0.615 | 1.04 (0.87-1.24) | 0.683 | 0 | 1.000 | 1.000 | 0 | 0.438 |
| *Tropicibacter multivorans* | 0.96 (0.87-1.05) | 0.388 | 0 | 1.000 | 1.000 | 0 | 0.723 | 0.95 (0.88-1.04) | 0.274 | 0 | 1.000 | 1.000 | 0 | 0.988 |
| *beta proteobacterium AAP51* | 0.86 (0.62-1.21) | 0.389 | 1 | 0.856 | 1.000 | 0 | 0.741 | 1.02 (0.75-1.38) | 0.892 | 0 | 0.856 | 1.000 | 0 | 0.413 |
| *Methylobacterium aquaticum* | 0.89 (0.68-1.16) | 0.390 | 0 | 1.000 | 1.000 | 0 | 0.954 | 0.83 (0.65-1.07) | 0.154 | 5 | 1.000 | 0.012 | 0 | 0.988 |
| *Corynebacterium accolens* | 1.08 (0.9-1.3) | 0.390 | 2 | 0.998 | 1.000 | 40.5 | 0.195 | 1.11 (0.88-1.4) | 0.390 | 5 | 0.998 | 0.858 | 66.4 | 0.084 |
| *Leclercia adecarboxylata* | 1.09 (0.9-1.32) | 0.394 | 0 | 1.000 | 1.000 | 0 | 0.464 | 0.95 (0.78-1.17) | 0.646 | 0 | 1.000 | 1.000 | 0 | 0.392 |
| *Nocardioides sp. Soil774* | 0.92 (0.77-1.11) | 0.396 | 0 | 1.000 | 1.000 | 0 | 0.854 | 1.01 (0.86-1.2) | 0.874 | 0 | 1.000 | 1.000 | 0 | 0.380 |
| *Rhizobacter sp. Root29* | 0.73 (0.36-1.51) | 0.397 | 0 | 1.000 | 1.000 | 75 | 0.046 | 0.8 (0.49-1.28) | 0.347 | 0 | 1.000 | 1.000 | 53.9 | 0.141 |
| *Haemophilus sputorum* | 1.11 (0.87-1.41) | 0.397 | 0 | 1.000 | 1.000 | 82.6 | 0.017 | 1.1 (0.98-1.23) | 0.118 | 10 | 1.000 | 0.435 | 39.4 | 0.199 |
| *Asticcacaulis excentricus* | 0.81 (0.49-1.34) | 0.413 | 0 | 1.000 | 1.000 | 63.8 | 0.096 | 0.82 (0.67-1.01) | 0.068 | 22 | 1.000 | 0.012 | 3.4 | 0.309 |
| *Silanimonas lenta* | 0.91 (0.72-1.14) | 0.416 | 0 | 1.000 | 1.000 | 0 | 0.801 | 0.8 (0.48-1.32) | 0.382 | 0 | 1.000 | 1.000 | 45.3 | 0.176 |
| *Pelomonas sp. Root662* | 0.92 (0.74-1.13) | 0.419 | 2 | 0.880 | 1.000 | 44.4 | 0.180 | 1.01 (0.72-1.42) | 0.940 | 0 | 0.880 | 1.000 | 79.6 | 0.027 |
| *Faecalibacterium prausnitzii* | 0.91 (0.72-1.15) | 0.424 | 1 | 0.623 | 1.000 | 0 | 0.795 | 0.99 (0.81-1.2) | 0.888 | 0 | 0.623 | 1.000 | 0 | 0.324 |
| *Massilia sp. Leaf139* | 1.1 (0.87-1.4) | 0.426 | 0 | 1.000 | 1.000 | 0 | 0.460 | 0.95 (0.58-1.56) | 0.849 | 0 | 1.000 | 1.000 | 59 | 0.118 |
| *Desulfocarbo indianensis* | 0.91 (0.72-1.15) | 0.427 | 0 | 1.000 | 1.000 | 0 | 0.852 | 0.56 (0.15-2.09) | 0.388 | 0 | 1.000 | 1.000 | 78.2 | 0.032 |
| *Corynebacterium amycolatum* | 0.96 (0.86-1.07) | 0.429 | 0 | 1.000 | 1.000 | 0 | 0.999 | 1 (0.91-1.09) | 0.941 | 0 | 1.000 | 1.000 | 0 | 0.659 |
| *Lactococcus lactis* | 0.6 (0.17-2.16) | 0.433 | 0 | 1.000 | 1.000 | 87.8 | 0.004 | 0.43 (0.07-2.85) | 0.382 | 0 | 1.000 | 1.000 | 93.2 | 0.000 |
| *Finegoldia magna* | 0.95 (0.85-1.07) | 0.439 | 0 | 1.000 | 1.000 | 0 | 0.458 | 0.98 (0.88-1.09) | 0.723 | 0 | 1.000 | 1.000 | 0 | 0.599 |
| *Cystobacter fuscus* | 1.08 (0.89-1.3) | 0.439 | 0 | 1.000 | 1.000 | 0 | 0.406 | 0.91 (0.66-1.26) | 0.582 | 0 | 1.000 | 1.000 | 32.5 | 0.224 |
| *Aquabacterium parvum* | 0.9 (0.69-1.17) | 0.442 | 0 | 1.000 | 1.000 | 15.1 | 0.278 | 1.05 (0.72-1.55) | 0.791 | 0 | 1.000 | 1.000 | 64.9 | 0.092 |
| *Microlunatus phosphovorus* | 0.9 (0.68-1.18) | 0.444 | 0 | 1.000 | 1.000 | 0 | 0.423 | 0.78 (0.38-1.63) | 0.515 | 0 | 1.000 | 1.000 | 74.6 | 0.047 |
| *Thermoanaerobacterium thermosaccharolyticum* | 1.04 (0.94-1.14) | 0.448 | 0 | 1.000 | 1.000 | 0 | 0.606 | 1.06 (0.8-1.41) | 0.690 | 0 | 1.000 | 1.000 | 88.6 | 0.003 |
| *Corynebacterium pseudodiphtheriticum* | 1.13 (0.83-1.54) | 0.452 | 0 | 1.000 | 1.000 | 60.4 | 0.112 | 1.14 (0.95-1.37) | 0.169 | 1 | 1.000 | 1.000 | 7.6 | 0.298 |
| *Azohydromonas australica* | 0.89 (0.65-1.21) | 0.454 | 0 | 1.000 | 1.000 | 0 | 0.900 | 0.8 (0.59-1.08) | 0.150 | 4 | 1.000 | 1.000 | 0 | 0.362 |
| *Rhizobium sp. Root651* | 0.94 (0.8-1.11) | 0.464 | 0 | 1.000 | 1.000 | 0 | 0.905 | 1.01 (0.87-1.17) | 0.935 | 0 | 1.000 | 1.000 | 0 | 0.658 |
| *Janthinobacterium sp. HH01* | 1.06 (0.91-1.23) | 0.469 | 0 | 1.000 | 1.000 | 0 | 0.916 | 1.05 (0.92-1.2) | 0.457 | 0 | 1.000 | 1.000 | 0 | 0.700 |
| *Xenophilus azovorans* | 1.1 (0.85-1.43) | 0.470 | 2 | 0.617 | 1.000 | 0 | 0.609 | 1.05 (0.83-1.33) | 0.685 | 0 | 0.617 | 1.000 | 0 | 0.549 |
| *Pseudomonas protegens* | 1.06 (0.9-1.26) | 0.475 | 0 | 1.000 | 1.000 | 0 | 0.958 | 1.05 (0.91-1.22) | 0.510 | 0 | 1.000 | 1.000 | 0 | 0.737 |
| *Aerococcus viridans* | 0.78 (0.39-1.56) | 0.480 | 0 | 1.000 | 1.000 | 76.7 | 0.038 | 0.67 (0.25-1.75) | 0.411 | 0 | 1.000 | 1.000 | 84.4 | 0.011 |
| *Pseudoxanthomonas suwonensis* | 0.9 (0.68-1.2) | 0.482 | 0 | 1.000 | 1.000 | 32.1 | 0.225 | 0.93 (0.78-1.11) | 0.423 | 0 | 1.000 | 1.000 | 0 | 0.831 |
| *Lactobacillus iners* | 0.97 (0.89-1.06) | 0.486 | 0 | 1.000 | 1.000 | 0 | 0.401 | 0.91 (0.78-1.08) | 0.281 | 0 | 1.000 | 1.000 | 45.5 | 0.175 |
| *Corynebacterium aurimucosum* | 0.98 (0.92-1.04) | 0.486 | 1 | 0.335 | 1.000 | 0 | 0.900 | 1 (0.95-1.06) | 0.926 | 0 | 0.335 | 1.000 | 0 | 0.509 |
| *Ornithinimicrobium pekingense* | 1.09 (0.86-1.37) | 0.488 | 0 | 1.000 | 1.000 | 0 | 0.374 | 0.95 (0.53-1.72) | 0.867 | 0 | 1.000 | 1.000 | 49.8 | 0.158 |
| *Olsenella sp. SIT9* | 1.06 (0.9-1.23) | 0.489 | 0 | 1.000 | 1.000 | 0 | 0.463 | 0.83 (0.39-1.75) | 0.621 | 0 | 1.000 | 1.000 | 53 | 0.145 |
| *Sphingomonas melonis* | 0.96 (0.84-1.09) | 0.491 | 0 | 1.000 | 1.000 | 0 | 0.816 | 0.99 (0.88-1.11) | 0.819 | 0 | 1.000 | 1.000 | 0 | 0.743 |
| *Staphylococcus cohnii* | 0.96 (0.84-1.09) | 0.491 | 0 | 1.000 | 1.000 | 0 | 0.387 | 0.92 (0.82-1.04) | 0.184 | 0 | 1.000 | 1.000 | 0 | 0.434 |
| *Caldicellulosiruptor bescii* | 1.16 (0.76-1.78) | 0.493 | 0 | 1.000 | 1.000 | 68.5 | 0.075 | 1.02 (0.91-1.14) | 0.754 | 0 | 1.000 | 1.000 | 0 | 0.382 |
| *Methylobacterium sp. 77* | 0.9 (0.66-1.23) | 0.498 | 0 | 1.000 | 1.000 | 0 | 0.450 | 1.01 (0.76-1.34) | 0.942 | 0 | 1.000 | 1.000 | 0 | 0.686 |
| *Massilia sp. WG5* | 1.06 (0.9-1.24) | 0.499 | 0 | 1.000 | 1.000 | 12.3 | 0.286 | 0.98 (0.82-1.17) | 0.842 | 0 | 1.000 | 1.000 | 28.6 | 0.237 |
| *Pantoea agglomerans* | 1.08 (0.86-1.35) | 0.501 | 0 | 1.000 | 1.000 | 0 | 0.432 | 1.05 (0.85-1.29) | 0.641 | 0 | 1.000 | 1.000 | 0 | 0.327 |
| *Microbacterium paraoxydans* | 1.1 (0.83-1.46) | 0.502 | 0 | 1.000 | 1.000 | 0 | 0.617 | 1.03 (0.77-1.37) | 0.849 | 0 | 1.000 | 1.000 | 0 | 0.662 |
| *Sphingomonas sp. Leaf33* | 1.11 (0.81-1.53) | 0.509 | 2 | 0.096 | 0.617 | 61.1 | 0.109 | 1.15 (0.95-1.39) | 0.147 | 11 | 0.096 | 0.012 | 12.9 | 0.284 |
| *Blautia obeum* | 1.09 (0.84-1.41) | 0.515 | 0 | 1.000 | 1.000 | 70.1 | 0.068 | 1.04 (0.91-1.18) | 0.561 | 0 | 1.000 | 1.000 | 13.2 | 0.283 |
| *Bradyrhizobium elkanii* | 1.06 (0.89-1.27) | 0.517 | 0 | 1.000 | 1.000 | 16.8 | 0.273 | 1.02 (0.85-1.22) | 0.866 | 0 | 1.000 | 1.000 | 24.7 | 0.249 |
| *Pseudomonas alkylphenolica* | 0.95 (0.8-1.12) | 0.522 | 0 | 1.000 | 1.000 | 0 | 0.863 | 0.97 (0.84-1.12) | 0.664 | 0 | 1.000 | 1.000 | 0 | 0.727 |
| *Acinetobacter ursingii* | 1.03 (0.93-1.14) | 0.536 | 0 | 1.000 | 1.000 | 0 | 0.651 | 1.03 (0.94-1.13) | 0.464 | 0 | 1.000 | 1.000 | 0 | 0.491 |
| *Corynebacterium singulare* | 0.97 (0.9-1.06) | 0.539 | 0 | 1.000 | 1.000 | 4.8 | 0.306 | 1 (0.93-1.07) | 0.993 | 0 | 1.000 | 1.000 | 0 | 0.540 |
| *Janibacter hoylei* | 1.03 (0.94-1.14) | 0.540 | 0 | 1.000 | 1.000 | 0 | 0.871 | 1.03 (0.94-1.13) | 0.569 | 0 | 1.000 | 1.000 | 0 | 0.610 |
| *Cupriavidus taiwanensis* | 1.15 (0.73-1.82) | 0.544 | 1 | 0.996 | 1.000 | 45.5 | 0.176 | 0.93 (0.53-1.61) | 0.785 | 0 | 0.996 | 1.000 | 57.5 | 0.125 |
| *Mycobacterium aurum* | 1.02 (0.95-1.11) | 0.552 | 0 | 1.000 | 1.000 | 0 | 0.322 | 1.02 (0.95-1.1) | 0.582 | 0 | 1.000 | 1.000 | 0 | 0.550 |
| *Rothia mucilaginosa* | 0.94 (0.76-1.16) | 0.553 | 0 | 1.000 | 1.000 | 59.3 | 0.117 | 0.99 (0.89-1.1) | 0.851 | 0 | 1.000 | 1.000 | 0 | 0.554 |
| *Pseudomonas xanthomarina* | 1.08 (0.83-1.4) | 0.555 | 0 | 1.000 | 1.000 | 0 | 0.768 | 0.99 (0.78-1.27) | 0.959 | 0 | 1.000 | 1.000 | 0 | 0.864 |
| *Granulicatella adiacens* | 1.06 (0.87-1.3) | 0.557 | 0 | 1.000 | 1.000 | 0 | 0.834 | 1.03 (0.85-1.25) | 0.743 | 0 | 1.000 | 1.000 | 0 | 0.436 |
| *Sphingomonas sp. Leaf17* | 0.93 (0.72-1.2) | 0.559 | 0 | 1.000 | 1.000 | 0 | 0.649 | 1.02 (0.82-1.27) | 0.873 | 0 | 1.000 | 1.000 | 0.3 | 0.317 |
| *Mesorhizobium plurifarium* | 1.02 (0.96-1.07) | 0.560 | 0 | 1.000 | 1.000 | 0 | 0.784 | 1.01 (0.97-1.06) | 0.630 | 0 | 1.000 | 1.000 | 0 | 0.494 |
| *Neisseria mucosa* | 1.06 (0.88-1.27) | 0.561 | 0 | 1.000 | 1.000 | 0 | 0.679 | 1.05 (0.88-1.25) | 0.576 | 0 | 1.000 | 1.000 | 0 | 0.976 |
| *Actinomyces massiliensis* | 1.04 (0.9-1.2) | 0.561 | 0 | 1.000 | 1.000 | 0 | 0.773 | 1.06 (0.93-1.21) | 0.398 | 0 | 1.000 | 1.000 | 0 | 0.512 |
| *Pseudomonas lutea* | 1.06 (0.86-1.32) | 0.562 | 0 | 1.000 | 1.000 | 9.9 | 0.292 | 1.05 (0.88-1.26) | 0.598 | 0 | 1.000 | 1.000 | 0 | 0.585 |
| *Streptococcus pneumoniae* | 0.88 (0.58-1.35) | 0.562 | 0 | 1.000 | 1.000 | 68.8 | 0.073 | 0.93 (0.67-1.28) | 0.641 | 0 | 1.000 | 1.000 | 61.1 | 0.109 |
| *Aquincola tertiaricarbonis* | 1.08 (0.82-1.43) | 0.568 | 1 | 0.972 | 1.000 | 0 | 0.354 | 1.16 (0.77-1.75) | 0.488 | 0 | 0.972 | 1.000 | 54.8 | 0.137 |
| *Bacillus thuringiensis* | 1.23 (0.6-2.49) | 0.575 | 0 | 1.000 | 1.000 | 70.6 | 0.065 | 0.99 (0.57-1.73) | 0.965 | 0 | 1.000 | 1.000 | 52.1 | 0.148 |
| *Tyzzerella nexilis* | 1.08 (0.82-1.44) | 0.576 | 0 | 1.000 | 1.000 | 77.4 | 0.035 | 1.13 (0.74-1.73) | 0.571 | 0 | 1.000 | 1.000 | 89.3 | 0.002 |
| *Mycobacterium mucogenicum* | 1.08 (0.83-1.41) | 0.579 | 0 | 1.000 | 1.000 | 0 | 0.725 | 0.99 (0.76-1.3) | 0.962 | 0 | 1.000 | 1.000 | 0 | 0.397 |
| *Paenibacillus sp. OSY-SE* | 0.96 (0.82-1.12) | 0.580 | 0 | 1.000 | 1.000 | 69.5 | 0.070 | 0.98 (0.88-1.08) | 0.638 | 0 | 1.000 | 1.000 | 37.2 | 0.207 |
| *Flavobacterium frigoris* | 1.04 (0.92-1.17) | 0.580 | 0 | 1.000 | 1.000 | 0 | 0.441 | 1.03 (0.91-1.17) | 0.634 | 0 | 1.000 | 1.000 | 22.8 | 0.255 |
| *Staphylococcus epidermidis* | 1.02 (0.94-1.11) | 0.585 | 0 | 1.000 | 1.000 | 0 | 0.788 | 1.02 (0.95-1.1) | 0.611 | 0 | 1.000 | 1.000 | 10.9 | 0.289 |
| *Pseudomonas koreensis* | 0.96 (0.84-1.1) | 0.589 | 0 | 1.000 | 1.000 | 0 | 0.670 | 0.95 (0.84-1.07) | 0.404 | 0 | 1.000 | 1.000 | 0 | 0.717 |
| *Ochrobactrum anthropi* | 0.97 (0.85-1.1) | 0.594 | 0 | 1.000 | 1.000 | 0 | 0.527 | 1.03 (0.91-1.15) | 0.658 | 0 | 1.000 | 1.000 | 0 | 0.422 |
| *[Propionibacterium] humerusii* | 1.05 (0.89-1.23) | 0.595 | 0 | 1.000 | 1.000 | 0 | 0.680 | 1.01 (0.86-1.18) | 0.921 | 0 | 1.000 | 1.000 | 11.4 | 0.288 |
| *Micrococcus luteus* | 1.03 (0.91-1.17) | 0.597 | 0 | 1.000 | 1.000 | 78.1 | 0.033 | 1.07 (0.91-1.26) | 0.425 | 0 | 1.000 | 1.000 | 88.4 | 0.003 |
| *Anaerosalibacter massiliensis* | 0.98 (0.92-1.05) | 0.600 | 0 | 1.000 | 1.000 | 0 | 0.358 | 1.01 (0.95-1.07) | 0.815 | 0 | 1.000 | 1.000 | 0 | 0.971 |
| *Labrenzia alba* | 0.94 (0.76-1.17) | 0.600 | 1 | 0.118 | 0.731 | 0 | 0.725 | 0.98 (0.7-1.36) | 0.892 | 0 | 0.118 | 1.000 | 52.7 | 0.146 |
| *Pseudomonas fulva* | 0.93 (0.72-1.22) | 0.610 | 0 | 1.000 | 1.000 | 0 | 0.387 | 1.04 (0.67-1.59) | 0.873 | 0 | 1.000 | 1.000 | 59.9 | 0.114 |
| *Sediminibacterium salmoneum* | 0.95 (0.79-1.15) | 0.610 | 0 | 1.000 | 1.000 | 0 | 0.846 | 0.89 (0.74-1.07) | 0.214 | 1 | 1.000 | 0.057 | 0 | 0.848 |
| *Leifsonia sp. Leaf336* | 1.06 (0.85-1.33) | 0.610 | 0 | 1.000 | 1.000 | 0 | 0.941 | 1.03 (0.82-1.29) | 0.789 | 0 | 1.000 | 1.000 | 11.4 | 0.288 |
| *Corynebacterium minutissimum* | 0.96 (0.81-1.13) | 0.613 | 0 | 1.000 | 1.000 | 0 | 0.833 | 1 (0.87-1.15) | 0.971 | 0 | 1.000 | 1.000 | 0 | 0.942 |
| *Phenylobacterium sp. Root1290* | 1.11 (0.73-1.68) | 0.620 | 0 | 1.000 | 1.000 | 40.7 | 0.194 | 1.07 (0.73-1.55) | 0.735 | 0 | 1.000 | 1.000 | 20.9 | 0.261 |
| *Alkanindiges illinoisensis* | 0.95 (0.79-1.15) | 0.627 | 0 | 1.000 | 1.000 | 7.4 | 0.299 | 0.94 (0.79-1.12) | 0.484 | 0 | 1.000 | 1.000 | 0 | 0.725 |
| *Paracoccus sp. 228* | 1.03 (0.91-1.16) | 0.628 | 0 | 1.000 | 1.000 | 0 | 0.447 | 1.05 (0.93-1.18) | 0.416 | 0 | 1.000 | 1.000 | 0 | 0.612 |
| *Massilia timonae* | 1.04 (0.89-1.21) | 0.629 | 0 | 1.000 | 1.000 | 0 | 0.916 | 1.03 (0.9-1.19) | 0.672 | 0 | 1.000 | 1.000 | 0 | 0.678 |
| *Lamprocystis purpurea* | 1.1 (0.75-1.6) | 0.636 | 0 | 1.000 | 1.000 | 0 | 0.868 | 1.04 (0.73-1.48) | 0.831 | 0 | 1.000 | 1.000 | 0 | 0.555 |
| *Planococcus sp. CAU13* | 1.09 (0.75-1.59) | 0.645 | 0 | 1.000 | 1.000 | 0 | 0.382 | 0.87 (0.58-1.31) | 0.502 | 0 | 1.000 | 1.000 | 0 | 0.385 |
| *Ideonella sp. B508-1* | 0.9 (0.56-1.43) | 0.646 | 0 | 1.000 | 1.000 | 55.2 | 0.135 | 0.98 (0.57-1.67) | 0.937 | 0 | 1.000 | 1.000 | 69 | 0.073 |
| *Klebsiella oxytoca* | 0.96 (0.82-1.13) | 0.649 | 0 | 1.000 | 1.000 | 0 | 0.945 | 0.92 (0.79-1.07) | 0.271 | 0 | 1.000 | 1.000 | 0 | 0.423 |
| *Methylobacterium sp. Leaf89* | 1.08 (0.77-1.52) | 0.649 | 0 | 1.000 | 1.000 | 0 | 0.597 | 0.99 (0.59-1.65) | 0.958 | 0 | 1.000 | 1.000 | 52.8 | 0.145 |
| *Megamonas funiformis* | 0.96 (0.83-1.13) | 0.653 | 0 | 1.000 | 1.000 | 0 | 0.977 | 0.97 (0.84-1.12) | 0.690 | 0 | 1.000 | 1.000 | 0 | 0.762 |
| *Rhodococcus fascians* | 0.94 (0.73-1.21) | 0.653 | 0 | 1.000 | 1.000 | 0 | 0.614 | 1.04 (0.83-1.3) | 0.755 | 0 | 1.000 | 1.000 | 0 | 0.887 |
| *Roseovarius tolerans* | 1.1 (0.73-1.64) | 0.656 | 0 | 1.000 | 1.000 | 81.6 | 0.020 | 1.21 (0.77-1.89) | 0.413 | 0 | 1.000 | 1.000 | 86.9 | 0.006 |
| *Enterobacter sp. 638* | 1.06 (0.82-1.38) | 0.657 | 0 | 1.000 | 1.000 | 0 | 0.763 | 0.93 (0.73-1.19) | 0.556 | 0 | 1.000 | 1.000 | 0 | 0.443 |
| *Nocardia paucivorans* | 1.03 (0.9-1.18) | 0.658 | 0 | 1.000 | 1.000 | 0 | 0.624 | 0.99 (0.88-1.12) | 0.888 | 0 | 1.000 | 1.000 | 0 | 0.996 |
| *Methylobacterium sp. Leaf456* | 1.12 (0.66-1.91) | 0.670 | 0 | 1.000 | 1.000 | 61.7 | 0.106 | 0.96 (0.48-1.9) | 0.904 | 0 | 1.000 | 1.000 | 74.6 | 0.047 |
| *Acidovorax sp. RAC01* | 0.98 (0.87-1.09) | 0.672 | 0 | 1.000 | 1.000 | 0 | 0.495 | 0.98 (0.89-1.08) | 0.689 | 0 | 1.000 | 1.000 | 0 | 0.818 |
| *Brachybacterium paraconglomeratum* | 0.95 (0.74-1.22) | 0.675 | 0 | 1.000 | 1.000 | 52 | 0.149 | 0.97 (0.85-1.11) | 0.688 | 0 | 1.000 | 1.000 | 0 | 0.395 |
| *Sphingomonas sp. Leaf343* | 1.06 (0.79-1.44) | 0.686 | 0 | 1.000 | 1.000 | 0 | 0.524 | 1.11 (0.82-1.5) | 0.490 | 2 | 1.000 | 1.000 | 0 | 0.469 |
| *Helicobacter heilmannii* | 0.98 (0.88-1.09) | 0.689 | 0 | 1.000 | 1.000 | 0 | 0.523 | 0.97 (0.87-1.07) | 0.493 | 0 | 1.000 | 1.000 | 5.6 | 0.303 |
| *Paracoccus yeei* | 1.09 (0.71-1.67) | 0.690 | 6 | 1.000 | 1.000 | 75.5 | 0.043 | 1.04 (0.87-1.23) | 0.678 | 3 | 1.000 | 1.000 | 0 | 0.346 |
| *Anaerococcus prevotii* | 1.03 (0.9-1.17) | 0.690 | 0 | 1.000 | 1.000 | 0 | 0.515 | 1.02 (0.91-1.16) | 0.720 | 0 | 1.000 | 1.000 | 0 | 0.573 |
| *Cellulomonas sp. Root930* | 1.02 (0.92-1.13) | 0.692 | 0 | 1.000 | 1.000 | 0 | 0.933 | 1.05 (0.96-1.15) | 0.303 | 0 | 1.000 | 1.000 | 0 | 0.790 |
| *Pseudomonas oryzihabitans* | 1.04 (0.85-1.28) | 0.698 | 0 | 1.000 | 1.000 | 0 | 0.565 | 1.08 (0.89-1.3) | 0.427 | 0 | 1.000 | 1.000 | 0 | 0.999 |
| *Streptococcus anginosus* | 0.96 (0.78-1.18) | 0.700 | 0 | 1.000 | 1.000 | 23.5 | 0.253 | 0.91 (0.65-1.26) | 0.557 | 0 | 1.000 | 1.000 | 63.2 | 0.099 |
| *Anaerococcus provenciensis* | 1.07 (0.76-1.5) | 0.702 | 0 | 1.000 | 1.000 | 45.7 | 0.175 | 1.07 (0.88-1.31) | 0.478 | 0 | 1.000 | 1.000 | 0 | 0.503 |
| *Ralstonia pickettii* | 0.98 (0.86-1.11) | 0.708 | 0 | 1.000 | 1.000 | 0 | 0.722 | 0.86 (0.62-1.2) | 0.380 | 0 | 1.000 | 1.000 | 60.4 | 0.112 |
| *Massilia putida* | 0.95 (0.72-1.26) | 0.712 | 0 | 1.000 | 1.000 | 26.6 | 0.243 | 0.88 (0.66-1.17) | 0.366 | 0 | 1.000 | 1.000 | 34.9 | 0.215 |
| *Streptococcus mitis* | 1.02 (0.94-1.1) | 0.713 | 0 | 1.000 | 1.000 | 0 | 0.919 | 1.01 (0.91-1.12) | 0.879 | 0 | 1.000 | 1.000 | 31.1 | 0.228 |
| *Campylobacter mucosalis* | 1.02 (0.93-1.11) | 0.715 | 0 | 1.000 | 1.000 | 34.3 | 0.217 | 1.02 (0.97-1.07) | 0.521 | 0 | 1.000 | 1.000 | 0 | 0.518 |
| *Cronobacter sakazakii* | 0.96 (0.76-1.2) | 0.719 | 0 | 1.000 | 1.000 | 0 | 0.805 | 0.94 (0.71-1.24) | 0.665 | 0 | 1.000 | 1.000 | 25.4 | 0.247 |
| *Dolosigranulum pigrum* | 1.03 (0.89-1.17) | 0.721 | 0 | 1.000 | 1.000 | 0 | 0.436 | 1.06 (0.94-1.19) | 0.365 | 0 | 1.000 | 1.000 | 0 | 0.535 |
| *Veillonella parvula* | 1.03 (0.88-1.21) | 0.721 | 0 | 1.000 | 1.000 | 0 | 0.529 | 1.03 (0.89-1.19) | 0.709 | 0 | 1.000 | 1.000 | 0 | 0.473 |
| *Massilia sp. 9096* | 1.03 (0.87-1.23) | 0.722 | 0 | 1.000 | 1.000 | 6.4 | 0.301 | 1.01 (0.81-1.26) | 0.956 | 0 | 1.000 | 1.000 | 43 | 0.185 |
| *Dietzia sp. UCD-THP* | 1.03 (0.87-1.21) | 0.724 | 0 | 1.000 | 1.000 | 46.5 | 0.172 | 1.01 (0.92-1.11) | 0.831 | 0 | 1.000 | 1.000 | 0 | 0.504 |
| *Anoxybacillus gonensis* | 0.93 (0.63-1.38) | 0.724 | 0 | 1.000 | 1.000 | 65 | 0.091 | 0.96 (0.73-1.27) | 0.791 | 0 | 1.000 | 1.000 | 50.7 | 0.154 |
| *Sphingobium baderi* | 1.03 (0.87-1.21) | 0.727 | 0 | 1.000 | 1.000 | 77.6 | 0.035 | 1.04 (0.83-1.3) | 0.750 | 0 | 1.000 | 1.000 | 89.8 | 0.002 |
| *Brevundimonas sp. DS20* | 1.06 (0.74-1.52) | 0.734 | 0 | 1.000 | 1.000 | 0 | 0.645 | 1.05 (0.74-1.48) | 0.781 | 0 | 1.000 | 1.000 | 0 | 0.577 |
| *Brevundimonas nasdae* | 1.02 (0.91-1.15) | 0.736 | 1 | 0.964 | 1.000 | 0 | 0.451 | 1.03 (0.93-1.15) | 0.532 | 0 | 0.964 | 1.000 | 0 | 0.657 |
| *Propionispora sp. 2/2-37* | 1.03 (0.85-1.25) | 0.738 | 0 | 1.000 | 1.000 | 79.6 | 0.027 | 1 (0.86-1.18) | 0.959 | 0 | 1.000 | 1.000 | 76.4 | 0.040 |
| *Corynebacterium kroppenstedtii* | 0.97 (0.79-1.19) | 0.738 | 0 | 1.000 | 1.000 | 0 | 0.974 | 1.02 (0.84-1.22) | 0.867 | 0 | 1.000 | 1.000 | 0 | 0.477 |
| *Cupriavidus gilardii* | 0.98 (0.87-1.1) | 0.739 | 0 | 1.000 | 1.000 | 0 | 0.771 | 0.88 (0.56-1.38) | 0.571 | 0 | 1.000 | 1.000 | 34.6 | 0.216 |
| *Turicella otitidis* | 1.05 (0.78-1.42) | 0.743 | 0 | 1.000 | 1.000 | 36.2 | 0.211 | 1.06 (0.86-1.31) | 0.593 | 0 | 1.000 | 1.000 | 0 | 0.858 |
| *Fusobacterium nucleatum* | 0.97 (0.81-1.17) | 0.746 | 0 | 1.000 | 1.000 | 0 | 0.733 | 0.91 (0.76-1.09) | 0.285 | 0 | 1.000 | 1.000 | 0 | 0.367 |
| *Cupriavidus metallidurans* | 1.02 (0.88-1.19) | 0.754 | 0 | 1.000 | 1.000 | 0 | 0.515 | 0.99 (0.86-1.13) | 0.856 | 0 | 1.000 | 1.000 | 0 | 0.983 |
| *Acinetobacter pittii* | 0.9 (0.46-1.77) | 0.759 | 0 | 1.000 | 1.000 | 85 | 0.010 | 0.8 (0.35-1.86) | 0.610 | 0 | 1.000 | 1.000 | 89.2 | 0.002 |
| *Mesorhizobium sp. NBIMC P2-C3* | 0.96 (0.72-1.28) | 0.760 | 0 | 1.000 | 1.000 | 24.4 | 0.250 | 0.91 (0.63-1.31) | 0.596 | 0 | 1.000 | 1.000 | 35.8 | 0.212 |
| *Thiomonas bhubaneswarensis* | 0.98 (0.89-1.09) | 0.769 | 0 | 1.000 | 1.000 | 0 | 0.694 | 0.96 (0.88-1.05) | 0.348 | 0 | 1.000 | 1.000 | 0 | 0.791 |
| *Caulobacter vibrioides* | 0.92 (0.53-1.59) | 0.772 | 0 | 1.000 | 1.000 | 20.1 | 0.263 | 1.09 (0.71-1.67) | 0.704 | 0 | 1.000 | 1.000 | 0 | 0.912 |
| *Cutibacterium granulosum* | 0.98 (0.83-1.15) | 0.774 | 0 | 1.000 | 1.000 | 0 | 0.572 | 1.02 (0.88-1.19) | 0.786 | 0 | 1.000 | 1.000 | 6.7 | 0.301 |
| *Pseudomonas stutzeri* | 1.01 (0.92-1.12) | 0.776 | 0 | 1.000 | 1.000 | 0 | 0.815 | 1.06 (0.96-1.17) | 0.230 | 2 | 1.000 | 1.000 | 3.4 | 0.309 |
| *Phaeobacter sp. CECT 7735* | 1.03 (0.84-1.27) | 0.780 | 0 | 1.000 | 1.000 | 0 | 0.585 | 0.95 (0.78-1.16) | 0.625 | 0 | 1.000 | 1.000 | 0 | 0.442 |
| *Jannaschia rubra* | 1.06 (0.71-1.56) | 0.783 | 0 | 1.000 | 1.000 | 55.6 | 0.133 | 0.86 (0.66-1.12) | 0.274 | 6 | 1.000 | 0.012 | 29.1 | 0.235 |
| *Sphingomonas sp. Root241* | 0.96 (0.71-1.29) | 0.789 | 0 | 1.000 | 1.000 | 0 | 0.633 | 0.96 (0.73-1.25) | 0.738 | 0 | 1.000 | 1.000 | 0 | 0.549 |
| *Rothia dentocariosa* | 1.01 (0.93-1.1) | 0.795 | 0 | 1.000 | 1.000 | 0 | 0.993 | 1.04 (0.96-1.12) | 0.366 | 0 | 1.000 | 1.000 | 0 | 0.597 |
| *Ideonella sakaiensis* | 1.03 (0.83-1.27) | 0.795 | 0 | 1.000 | 1.000 | 0 | 0.725 | 1.01 (0.84-1.22) | 0.906 | 0 | 1.000 | 1.000 | 0 | 0.935 |
| *Sphingomonas taxi* | 1.03 (0.84-1.26) | 0.796 | 0 | 1.000 | 1.000 | 0 | 0.758 | 1.01 (0.84-1.22) | 0.928 | 0 | 1.000 | 1.000 | 0 | 0.505 |
| *Lactobacillus paracasei* | 0.98 (0.84-1.14) | 0.799 | 0 | 1.000 | 1.000 | 0 | 0.705 | 0.88 (0.6-1.3) | 0.528 | 0 | 1.000 | 1.000 | 47.8 | 0.166 |
| *Staphylococcus saprophyticus* | 1.02 (0.86-1.22) | 0.802 | 0 | 1.000 | 1.000 | 0 | 0.546 | 0.88 (0.74-1.04) | 0.134 | 2 | 1.000 | 0.164 | 0 | 0.410 |
| *Thalassobius sp. CECT 5113* | 1.02 (0.85-1.24) | 0.804 | 0 | 1.000 | 1.000 | 0 | 0.818 | 1.03 (0.86-1.23) | 0.784 | 0 | 1.000 | 1.000 | 0 | 0.818 |
| *Staphylococcus hominis* | 1.01 (0.92-1.11) | 0.805 | 0 | 1.000 | 1.000 | 4.1 | 0.307 | 1.02 (0.95-1.09) | 0.567 | 0 | 1.000 | 1.000 | 0 | 0.329 |
| *Alicycliphilus denitrificans* | 1.08 (0.57-2.07) | 0.807 | 1 | 1.000 | 1.000 | 66.3 | 0.085 | 0.89 (0.77-1.03) | 0.104 | 9 | 1.000 | 1.000 | 0 | 0.543 |
| *Sphingomonas wittichii* | 1.04 (0.75-1.46) | 0.808 | 0 | 1.000 | 1.000 | 0 | 0.408 | 0.9 (0.64-1.25) | 0.530 | 0 | 1.000 | 1.000 | 0 | 0.471 |
| *Cutibacterium avidum* | 1.07 (0.63-1.82) | 0.811 | 0 | 1.000 | 1.000 | 72 | 0.059 | 0.98 (0.46-2.08) | 0.950 | 0 | 1.000 | 1.000 | 84.6 | 0.011 |
| *Chryseobacterium vrystaatense* | 0.96 (0.67-1.37) | 0.812 | 0 | 1.000 | 1.000 | 0 | 0.805 | 0.95 (0.69-1.32) | 0.756 | 0 | 1.000 | 1.000 | 0 | 0.529 |
| *Paraburkholderia kururiensis* | 1.15 (0.36-3.69) | 0.814 | 0 | 1.000 | 1.000 | 58.7 | 0.120 | 0.48 (0.05-4.66) | 0.530 | 0 | 1.000 | 1.000 | 87.8 | 0.004 |
| *Tepidimonas taiwanensis* | 0.98 (0.86-1.13) | 0.818 | 0 | 1.000 | 1.000 | 7.8 | 0.298 | 1.01 (0.9-1.14) | 0.815 | 0 | 1.000 | 1.000 | 0 | 0.382 |
| *[Propionibacterium] namnetense* | 0.98 (0.86-1.13) | 0.822 | 0 | 1.000 | 1.000 | 21.4 | 0.259 | 1.06 (0.8-1.39) | 0.691 | 0 | 1.000 | 1.000 | 68.3 | 0.076 |
| *Cytophagales bacterium B6* | 0.98 (0.86-1.13) | 0.822 | 0 | 1.000 | 1.000 | 12.4 | 0.285 | 0.99 (0.89-1.09) | 0.785 | 0 | 1.000 | 1.000 | 0 | 0.420 |
| *Thermoanaerobacter wiegelii* | 0.94 (0.56-1.58) | 0.824 | 0 | 1.000 | 1.000 | 0 | 0.934 | 0.69 (0.43-1.1) | 0.115 | 0 | 1.000 | 1.000 | 0 | 0.533 |
| *Corynebacterium matruchotii* | 0.96 (0.65-1.41) | 0.825 | 0 | 1.000 | 1.000 | 59.7 | 0.115 | 0.96 (0.69-1.34) | 0.819 | 0 | 1.000 | 1.000 | 52.5 | 0.147 |
| *Gemmata obscuriglobus* | 1.01 (0.93-1.1) | 0.830 | 0 | 1.000 | 1.000 | 0 | 0.841 | 1.1 (0.95-1.28) | 0.209 | 2 | 1.000 | 1.000 | 67.7 | 0.079 |
| *Geobacillus subterraneus* | 1.08 (0.52-2.24) | 0.831 | 1 | 1.000 | 1.000 | 45.8 | 0.174 | 0.87 (0.76-1) | **0.044** | 42 | 1.000 | 0.012 | 0 | 0.671 |
| *Sphingomonas sp. Ag1* | 0.96 (0.67-1.38) | 0.836 | 0 | 1.000 | 1.000 | 58.6 | 0.120 | 0.93 (0.76-1.15) | 0.518 | 0 | 1.000 | 1.000 | 4.6 | 0.306 |
| *Calothrix sp. PCC 7103* | 0.93 (0.47-1.85) | 0.838 | 0 | 1.000 | 1.000 | 74.2 | 0.049 | 0.91 (0.43-1.93) | 0.801 | 0 | 1.000 | 1.000 | 80.3 | 0.024 |
| *Agrobacterium arsenijevicii* | 0.98 (0.82-1.18) | 0.839 | 0 | 1.000 | 1.000 | 0 | 0.528 | 0.9 (0.77-1.06) | 0.203 | 1 | 1.000 | 1.000 | 0 | 0.538 |
| *Peptoniphilus rhinitidis* | 1.02 (0.86-1.2) | 0.841 | 0 | 1.000 | 1.000 | 0 | 0.699 | 1.03 (0.89-1.21) | 0.673 | 0 | 1.000 | 1.000 | 0 | 0.454 |
| *Thermoanaerobacter sp. YS13* | 1.12 (0.37-3.4) | 0.841 | 0 | 1.000 | 1.000 | 53.9 | 0.141 | 0.46 (0.05-3.95) | 0.478 | 0 | 1.000 | 1.000 | 86.4 | 0.007 |
| *Curvibacter lanceolatus* | 0.98 (0.83-1.17) | 0.841 | 0 | 1.000 | 1.000 | 66.4 | 0.085 | 0.97 (0.86-1.09) | 0.592 | 1 | 1.000 | 0.453 | 46 | 0.173 |
| *Acinetobacter gerneri* | 1.02 (0.85-1.22) | 0.855 | 0 | 1.000 | 1.000 | 0 | 0.642 | 0.97 (0.82-1.14) | 0.720 | 0 | 1.000 | 1.000 | 0 | 0.416 |
| *Aquabacterium sp. NJ1* | 0.98 (0.77-1.25) | 0.858 | 0 | 1.000 | 1.000 | 25.8 | 0.246 | 1.06 (0.87-1.3) | 0.549 | 0 | 1.000 | 1.000 | 0 | 0.508 |
| *Bacillus anthracis* | 1.1 (0.39-3.12) | 0.859 | 0 | 1.000 | 1.000 | 49.4 | 0.160 | 0.46 (0.05-3.99) | 0.482 | 0 | 1.000 | 1.000 | 86.6 | 0.006 |
| *Bacillus sp. FJAT-14578* | 1.1 (0.39-3.12) | 0.859 | 0 | 1.000 | 1.000 | 49.4 | 0.160 | 0.46 (0.05-3.99) | 0.482 | 0 | 1.000 | 1.000 | 86.6 | 0.006 |
| *Microcystis aeruginosa* | 1.1 (0.39-3.12) | 0.859 | 0 | 1.000 | 1.000 | 49.4 | 0.160 | 0.46 (0.05-3.99) | 0.482 | 0 | 1.000 | 1.000 | 86.6 | 0.006 |
| *Mycoplasma cricetuli* | 1.1 (0.39-3.12) | 0.859 | 0 | 1.000 | 1.000 | 49.4 | 0.160 | 0.46 (0.05-3.99) | 0.482 | 0 | 1.000 | 1.000 | 86.6 | 0.006 |
| *Mycoplasma testudinis* | 1.1 (0.39-3.12) | 0.859 | 0 | 1.000 | 1.000 | 49.4 | 0.160 | 0.46 (0.05-3.99) | 0.482 | 0 | 1.000 | 1.000 | 86.6 | 0.006 |
| *Streptomyces ghanaensis* | 1.1 (0.39-3.12) | 0.859 | 0 | 1.000 | 1.000 | 49.4 | 0.160 | 0.46 (0.05-3.99) | 0.482 | 0 | 1.000 | 1.000 | 86.6 | 0.006 |
| *Streptomyces lividans* | 1.1 (0.39-3.12) | 0.859 | 0 | 1.000 | 1.000 | 49.4 | 0.160 | 0.46 (0.05-3.99) | 0.482 | 0 | 1.000 | 1.000 | 86.6 | 0.006 |
| *Prevotella copri* | 1.02 (0.83-1.25) | 0.865 | 1 | 0.858 | 1.000 | 0 | 0.554 | 0.95 (0.71-1.27) | 0.729 | 0 | 0.858 | 1.000 | 37 | 0.208 |
| *Sphingobium yanoikuyae* | 0.98 (0.79-1.22) | 0.871 | 0 | 1.000 | 1.000 | 60.3 | 0.112 | 1 (0.89-1.13) | 0.962 | 0 | 1.000 | 1.000 | 0 | 0.952 |
| *Staphylococcus haemolyticus* | 1.01 (0.9-1.14) | 0.876 | 0 | 1.000 | 1.000 | 0 | 0.496 | 1.01 (0.9-1.13) | 0.869 | 0 | 1.000 | 1.000 | 0 | 0.784 |
| *Caballeronia zhejiangensis* | 1.08 (0.42-2.78) | 0.881 | 0 | 1.000 | 1.000 | 74.5 | 0.048 | 1.17 (0.75-1.82) | 0.486 | 0 | 1.000 | 1.000 | 0 | 0.596 |
| *Acinetobacter bereziniae* | 1.03 (0.68-1.57) | 0.886 | 19 | 0.002 | 0.016 | 79 | 0.029 | 1 (0.65-1.54) | 0.985 | 13 | 0.002 | 0.012 | 81.7 | 0.019 |
| *Sphingobium lactosutens* | 0.99 (0.84-1.16) | 0.890 | 0 | 1.000 | 1.000 | 58 | 0.123 | 0.93 (0.81-1.07) | 0.323 | 5 | 1.000 | 0.012 | 42.4 | 0.187 |
| *Necropsobacter massiliensis* | 0.99 (0.9-1.09) | 0.892 | 0 | 1.000 | 1.000 | 51.6 | 0.151 | 0.98 (0.88-1.09) | 0.755 | 0 | 1.000 | 1.000 | 66.7 | 0.083 |
| *Williamsia muralis* | 1.02 (0.78-1.32) | 0.893 | 0 | 1.000 | 1.000 | 0 | 0.657 | 1.08 (0.86-1.36) | 0.516 | 0 | 1.000 | 1.000 | 0 | 0.550 |
| *Dermacoccus nishinomiyaensis* | 1.01 (0.85-1.2) | 0.893 | 0 | 1.000 | 1.000 | 0 | 0.418 | 1.05 (0.9-1.21) | 0.549 | 0 | 1.000 | 1.000 | 0 | 0.892 |
| *Corynebacterium simulans* | 1.02 (0.81-1.27) | 0.893 | 0 | 1.000 | 1.000 | 0 | 0.335 | 1.03 (0.85-1.24) | 0.784 | 0 | 1.000 | 1.000 | 0 | 0.793 |
| *Pseudomonas mendocina* | 1.01 (0.88-1.16) | 0.894 | 0 | 1.000 | 1.000 | 0 | 0.865 | 1.09 (0.96-1.23) | 0.195 | 1 | 1.000 | 1.000 | 0 | 0.512 |
| *Pseudomonas putida* | 0.98 (0.74-1.3) | 0.897 | 0 | 1.000 | 1.000 | 24.4 | 0.250 | 0.94 (0.61-1.47) | 0.797 | 0 | 1.000 | 1.000 | 68 | 0.077 |
| *Thermus scotoductus* | 1.01 (0.81-1.26) | 0.901 | 0 | 1.000 | 1.000 | 0 | 0.693 | 0.97 (0.8-1.17) | 0.731 | 0 | 1.000 | 1.000 | 0 | 0.950 |
| *Massilia sp. NR 4-1* | 1.02 (0.73-1.42) | 0.908 | 0 | 1.000 | 1.000 | 33.2 | 0.221 | 0.94 (0.66-1.33) | 0.720 | 0 | 1.000 | 1.000 | 40.5 | 0.195 |
| *Frigoribacterium sp. Leaf254* | 1.02 (0.74-1.4) | 0.915 | 0 | 1.000 | 1.000 | 31.4 | 0.227 | 1.08 (0.89-1.32) | 0.432 | 0 | 1.000 | 1.000 | 0 | 0.616 |
| *Deinococcus gobiensis* | 0.97 (0.52-1.82) | 0.931 | 0 | 1.000 | 1.000 | 77.8 | 0.034 | 0.95 (0.6-1.5) | 0.825 | 0 | 1.000 | 1.000 | 66.3 | 0.085 |
| *Staphylococcus pettenkoferi* | 0.98 (0.64-1.5) | 0.932 | 0 | 1.000 | 1.000 | 55.2 | 0.135 | 0.89 (0.69-1.15) | 0.382 | 0 | 1.000 | 1.000 | 0 | 0.554 |
| *Moraxella osloensis* | 0.99 (0.86-1.15) | 0.934 | 0 | 1.000 | 1.000 | 50.1 | 0.157 | 1.03 (0.94-1.12) | 0.555 | 0 | 1.000 | 1.000 | 9.8 | 0.292 |
| *Acinetobacter radioresistens* | 1.01 (0.8-1.27) | 0.935 | 0 | 1.000 | 1.000 | 29.8 | 0.233 | 0.97 (0.7-1.35) | 0.867 | 0 | 1.000 | 1.000 | 44.9 | 0.178 |
| *Enterobacter cloacae* | 1 (0.91-1.09) | 0.937 | 0 | 1.000 | 1.000 | 0 | 0.767 | 1.02 (0.94-1.1) | 0.698 | 0 | 1.000 | 1.000 | 0 | 0.814 |
| *Sphingomonas sp. Leaf11* | 0.99 (0.73-1.33) | 0.939 | 0 | 1.000 | 1.000 | 0 | 0.664 | 0.95 (0.72-1.26) | 0.745 | 0 | 1.000 | 1.000 | 0 | 0.583 |
| *Phenylobacterium sp. Root700* | 0.97 (0.43-2.18) | 0.943 | 0 | 1.000 | 1.000 | 69.6 | 0.070 | 1.16 (0.83-1.63) | 0.377 | 0 | 1.000 | 1.000 | 0 | 0.459 |
| *Novosphingobium malaysiense* | 0.99 (0.86-1.15) | 0.944 | 0 | 1.000 | 1.000 | 0 | 0.567 | 0.98 (0.86-1.11) | 0.711 | 0 | 1.000 | 1.000 | 0 | 0.414 |
| *Pseudomonas saudiphocaensis* | 0.99 (0.79-1.25) | 0.946 | 0 | 1.000 | 1.000 | 27.5 | 0.240 | 0.95 (0.62-1.45) | 0.814 | 0 | 1.000 | 1.000 | 74.3 | 0.048 |
| *Curvibacter sp. PAE-UM* | 0.99 (0.74-1.32) | 0.947 | 0 | 1.000 | 1.000 | 0 | 0.687 | 0.93 (0.71-1.22) | 0.622 | 0 | 1.000 | 1.000 | 0 | 0.623 |
| *[Bacillus] aminovorans* | 1.01 (0.75-1.37) | 0.947 | 0 | 1.000 | 1.000 | 71.7 | 0.060 | 1.01 (0.73-1.42) | 0.932 | 0 | 1.000 | 1.000 | 82.5 | 0.017 |
| *Corynebacterium jeikeium* | 1.01 (0.8-1.26) | 0.955 | 0 | 1.000 | 1.000 | 66.4 | 0.084 | 1.03 (0.94-1.13) | 0.488 | 0 | 1.000 | 1.000 | 0 | 0.831 |
| *Thalassobacter stenotrophicus* | 1 (0.92-1.08) | 0.957 | 0 | 1.000 | 1.000 | 0 | 0.769 | 1 (0.92-1.07) | 0.921 | 0 | 1.000 | 1.000 | 0 | 0.348 |
| *Roseomonas gilardii* | 0.99 (0.76-1.3) | 0.960 | 0 | 1.000 | 1.000 | 0 | 0.871 | 0.95 (0.61-1.48) | 0.810 | 0 | 1.000 | 1.000 | 42.6 | 0.187 |
| *Dietzia maris* | 0.99 (0.76-1.29) | 0.961 | 0 | 1.000 | 1.000 | 0 | 0.403 | 1 (0.78-1.29) | 0.985 | 0 | 1.000 | 1.000 | 0 | 0.320 |
| *Acinetobacter schindleri* | 1 (0.85-1.19) | 0.963 | 0 | 1.000 | 1.000 | 46.6 | 0.171 | 1.05 (0.96-1.16) | 0.302 | 0 | 1.000 | 1.000 | 0 | 0.702 |
| *Comamonas granuli* | 1.01 (0.78-1.29) | 0.966 | 0 | 1.000 | 1.000 | 0 | 0.948 | 0.98 (0.77-1.23) | 0.833 | 0 | 1.000 | 1.000 | 0 | 0.882 |
| *Chryseobacterium sp. JAH* | 0.99 (0.67-1.48) | 0.968 | 1 | 0.980 | 1.000 | 72.8 | 0.055 | 1.07 (0.92-1.26) | 0.386 | 0 | 0.980 | 1.000 | 11.5 | 0.288 |
| *Gordonia polyisoprenivorans* | 1 (0.86-1.17) | 0.972 | 0 | 1.000 | 1.000 | 0 | 0.544 | 0.99 (0.87-1.13) | 0.901 | 0 | 1.000 | 1.000 | 0 | 0.876 |
| *Bacteroides vulgatus* | 1 (0.83-1.22) | 0.975 | 0 | 1.000 | 1.000 | 0 | 0.725 | 0.91 (0.75-1.1) | 0.319 | 0 | 1.000 | 1.000 | 0 | 0.342 |
| *Sphingomonas echinoides* | 0.99 (0.64-1.55) | 0.976 | 0 | 1.000 | 1.000 | 82.9 | 0.015 | 0.92 (0.67-1.28) | 0.640 | 9 | 1.000 | 1.000 | 70.2 | 0.067 |
| *Streptococcus thermophilus* | 1 (0.87-1.15) | 0.978 | 0 | 1.000 | 1.000 | 0 | 0.418 | 1.04 (0.92-1.17) | 0.555 | 0 | 1.000 | 1.000 | 0 | 0.901 |
| *Paracoccus sphaerophysae* | 1 (0.79-1.28) | 0.978 | 0 | 1.000 | 1.000 | 0 | 0.686 | 1 (0.8-1.24) | 0.968 | 0 | 1.000 | 1.000 | 0 | 0.946 |
| *Actinomyces graevenitzii* | 1 (0.89-1.13) | 0.978 | 0 | 1.000 | 1.000 | 0 | 0.722 | 0.99 (0.89-1.1) | 0.803 | 0 | 1.000 | 1.000 | 0 | 0.887 |
| *Paraburkholderia xenovorans* | 1 (0.81-1.23) | 0.982 | 0 | 1.000 | 1.000 | 0 | 0.382 | 1.16 (0.98-1.37) | 0.083 | 14 | 1.000 | 0.012 | 0 | 0.973 |
| *Novosphingobium sp. Rr 2-17* | 1 (0.9-1.12) | 0.988 | 0 | 1.000 | 1.000 | 0 | 0.412 | 0.98 (0.89-1.08) | 0.671 | 0 | 1.000 | 1.000 | 0 | 0.350 |
| *Acidovorax avenae* | 1 (0.7-1.43) | 0.988 | 0 | 1.000 | 1.000 | 0 | 0.821 | 0.83 (0.6-1.16) | 0.280 | 0 | 1.000 | 1.000 | 0 | 0.990 |
| *Geobacillus thermoleovorans* | 1 (0.81-1.23) | 0.989 | 0 | 1.000 | 1.000 | 0 | 0.320 | 0.54 (0.14-2.16) | 0.384 | 0 | 1.000 | 1.000 | 78.9 | 0.030 |
| *Delftia acidovorans* | 1 (0.82-1.23) | 0.995 | 0 | 1.000 | 1.000 | 0 | 0.821 | 1.04 (0.87-1.25) | 0.646 | 0 | 1.000 | 1.000 | 0 | 0.771 |
| *Caulobacter henricii* | 1 (0.72-1.39) | 0.996 | 0 | 1.000 | 1.000 | 17.7 | 0.270 | 1.1 (0.84-1.45) | 0.492 | 0 | 1.000 | 1.000 | 0 | 0.484 |
| *Roseateles depolymerans* | 1 (0.8-1.24) | 1.000 | 0 | 1.000 | 1.000 | 0 | 0.882 | 1.06 (0.86-1.31) | 0.592 | 0 | 1.000 | 1.000 | 0 | 0.347 |

^a^ Multivariate model adjusting for age, gender, tumor stage, and cigarette smoking in Cox proportional hazards models.

^b^ Random-effect Meta-analysis of Cox proportional hazards models was performed with repeated subsampling (90% of the samples, 100 iterations), and the selected proportion out of 100 for each taxon in the meta-analysis of TCGA and ICGC is shown.

^c^ Permutation test column represents the results based on a permutation test with 500 iterations. The *P*-value for *j*th species was calculated as $\frac{\sum_{i}^{500} {I(P}_{ij}\geq O_{j})+1}{501}$, where $I(\cdot)$ is the indicator function, $O_{j}$ was the frequency of *j*th species identified (i.e., with a meta p-value of <0.05 in differential abundance test) using the original cohorts, and $P_{ij}$ was the frequency of *j*th species identified using the permuted data in *i*th iteration. In each iteration, the permuted data was generated by randomly permuting the sample ID. The *Q*-value was then calculated adjusting for the false discovery rate (FDR) across all species.

^d^ Heterogeneity was quantified using *I*^2^ and its significance was tested to assess whether there were significant differences among the subgroups based on cohort.

**Supplemental Table 3. Association of tumor microbial modules and pathways with early-stage PDAC OS and RFS in the meta-analysis of TCGA and ICGC cohorts.**

| **KEGG Module Name** | Meta-analysis for OS^a^ | | | | |  | Meta-analysis for RFS^a^ | | | | | | | |
| --- | --- | --- | --- | --- | --- | --- | --- | --- | --- | --- | --- | --- | --- | --- |
|  | HR (95%CI) | *P*_meta_ | *I*^2^ | *P*_het_ | |  | HR (95%CI) | | *P*_meta_ | | *I*^2^ | | *P*_het_ | |
| M00156: Cytochrome c oxidase, cbb3-type | **0.82 (0.73-0.92)** | **4.37E-04** | 0.0 | 0.442 | |  | **0.87 (0.78-0.96)** | | **6.48E-03** | | 0.0 | | 0.713 | |
| M00201: alpha-Glucoside transport system | **1.27 (1.08-1.49)** | **3.38E-03** | 0.0 | 0.401 | |  | **1.21 (1.05-1.39)** | | **8.11E-03** | | 0.0 | | 0.520 | |
| M00514: TtrS-TtrR (tetrathionate respiration) two-component regulatory system | **0.85 (0.75-0.96)** | **7.50E-03** | 0.0 | 0.682 | |  | 0.88 (0.74-1.04) | | 0.135 | | 52.5 | | 0.147 | |
| M00185: Sulfate transport system | **0.83 (0.72-0.96)** | **0.012** | 0.0 | 0.363 | |  | **0.86 (0.75-0.98)** | | **0.025** | | 0.0 | | 0.687 | |
| M00080: Lipopolysaccharide biosynthesis, inner core => outer core => O-antigen | **0.85 (0.75-0.97)** | **0.013** | 0.0 | 0.498 | |  | 0.89 (0.79-1) | | 0.060 | | 0.0 | | 0.999 | |
| M00064: ADP-L-glycero-D-manno-heptose biosynthesis | **0.85 (0.74-0.97)** | **0.019** | 0.0 | 0.847 | |  | **0.87 (0.77-0.99)** | | **0.032** | | 0.0 | | 0.716 | |
| M00523: RegB-RegA (redox response) two-component regulatory system | **0.83 (0.71-0.97)** | **0.020** | 0.0 | 0.467 | |  | **0.85 (0.74-0.98)** | | **0.023** | | 0.0 | | 0.895 | |
| M00042: Catecholamine biosynthesis, tyrosine => dopamine => noradrenaline => adrenaline | **1.22 (1.03-1.44)** | **0.022** | 0.0 | 0.436 | |  | **1.21 (1.04-1.41)** | | **0.014** | | 0.0 | | 0.827 | |
| M00230: Glutamate/aspartate transport system | **0.9 (0.83-0.99)** | **0.025** | 0.0 | 0.516 | |  | **0.9 (0.83-0.97)** | | **7.83E-03** | | 0.0 | | 0.448 | |
| M00066: Lactosylceramide biosynthesis | **1.11 (1.01-1.21)** | **0.025** | 0.0 | 0.444 | |  | **1.1 (1.01-1.19)** | | **0.021** | | 0.0 | | 0.956 | |
| M00302: 2-Aminoethylphosphonate transport system | **1.1 (1.01-1.21)** | **0.027** | 0.0 | 0.601 | |  | **1.14 (1.05-1.24)** | | **2.58E-03** | | 0.0 | | 0.842 | |
| M00442: Putative hydroxymethylpyrimidine transport system | **1.11 (1.01-1.22)** | **0.028** | 0.0 | 0.909 | |  | 1.09 (1-1.19) | | 0.050 | | 0.0 | | 0.372 | |
| M00276: PTS system, mannose-specific II component | **1.15 (1.01-1.3)** | **0.029** | 0.0 | 0.323 | |  | 1.07 (0.96-1.2) | | 0.216 | | 0.0 | | 0.870 | |
| M00047: Creatine pathway | **1.16 (1.01-1.34)** | **0.036** | 0.0 | 0.695 | |  | 1.17 (0.99-1.38) | | 0.066 | | 28.3 | | 0.237 | |
| M00251: Teichoic acid transport system | **1.15 (1.01-1.31)** | **0.036** | 0.0 | 0.561 | |  | **1.17 (1.04-1.31)** | | **7.57E-03** | | 0.0 | | 0.543 | |
| M00250: Lipopolysaccharide transport system | **0.89 (0.79-1)** | **0.043** | 0.0 | 0.743 | |  | 0.94 (0.84-1.04) | | 0.245 | | 0.0 | | 0.808 | |
| M00501: PilS-PilR (type 4 fimbriae synthesis) two-component regulatory system | **0.89 (0.79-1)** | **0.046** | 0.0 | 0.422 | |  | 0.94 (0.84-1.04) | | 0.237 | | 0.0 | | 0.366 | |
| M00039: Monolignol biosynthesis, phenylalanine/tyrosine => monolignol | 0.88 (0.77-1) | 0.050 | 0.0 | 0.521 | |  | 0.92 (0.82-1.04) | | 0.200 | | 0.0 | | 0.821 | |
| M00253: Sodium transport system | 0.89 (0.79-1) | 0.050 | 9.9 | 0.292 | |  | 0.96 (0.87-1.05) | | 0.349 | | 0.0 | | 0.484 | |
| M00365: C10-C20 isoprenoid biosynthesis, archaea | 1.21 (1-1.46) | 0.055 | 0.0 | 0.668 | |  | 1.16 (0.98-1.37) | | 0.078 | | 0.0 | | 0.860 | |
| M00493: AlgZ-AlgR (alginate production) two-component regulatory system | 0.9 (0.8-1) | 0.058 | 0.0 | 0.332 | |  | 0.95 (0.86-1.05) | | 0.291 | | 0.0 | | 0.370 | |
| M00416: Cytochrome aa3-600 menaquinol oxidase | 0.94 (0.88-1) | 0.061 | 0.0 | 0.584 | |  | **0.94 (0.89-1)** | | **0.047** | | 0.0 | | 0.700 | |
| M00479: DesK-DesR (membrane lipid fluidity regulation) two-component regulatory system | 1.18 (0.99-1.4) | 0.066 | 0.0 | 0.942 | |  | 1.13 (0.97-1.31) | | 0.111 | | 0.0 | | 0.715 | |
| M00107: Steroid hormone biosynthesis, cholesterol => prognenolone => progesterone | 0.9 (0.8-1.01) | 0.066 | 0.0 | 0.485 | |  | 0.93 (0.84-1.04) | | 0.199 | | 0.0 | | 0.530 | |
| M00063: CMP-KDO biosynthesis | 0.85 (0.71-1.02) | 0.079 | 0.0 | 0.594 | |  | 0.87 (0.74-1.03) | | 0.112 | | 0.0 | | 0.680 | |
| M00213: L-Arabinose transport system | 1.09 (0.99-1.21) | 0.080 | 0.0 | 0.584 | |  | 1.08 (0.98-1.18) | | 0.106 | | 0.0 | | 0.383 | |
| M00110: C19/C18-Steroid hormone biosynthesis, pregnenolone => androstenedione => estrone | 0.9 (0.8-1.01) | 0.083 | 0.0 | 0.447 | |  | 0.93 (0.83-1.03) | | 0.170 | | 0.0 | | 0.568 | |
| M00191: Thiamine transport system | 1.07 (0.99-1.16) | 0.085 | 0.0 | 0.666 | |  | **1.08 (1.01-1.16)** | | **0.020** | | 0.0 | | 0.352 | |
| M00349: Microcin C transport system | 0.93 (0.85-1.01) | 0.090 | 0.0 | 0.428 | |  | 0.95 (0.86-1.06) | | 0.361 | | 34.3 | | 0.217 | |
| M00338: Cysteine biosynthesis, homocysteine + serine => cysteine | 1.11 (0.98-1.26) | 0.091 | 16.9 | 0.273 | |  | 1.11 (1-1.24) | | 0.055 | | 11.1 | | 0.289 | |
| M00543: Biphenyl degradation, biphenyl => 2-oxopent-4-enoate + benzoate | 1.11 (0.98-1.24) | 0.092 | 34.7 | 0.216 | |  | 1.13 (0.97-1.32) | | 0.117 | | 66.9 | | 0.082 | |
| M00366: C10-C20 isoprenoid biosynthesis, plants | 1.21 (0.97-1.51) | 0.092 | 0.0 | 0.905 | |  | 1.15 (0.94-1.4) | | 0.176 | | 0.0 | | 0.646 | |
| M00547: Benzene/toluene degradation, benzene => catechol / toluene => 3-methylcatechol | 1.1 (0.98-1.25) | 0.101 | 37.0 | 0.208 | |  | 1.13 (0.97-1.31) | | 0.105 | | 65.4 | | 0.089 | |
| M00091: Phosphatidylcholine (PC) biosynthesis, PE => PC | 1.11 (0.98-1.27) | 0.103 | 0.0 | 0.732 | |  | 1.09 (0.96-1.22) | | 0.176 | | 0.0 | | 0.844 | |
| M00471: NarX-NarL (nitrate respiration) two-component regulatory system | 0.92 (0.84-1.02) | 0.103 | 0.0 | 0.659 | |  | 0.92 (0.84-1.01) | | 0.089 | | 0.0 | | 0.648 | |
| M00342: Bacterial proteasome | 1.16 (0.97-1.39) | 0.103 | 0.0 | 0.690 | |  | 1.15 (0.97-1.35) | | 0.102 | | 0.0 | | 0.714 | |
| M00506: CheA-CheYBV (chemotaxis) two-component regulatory system | 0.74 (0.51-1.07) | 0.108 | 34.4 | 0.217 | |  | 0.76 (0.52-1.12) | | 0.161 | | 47.4 | | 0.168 | |
| M00206: Cellobiose transport system | 1.19 (0.96-1.47) | 0.110 | 51.6 | 0.151 | |  | 1.14 (0.95-1.37) | | 0.154 | | 47.4 | | 0.168 | |
| M00458: ResE-ResD (aerobic and anaerobic respiration) two-component regulatory system | 0.94 (0.88-1.01) | 0.111 | 0.0 | 0.729 | |  | 0.94 (0.89-1.01) | | 0.076 | | 0.0 | | 0.428 | |
| M00122: Cobalamin biosynthesis, cobinamide => cobalamin | 0.8 (0.61-1.05) | 0.112 | 0.0 | 0.769 | |  | 0.86 (0.67-1.09) | | 0.207 | | 0.0 | | 0.881 | |
| M00364: C10-C20 isoprenoid biosynthesis, bacteria | 1.22 (0.95-1.57) | 0.116 | 0.0 | 0.782 | |  | 1.16 (0.92-1.45) | | 0.207 | | 0.0 | | 0.653 | |
| M00440: Nickel transport system | 1.08 (0.98-1.18) | 0.117 | 0.0 | 0.549 | |  | 1.06 (0.97-1.15) | | 0.199 | | 0.0 | | 0.801 | |
| M00113: Jasmonic acid biosynthesis | 1.16 (0.96-1.41) | 0.118 | 0.0 | 0.484 | |  | 1.1 (0.88-1.37) | | 0.422 | | 42.1 | | 0.189 | |
| M00443: SenX3-RegX3 (phosphate starvation response) two-component regulatory system | 1.15 (0.96-1.38) | 0.126 | 0.0 | 0.806 | |  | 1.13 (0.96-1.33) | | 0.129 | | 0.0 | | 0.876 | |
| M00210: Phospholipid transport system | 0.88 (0.74-1.04) | 0.144 | 18.2 | 0.269 | |  | 0.9 (0.79-1.02) | | 0.091 | | 0.0 | | 0.402 | |
| M00492: LytS-LytR two-component regulatory system | 1.09 (0.97-1.22) | 0.144 | 0.0 | 0.503 | |  | 1.04 (0.86-1.25) | | 0.717 | | 69.3 | | 0.071 | |
| M00237: Branched-chain amino acid transport system | 0.89 (0.75-1.04) | 0.148 | 0.0 | 0.547 | |  | 0.95 (0.75-1.2) | | 0.678 | | 23.6 | | 0.253 | |
| M00433: Lysine biosynthesis, 2-oxoglutarate => 2-oxoadipate | 1.12 (0.96-1.3) | 0.148 | 0.0 | 0.495 | |  | 1.11 (0.86-1.42) | | 0.435 | | 65.9 | | 0.087 | |
| M00231: Octopine/nopaline transport system | 1.07 (0.97-1.18) | 0.153 | 0.0 | 0.471 | |  | **1.09 (1-1.2)** | | **0.045** | | 0.0 | | 0.826 | |
| M00202: Oligogalacturonide transport system | 1.08 (0.97-1.21) | 0.153 | 0.0 | 0.911 | |  | 1.02 (0.86-1.22) | | 0.812 | | 67.2 | | 0.081 | |
| M00343: Archaeal proteasome | 1.13 (0.95-1.36) | 0.164 | 0.0 | 0.391 | |  | 1.06 (0.91-1.24) | | 0.447 | | 0.0 | | 0.384 | |
| M00060: Lipopolysaccharide biosynthesis, KDO2-lipid A | 0.91 (0.79-1.04) | 0.167 | 0.0 | 0.698 | |  | 0.95 (0.83-1.08) | | 0.416 | | 0.0 | | 0.522 | |
| M00332: Type III secretion system | 1.04 (0.98-1.1) | 0.168 | 0.0 | 0.602 | |  | 1.05 (0.99-1.1) | | 0.084 | | 0.0 | | 0.596 | |
| M00044: Tyrosine degradation, tyrosine => homogentisate | 0.92 (0.81-1.04) | 0.173 | 0.0 | 0.357 | |  | 0.96 (0.85-1.08) | | 0.452 | | 0.0 | | 0.381 | |
| M00209: Osmoprotectant transport system | 1.18 (0.93-1.49) | 0.177 | 62.5 | 0.103 | |  | 1.18 (0.91-1.52) | | 0.217 | | 71.0 | | 0.063 | |
| M00323: Urea transport system | 0.93 (0.85-1.03) | 0.182 | 5.8 | 0.303 | |  | 1 (0.87-1.15) | | 0.964 | | 41.4 | | 0.191 | |
| M00372: Abscisic acid biosynthesis, beta-carotene => abscisic acid | 1.08 (0.96-1.22) | 0.182 | 0.0 | 0.684 | |  | 1.07 (0.96-1.18) | | 0.222 | | 0.0 | | 0.690 | |
| M00507: ChpA-ChpB/PilGH (chemosensory) two-component regulatory system | 0.92 (0.82-1.04) | 0.184 | 12.4 | 0.285 | |  | 0.96 (0.86-1.06) | | 0.394 | | 9.0 | | 0.295 | |
| M00367: C10-C20 isoprenoid biosynthesis, non-plant eukaryotes | 1.13 (0.94-1.36) | 0.187 | 0.0 | 0.585 | |  | 1.12 (0.96-1.32) | | 0.151 | | 0.0 | | 0.690 | |
| M00279: PTS system, galactitol-specific II component | 1.08 (0.96-1.21) | 0.193 | 0.0 | 0.366 | |  | 1.05 (0.89-1.23) | | 0.590 | | 60.2 | | 0.113 | |
| M00220: Rhamnose transport system | 1.08 (0.96-1.21) | 0.196 | 0.0 | 0.778 | |  | 1.06 (0.95-1.18) | | 0.284 | | 0.0 | | 0.834 | |
| M00545: Trans-cinnamate degradation, trans-cinnamate => acetyl-CoA | 1.08 (0.96-1.22) | 0.199 | 13.2 | 0.283 | |  | **1.13 (1.02-1.25)** | | **0.021** | | 0.0 | | 0.350 | |
| M00045: Histidine degradation, histidine => N-formiminoglutamate => glutamate | 1.14 (0.93-1.4) | 0.200 | 0.0 | 0.824 | |  | 1.13 (0.95-1.35) | | 0.166 | | 0.0 | | 0.917 | |
| M00446: RstB-RstA two-component regulatory system | 0.94 (0.85-1.04) | 0.204 | 0.0 | 0.377 | |  | 0.97 (0.89-1.06) | | 0.534 | | 0.0 | | 0.882 | |
| M00502: GlrK-GlrR (amino sugar metabolism) two-component regulatory system | 0.94 (0.85-1.04) | 0.205 | 0.0 | 0.920 | |  | 0.94 (0.86-1.04) | | 0.232 | | 0.0 | | 0.910 | |
| M00520: ChvG-ChvI (acidity sensing) two-component regulatory system | 1.09 (0.95-1.24) | 0.206 | 0.0 | 0.377 | |  | 1.02 (0.91-1.15) | | 0.681 | | 0.0 | | 0.343 | |
| M00040: Tyrosine biosynthesis, prephanate => pretyrosine => tyrosine | 0.92 (0.81-1.05) | 0.208 | 0.0 | 0.543 | |  | 0.95 (0.85-1.07) | | 0.426 | | 0.0 | | 0.738 | |
| M00542: EHEC/EPEC pathogenicity signature, T3SS and effectors | 1.04 (0.98-1.1) | 0.210 | 0.0 | 0.555 | |  | 1.04 (0.99-1.1) | | 0.102 | | 0.0 | | 0.564 | |
| M00434: PhoR-PhoB (phosphate starvation response) two-component regulatory system | 0.86 (0.67-1.09) | 0.211 | 18.1 | 0.269 | |  | 0.87 (0.7-1.06) | | 0.170 | | 5.9 | | 0.303 | |
| M00476: ComP-ComA (competence) two-component regulatory system | 0.93 (0.83-1.04) | 0.215 | 0.0 | 0.328 | |  | 0.94 (0.83-1.07) | | 0.368 | | 24.0 | | 0.251 | |
| M00445: EnvZ-OmpR (osmotic stress response) two-component regulatory system | 0.93 (0.82-1.05) | 0.230 | 15.0 | 0.278 | |  | 0.93 (0.84-1.03) | | 0.184 | | 5.5 | | 0.304 | |
| M00569: Catechol meta-cleavage, catechol => acetyl-CoA / 4-methylcatechol => propanoyl-CoA | 0.88 (0.71-1.09) | 0.231 | 32.9 | 0.222 | |  | 0.92 (0.79-1.06) | | 0.248 | | 0.0 | | 0.407 | |
| M00128: Ubiquinone biosynthesis, eukaryotes, 4-hydroxybenzoate => ubiquinone | 0.87 (0.7-1.09) | 0.234 | 55.1 | 0.136 | |  | **0.89 (0.79-1)** | | **0.047** | | 0.0 | | 0.444 | |
| M00233: Glutamate transport system | 1.1 (0.94-1.3) | 0.236 | 0.0 | 0.538 | |  | 1.12 (0.97-1.29) | | 0.137 | | 0.0 | | 0.587 | |
| M00549: Nucleotide sugar biosynthesis, glucose => UDP-glucose | 1.22 (0.88-1.7) | 0.237 | 10.0 | 0.292 | |  | 1.1 (0.83-1.45) | | 0.505 | | 0.0 | | 0.525 | |
| M00235: Arginine/ornithine transport system | 0.95 (0.87-1.04) | 0.244 | 0.0 | 0.675 | |  | 0.97 (0.89-1.05) | | 0.426 | | 0.0 | | 0.819 | |
| M00211: Putative ABC transport system | 1.07 (0.95-1.2) | 0.247 | 0.0 | 0.948 | |  | 0.99 (0.9-1.1) | | 0.917 | | 0.0 | | 0.350 | |
| M00135: GABA biosynthesis, eukaryotes, putrescine => GABA | 0.92 (0.79-1.07) | 0.266 | 25.0 | 0.248 | |  | 0.97 (0.87-1.09) | | 0.647 | | 0.0 | | 0.493 | |
| M00145: NAD(P)H:quinone oxidoreductase, chloroplasts and cyanobacteria | 1.06 (0.95-1.19) | 0.276 | 0.0 | 0.534 | |  | 1.05 (0.95-1.16) | | 0.353 | | 0.0 | | 0.758 | |
| M00535: Isoleucine biosynthesis, pyruvate => 2-oxobutanoate | 1.19 (0.87-1.62) | 0.280 | 0.0 | 0.857 | |  | 1.14 (0.87-1.5) | | 0.345 | | 0.0 | | 0.946 | |
| M00448: CssS-CssR (secretion stress response) two-component regulatory system | 1.06 (0.95-1.18) | 0.285 | 0.0 | 0.640 | |  | 1.02 (0.87-1.2) | | 0.772 | | 62.6 | | 0.102 | |
| M00244: Putative zinc/manganese transport system | 1.13 (0.91-1.4) | 0.285 | 0.0 | 0.550 | |  | 1.12 (0.93-1.36) | | 0.221 | | 0.0 | | 0.343 | |
| M00348: Glutathione transport system | 0.96 (0.88-1.04) | 0.288 | 0.0 | 0.919 | |  | 0.93 (0.85-1.01) | | 0.099 | | 18.5 | | 0.268 | |
| M00306: PTS system, fructose-specific II-like component | 1.07 (0.94-1.22) | 0.291 | 0.0 | 0.948 | |  | 1.06 (0.94-1.19) | | 0.357 | | 0.0 | | 0.807 | |
| M00461: MtrB-MtrA (osmotic stress response) two-component regulatory system | 1.1 (0.92-1.32) | 0.295 | 0.0 | 0.791 | |  | 1.1 (0.93-1.29) | | 0.263 | | 0.0 | | 0.859 | |
| M00223: Phosphonate transport system | 0.95 (0.87-1.04) | 0.296 | 0.0 | 0.984 | |  | 0.97 (0.89-1.05) | | 0.477 | | 0.0 | | 0.670 | |
| M00259: Heme transport system | 0.92 (0.79-1.07) | 0.299 | 0.0 | 0.547 | |  | 0.93 (0.81-1.07) | | 0.301 | | 0.0 | | 0.711 | |
| M00324: Dipeptide transport system | 0.94 (0.85-1.05) | 0.302 | 0.0 | 0.574 | |  | 0.92 (0.81-1.06) | | 0.249 | | 39.5 | | 0.198 | |
| M00320: Lipopolysaccharide export system | 0.9 (0.75-1.09) | 0.302 | 42.9 | 0.186 | |  | 0.92 (0.81-1.03) | | 0.149 | | 0.0 | | 0.565 | |
| M00021: Cysteine biosynthesis, serine => cysteine | 1.18 (0.86-1.64) | 0.306 | 0.0 | 0.978 | |  | 1.11 (0.83-1.46) | | 0.485 | | 0.0 | | 0.658 | |
| M00455: TorS-TorR (TMAO respiration) two-component regulatory system | 0.94 (0.84-1.06) | 0.308 | 0.0 | 0.847 | |  | 0.96 (0.87-1.07) | | 0.452 | | 0.0 | | 0.484 | |
| M00144: NADH:quinone oxidoreductase, prokaryotes | 0.85 (0.63-1.16) | 0.310 | 0.0 | 0.417 | |  | 0.86 (0.65-1.13) | | 0.276 | | 0.0 | | 0.600 | |
| M00238: D-Methionine transport system | 1.17 (0.86-1.58) | 0.312 | 0.0 | 0.598 | |  | 1.16 (0.89-1.51) | | 0.283 | | 0.0 | | 0.367 | |
| M00116: Menaquinone biosynthesis, chorismate => menaquinone | 1.13 (0.89-1.42) | 0.319 | 0.0 | 0.346 | |  | 1.08 (0.88-1.32) | | 0.479 | | 0.0 | | 0.386 | |
| M00224: Fluoroquinolone transport system | 0.96 (0.89-1.04) | 0.326 | 0.0 | 0.643 | |  | 0.98 (0.92-1.05) | | 0.650 | | 0.0 | | 0.893 | |
| M00287: PTS system, galactosamine-specific II component | 1.07 (0.94-1.22) | 0.326 | 12.8 | 0.284 | |  | 1 (0.9-1.12) | | 0.958 | | 0.0 | | 0.787 | |
| M00529: Denitrification, nitrate => nitrogen | 0.92 (0.78-1.09) | 0.332 | 0.0 | 0.595 | |  | 0.96 (0.83-1.13) | | 0.649 | | 0.0 | | 0.469 | |
| M00478: DegS-DegU (multicellular behavior control) two-component regulatory system | 0.96 (0.87-1.05) | 0.332 | 0.0 | 0.954 | |  | 0.95 (0.87-1.03) | | 0.187 | | 0.0 | | 0.704 | |
| M00162: Cytochrome b6f complex | 1.08 (0.92-1.27) | 0.334 | 42.7 | 0.187 | |  | 1.09 (0.89-1.34) | | 0.406 | | 66.9 | | 0.082 | |
| M00193: Putative spermidine/putrescine transport system | 1.05 (0.95-1.16) | 0.335 | 0.0 | 0.761 | |  | 1.05 (0.96-1.16) | | 0.256 | | 0.0 | | 0.784 | |
| M00274: PTS system, mannitol-specific II component | 0.95 (0.85-1.06) | 0.337 | 0.0 | 0.813 | |  | 0.96 (0.87-1.05) | | 0.364 | | 0.0 | | 0.602 | |
| M00539: Cumate degradation, p-cumate => 2-oxopent-4-enoate + 2-methylpropanoate | 1.1 (0.9-1.34) | 0.351 | 75.5 | 0.043 | |  | 1.14 (0.94-1.38) | | 0.170 | | 77.0 | | 0.037 | |
| M00474: RcsC-RcsD-RcsB (capsule synthesis) two-component regulatory system | 0.96 (0.88-1.05) | 0.353 | 0.0 | 0.838 | |  | 0.96 (0.89-1.04) | | 0.291 | | 0.0 | | 0.816 | |
| M00114: Ascorbate biosynthesis, plants, glucose-6P => ascorbate | 1.15 (0.86-1.53) | 0.355 | 0.0 | 0.431 | |  | 1.07 (0.83-1.38) | | 0.614 | | 0.0 | | 0.599 | |
| M00155: Cytochrome c oxidase, prokaryotes | 0.88 (0.68-1.15) | 0.356 | 48.5 | 0.163 | |  | 0.93 (0.71-1.23) | | 0.624 | | 60.9 | | 0.110 | |
| M00198: Putative sn-glycerol-phosphate transport system | 0.95 (0.85-1.06) | 0.357 | 26.1 | 0.245 | |  | 0.98 (0.9-1.06) | | 0.619 | | 0.0 | | 0.547 | |
| M00505: KinB-AlgB (alginate production) two-component regulatory system | 1.04 (0.95-1.14) | 0.357 | 0.0 | 0.589 | |  | 1.05 (0.97-1.14) | | 0.226 | | 0.0 | | 0.795 | |
| M00124: Pyridoxal biosynthesis, erythrose-4P => pyridoxal-5P | 0.88 (0.67-1.16) | 0.366 | 0.0 | 0.358 | |  | 0.9 (0.66-1.23) | | 0.526 | | 38.7 | | 0.202 | |
| M00247: Putative ABC transport system | 1.11 (0.88-1.39) | 0.368 | 0.0 | 0.960 | |  | 1.13 (0.92-1.37) | | 0.247 | | 0.0 | | 0.853 | |
| M00450: BaeS-BaeR (envelope stress response) two-component regulatory system | 0.96 (0.89-1.05) | 0.371 | 0.0 | 0.842 | |  | 0.98 (0.9-1.05) | | 0.509 | | 0.0 | | 0.617 | |
| M00099: Sphingosine biosynthesis | 1.14 (0.86-1.52) | 0.373 | 69.1 | 0.072 | |  | 1.11 (0.88-1.41) | | 0.384 | | 65.2 | | 0.090 | |
| M00500: AtoS-AtoC (cPHB biosynthesis) two-component regulatory system | 0.95 (0.84-1.07) | 0.377 | 0.0 | 0.476 | |  | 0.9 (0.77-1.04) | | 0.150 | | 29.0 | | 0.235 | |
| M00082: Fatty acid biosynthesis, initiation | 1.18 (0.81-1.7) | 0.387 | 10.0 | 0.292 | |  | 1.13 (0.83-1.56) | | 0.436 | | 0.0 | | 0.442 | |
| M00118: Glutathione biosynthesis, glutamate => glutathione | 0.92 (0.77-1.1) | 0.388 | 0.0 | 0.682 | |  | 0.93 (0.79-1.09) | | 0.372 | | 0.0 | | 0.960 | |
| M00171: C4-dicarboxylic acid cycle, NAD - malic enzyme type | 1.14 (0.85-1.52) | 0.394 | 0.0 | 0.738 | |  | 1.1 (0.86-1.42) | | 0.440 | | 0.0 | | 0.726 | |
| M00034: Methionine salvage pathway | 0.81 (0.5-1.32) | 0.402 | 62.5 | 0.103 | |  | 0.78 (0.45-1.36) | | 0.383 | | 73.2 | | 0.053 | |
| M00089: Triacylglycerol biosynthesis | 1.16 (0.81-1.65) | 0.420 | 0.0 | 0.462 | |  | 1.16 (0.84-1.6) | | 0.366 | | 0.0 | | 0.495 | |
| M00098: Acylglycerol degradation | 1.04 (0.94-1.15) | 0.422 | 0.0 | 0.947 | |  | 0.98 (0.9-1.08) | | 0.750 | | 0.0 | | 0.890 | |
| M00314: Bacitracin transport system | 0.96 (0.86-1.06) | 0.424 | 0.0 | 0.857 | |  | 0.93 (0.77-1.12) | | 0.430 | | 56.7 | | 0.129 | |
| M00452: CusS-CusR (copper tolerance) two-component regulatory system | 0.95 (0.82-1.09) | 0.432 | 41.0 | 0.193 | |  | 0.99 (0.82-1.2) | | 0.940 | | 66.2 | | 0.085 | |
| M00548: Benzene degradation, benzene => catechol | 0.97 (0.89-1.05) | 0.435 | 0.0 | 0.645 | |  | 0.97 (0.9-1.04) | | 0.353 | | 0.0 | | 0.625 | |
| M00207: Putative multiple sugar transport system | 1.17 (0.79-1.72) | 0.436 | 43.1 | 0.185 | |  | 1.09 (0.84-1.4) | | 0.527 | | 0.0 | | 0.437 | |
| M00432: Leucine biosynthesis, 2-oxoisovalerate => 2-oxoisocaproate | 1.13 (0.82-1.56) | 0.439 | 0.0 | 0.393 | |  | 1.08 (0.81-1.43) | | 0.595 | | 0.0 | | 0.444 | |
| M00267: PTS system, N-acetylglucosamine-specific II component | 1.06 (0.91-1.24) | 0.439 | 22.2 | 0.257 | |  | 1.04 (0.92-1.18) | | 0.544 | | 0.0 | | 0.492 | |
| M00271: PTS system, beta-glucoside-specific II component | 1.05 (0.92-1.21) | 0.439 | 12.5 | 0.285 | |  | 1.03 (0.92-1.16) | | 0.609 | | 0.0 | | 0.428 | |
| M00499: HydH-HydG (metal tolerance) two-component regulatory system | 1.05 (0.93-1.18) | 0.460 | 0.0 | 0.409 | |  | 1.01 (0.91-1.13) | | 0.798 | | 0.0 | | 0.400 | |
| M00565: Trehalose biosynthesis, D-glucose 1P => trehalose | 1.1 (0.86-1.4) | 0.460 | 0.0 | 0.873 | |  | 1.11 (0.9-1.37) | | 0.340 | | 0.0 | | 0.897 | |
| M00052: Pyrimidine ribonucleotide biosynthesis, UMP => UDP/UTP,CDP/CTP | 1.12 (0.83-1.52) | 0.460 | 0.0 | 0.347 | |  | 1.06 (0.8-1.41) | | 0.662 | | 2.7 | | 0.311 | |
| M00027: GABA (gamma-Aminobutyrate) shunt | 1.08 (0.88-1.34) | 0.461 | 0.0 | 0.368 | |  | 1.07 (0.89-1.29) | | 0.455 | | 0.0 | | 0.468 | |
| M00236: Putative polar amino acid transport system | 1.14 (0.79-1.64) | 0.475 | 21.0 | 0.261 | |  | 1.02 (0.67-1.57) | | 0.919 | | 48.2 | | 0.165 | |
| M00104: Bile acid biosynthesis, cholesterol => cholate/chenodeoxycholate | 0.96 (0.86-1.07) | 0.479 | 0.0 | 0.649 | |  | 0.97 (0.88-1.08) | | 0.628 | | 0.0 | | 0.458 | |
| M00208: Glycine betaine/proline transport system | 1.05 (0.92-1.21) | 0.483 | 0.0 | 0.899 | |  | 1.06 (0.95-1.2) | | 0.306 | | 0.0 | | 0.976 | |
| M00334: Type VI secretion system | 0.95 (0.82-1.1) | 0.484 | 0.0 | 0.831 | |  | 1 (0.87-1.15) | | 0.985 | | 0.0 | | 0.985 | |
| M00170: C4-dicarboxylic acid cycle, phosphoenolpyruvate carboxykinase type | 0.93 (0.76-1.14) | 0.487 | 22.7 | 0.255 | |  | 0.96 (0.76-1.21) | | 0.722 | | 51.6 | | 0.151 | |
| M00489: DctS-DctR (C4-dicarboxylate transport) two-component regulatory system | 1.09 (0.86-1.37) | 0.493 | 64.5 | 0.093 | |  | 0.99 (0.85-1.16) | | 0.927 | | 30.7 | | 0.230 | |
| M00081: Pectin degradation | 0.95 (0.84-1.09) | 0.498 | 10.4 | 0.291 | |  | 0.95 (0.73-1.23) | | 0.680 | | 75.1 | | 0.045 | |
| M00255: Lipoprotein-releasing system | 0.92 (0.72-1.18) | 0.500 | 65.6 | 0.088 | |  | 0.92 (0.76-1.1) | | 0.355 | | 49.0 | | 0.161 | |
| M00175: Nitrogen fixation, nitrogen => ammonia | 1.05 (0.92-1.19) | 0.500 | 0.0 | 0.529 | |  | 1.07 (0.93-1.22) | | 0.350 | | 20.6 | | 0.262 | |
| M00473: UhpB-UhpA (hexose phosphates uptake) two-component regulatory system | 0.94 (0.78-1.13) | 0.502 | 22.3 | 0.257 | |  | 0.94 (0.81-1.09) | | 0.427 | | 1.8 | | 0.313 | |
| M00167: Reductive pentose phosphate cycle, glyceraldehyde-3P => ribulose-5P | 1.11 (0.82-1.51) | 0.503 | 0.0 | 0.659 | |  | 1.07 (0.81-1.4) | | 0.648 | | 0.0 | | 0.596 | |
| M00375: Hydroxypropionate-hydroxybutylate cycle | 0.9 (0.65-1.23) | 0.509 | 0.0 | 0.571 | |  | 0.96 (0.7-1.32) | | 0.800 | | 20.6 | | 0.262 | |
| M00268: PTS system, alpha-glucoside-specific II component | 1.05 (0.92-1.2) | 0.510 | 0.0 | 0.760 | |  | 1.02 (0.91-1.15) | | 0.714 | | 0.0 | | 0.769 | |
| M00435: Taurine transport system | 0.97 (0.89-1.06) | 0.512 | 0.0 | 0.924 | |  | 0.97 (0.89-1.05) | | 0.410 | | 0.0 | | 0.932 | |
| M00525: Lysine biosynthesis, acetyl-DAP pathway, aspartate => lysine | 1.12 (0.8-1.56) | 0.512 | 0.0 | 0.428 | |  | 1.05 (0.78-1.4) | | 0.757 | | 0.0 | | 0.423 | |
| M00376: 3-Hydroxypropionate bi-cycle | 0.89 (0.64-1.26) | 0.518 | 0.0 | 0.712 | |  | 0.9 (0.66-1.21) | | 0.480 | | 0.0 | | 0.389 | |
| M00555: Betaine biosynthesis, choline => betaine | 0.95 (0.83-1.1) | 0.519 | 0.0 | 0.777 | |  | 1 (0.88-1.14) | | 0.972 | | 0.0 | | 0.762 | |
| M00495: AgrC-AgrA (exoprotein synthesis) two-component regulatory system | 1.05 (0.91-1.21) | 0.523 | 0.0 | 0.366 | |  | 1.02 (0.84-1.23) | | 0.877 | | 47.2 | | 0.169 | |
| M00239: Peptides/nickel transport system | 1.1 (0.82-1.49) | 0.525 | 15.6 | 0.276 | |  | 1.06 (0.84-1.36) | | 0.614 | | 3.2 | | 0.309 | |
| M00447: CpxA-CpxR (envelope stress response) two-component regulatory system | 0.97 (0.88-1.07) | 0.528 | 0.0 | 0.690 | |  | 0.94 (0.86-1.03) | | 0.197 | | 0.0 | | 0.324 | |
| M00165: Reductive pentose phosphate cycle (Calvin cycle) | 1.1 (0.81-1.49) | 0.531 | 0.0 | 0.744 | |  | 1.08 (0.82-1.41) | | 0.595 | | 0.0 | | 0.614 | |
| M00362: Nucleotide sugar biosynthesis, prokaryotes | 1.11 (0.81-1.51) | 0.531 | 0.0 | 0.659 | |  | 1.06 (0.8-1.4) | | 0.688 | | 0.0 | | 0.874 | |
| M00491: arabinogalactan oligomer/maltooligosaccharide transport system | 1.09 (0.84-1.42) | 0.532 | 0.0 | 0.555 | |  | 1.01 (0.79-1.29) | | 0.925 | | 0.0 | | 0.780 | |
| M00530: Dissimilatory nitrate reduction, nitrate => ammonia | 0.94 (0.79-1.13) | 0.533 | 0.0 | 0.854 | |  | 0.93 (0.8-1.09) | | 0.379 | | 0.0 | | 0.818 | |
| M00127: Thiamine biosynthesis, AIR => thiamine-P/thiamine-2P | 1.1 (0.82-1.48) | 0.536 | 0.0 | 0.884 | |  | 1.12 (0.86-1.46) | | 0.391 | | 0.0 | | 0.995 | |
| M00049: Adenine ribonucleotide biosynthesis, IMP => ADP,ATP | 1.1 (0.81-1.49) | 0.538 | 0.0 | 0.738 | |  | 1.03 (0.79-1.35) | | 0.821 | | 0.0 | | 0.823 | |
| M00216: Multiple sugar transport system | 1.06 (0.89-1.26) | 0.540 | 0.0 | 0.610 | |  | 1.02 (0.86-1.19) | | 0.849 | | 0.0 | | 0.654 | |
| M00277: PTS system, N-acetylgalactosamine-specific II component | 1.06 (0.89-1.26) | 0.541 | 48.1 | 0.165 | |  | 1 (0.89-1.12) | | 0.977 | | 0.0 | | 0.429 | |
| M00007: Pentose phosphate pathway, non-oxidative phase, fructose 6P => ribose 5P | 1.1 (0.81-1.49) | 0.541 | 0.0 | 0.422 | |  | 1.02 (0.78-1.34) | | 0.868 | | 0.0 | | 0.489 | |
| M00215: D-Xylose transport system | 1.04 (0.91-1.2) | 0.545 | 0.0 | 0.734 | |  | 1.03 (0.91-1.17) | | 0.628 | | 0.0 | | 0.674 | |
| M00481: LiaS-LiaR (cell wall stress response) two-component regulatory system | 0.95 (0.8-1.13) | 0.546 | 69.7 | 0.069 | |  | 0.98 (0.89-1.08) | | 0.689 | | 38.7 | | 0.201 | |
| M00509: WspE-WspRF (chemosensory) two-component regulatory system | 1.04 (0.92-1.17) | 0.558 | 31.9 | 0.226 | |  | 1.02 (0.94-1.1) | | 0.666 | | 0.0 | | 0.333 | |
| M00305: PTS system, 2-O-A-mannosyl-D-glycerate-specific II component | 0.96 (0.85-1.09) | 0.558 | 0.0 | 0.765 | |  | **0.89 (0.8-1)** | | **0.046** | | 0.0 | | 0.497 | |
| M00050: Guanine ribonucleotide biosynthesis IMP => GDP,GTP | 1.09 (0.82-1.45) | 0.558 | 0.0 | 0.816 | |  | 1.04 (0.81-1.35) | | 0.755 | | 0.0 | | 0.810 | |
| M00527: Lysine biosynthesis, DAP aminotransferase pathway, aspartate => lysine | 1.1 (0.79-1.53) | 0.565 | 0.0 | 0.530 | |  | 1.04 (0.78-1.39) | | 0.781 | | 0.0 | | 0.514 | |
| M00016: Lysine biosynthesis, succinyl-DAP pathway, aspartate => lysine | 1.1 (0.79-1.53) | 0.565 | 0.0 | 0.642 | |  | 1.04 (0.78-1.38) | | 0.799 | | 0.0 | | 0.636 | |
| M00115: NAD biosynthesis, aspartate => NAD | 1.09 (0.82-1.44) | 0.568 | 0.0 | 0.925 | |  | 1.08 (0.84-1.38) | | 0.569 | | 0.0 | | 0.934 | |
| M00004: Pentose phosphate pathway (Pentose phosphate cycle) | 1.09 (0.82-1.45) | 0.568 | 0.0 | 0.403 | |  | 1 (0.77-1.29) | | 1.000 | | 0.0 | | 0.550 | |
| M00546: Purine degradation, xanthine => urea | 0.96 (0.84-1.1) | 0.570 | 0.6 | 0.316 | |  | 1.01 (0.87-1.17) | | 0.905 | | 16.7 | | 0.273 | |
| M00001: Glycolysis (Embden-Meyerhof pathway), glucose => pyruvate | 1.09 (0.8-1.48) | 0.574 | 0.0 | 0.511 | |  | 1.03 (0.79-1.36) | | 0.816 | | 0.0 | | 0.521 | |
| M00518: GlnK-GlnL (glutamine utilization) two-component regulatory system | 0.96 (0.83-1.11) | 0.577 | 45.3 | 0.176 | |  | 0.96 (0.89-1.04) | | 0.289 | | 0.0 | | 0.536 | |
| M00567: Methanogenesis, CO2 => methane | 0.98 (0.91-1.05) | 0.582 | 0.0 | 0.877 | |  | 1.04 (0.97-1.12) | | 0.225 | | 0.0 | | 0.702 | |
| M00141: C1-unit interconversion, eukaryotes | 1.08 (0.81-1.45) | 0.582 | 0.0 | 0.441 | |  | 0.99 (0.76-1.29) | | 0.959 | | 0.0 | | 0.664 | |
| M00429: Competence-related DNA transformation transporter | 1.08 (0.82-1.43) | 0.583 | 0.0 | 0.372 | |  | 1.04 (0.81-1.33) | | 0.743 | | 0.0 | | 0.525 | |
| M00038: Tryptophan metabolism, tryptophan => kynurenine => 2-aminomuconate | 0.93 (0.7-1.22) | 0.590 | 51.0 | 0.153 | |  | 0.98 (0.72-1.32) | | 0.873 | | 63.3 | | 0.099 | |
| M00020: Serine biosynthesis, glycerate-3P => serine | 1.08 (0.81-1.44) | 0.593 | 0.0 | 0.419 | |  | 1.02 (0.79-1.31) | | 0.874 | | 0.0 | | 0.467 | |
| M00303: PTS system, N-acetylmuramic acid-specific II component | 1.04 (0.91-1.18) | 0.593 | 0.0 | 0.919 | |  | 1.01 (0.9-1.14) | | 0.852 | | 0.0 | | 0.569 | |
| M00265: PTS system, glucose-specific II component | 1.04 (0.91-1.19) | 0.596 | 0.0 | 0.859 | |  | 1 (0.89-1.13) | | 0.947 | | 0.0 | | 0.655 | |
| M00330: Adhesin protein transport system | 0.97 (0.86-1.09) | 0.597 | 21.0 | 0.260 | |  | 0.98 (0.9-1.07) | | 0.627 | | 0.0 | | 0.343 | |
| M00159: V/A-type ATPase, prokaryotes | 0.94 (0.74-1.19) | 0.600 | 81.5 | 0.020 | |  | 0.99 (0.8-1.22) | | 0.919 | | 80.0 | | 0.025 | |
| M00192: Putative thiamine transport system | 0.97 (0.88-1.08) | 0.600 | 0.0 | 0.908 | |  | 0.98 (0.9-1.07) | | 0.632 | | 0.0 | | 0.777 | |
| M00241: Vitamin B12 transport system | 0.96 (0.84-1.11) | 0.602 | 50.5 | 0.155 | |  | 0.95 (0.84-1.07) | | 0.407 | | 47.9 | | 0.166 | |
| M00218: Fructose transport system | 1.03 (0.92-1.15) | 0.606 | 44.3 | 0.180 | |  | 1.09 (0.98-1.21) | | 0.118 | | 45.1 | | 0.177 | |
| M00423: Molybdate/tungstate transport system | 1.03 (0.92-1.15) | 0.608 | 0.0 | 0.798 | |  | 1.08 (0.97-1.2) | | 0.149 | | 0.0 | | 0.522 | |
| M00079: Keratan sulfate degradation | 1.05 (0.88-1.25) | 0.609 | 0.0 | 0.589 | |  | 0.97 (0.83-1.13) | | 0.712 | | 0.0 | | 0.401 | |
| M00163: Photosystem I | 1.06 (0.85-1.31) | 0.613 | 75.1 | 0.045 | |  | 1.03 (0.77-1.38) | | 0.825 | | 87.5 | | 0.005 | |
| M00006: Pentose phosphate pathway, oxidative phase, glucose 6P => ribulose 5P | 1.06 (0.84-1.36) | 0.614 | 0.0 | 0.368 | |  | 0.98 (0.79-1.22) | | 0.872 | | 0.0 | | 0.671 | |
| M00457: TctE-TctD (tricarboxylic acid transport) two-component regulatory system | 0.98 (0.88-1.08) | 0.618 | 0.0 | 0.338 | |  | 1.04 (0.85-1.27) | | 0.680 | | 61.5 | | 0.107 | |
| M00036: Leucine degradation, leucine => acetoacetate + acetyl-CoA | 0.87 (0.51-1.48) | 0.619 | 48.4 | 0.164 | |  | 0.9 (0.52-1.58) | | 0.720 | | 65.3 | | 0.090 | |
| M00242: Zinc transport system | 1.04 (0.88-1.24) | 0.620 | 0.0 | 0.669 | |  | 1.01 (0.87-1.18) | | 0.911 | | 0.0 | | 0.698 | |
| M00272: PTS system, beta-glucoside (arbutin/salicin/cellobiose)-specific II component | 1.03 (0.9-1.18) | 0.624 | 0.0 | 0.924 | |  | 1.01 (0.89-1.13) | | 0.920 | | 0.0 | | 0.582 | |
| M00005: PRPP biosynthesis, ribose 5P => PRPP | 0.91 (0.63-1.32) | 0.625 | 26.5 | 0.243 | |  | 0.88 (0.61-1.25) | | 0.468 | | 38.2 | | 0.203 | |
| M00228: Putative glutamine transport system | 0.95 (0.78-1.16) | 0.630 | 75.6 | 0.043 | |  | 1 (0.93-1.08) | | 0.998 | | 11.1 | | 0.289 | |
| M00076: Dermatan sulfate degradation | 0.97 (0.87-1.09) | 0.631 | 0.0 | 0.851 | |  | 0.94 (0.86-1.04) | | 0.247 | | 0.0 | | 0.570 | |
| M00575: Pertussis pathogenicity signature 2, T1SS | 0.94 (0.73-1.21) | 0.631 | 58.4 | 0.121 | |  | 0.95 (0.82-1.1) | | 0.523 | | 13.5 | | 0.282 | |
| M00331: Type II general secretion pathway | 0.94 (0.75-1.2) | 0.640 | 56.2 | 0.131 | |  | 0.96 (0.76-1.2) | | 0.712 | | 57.5 | | 0.125 | |
| M00526: Lysine biosynthesis, DAP dehydrogenase pathway, aspartate => lysine | 1.08 (0.78-1.49) | 0.643 | 0.0 | 0.474 | |  | 1.03 (0.78-1.37) | | 0.834 | | 0.0 | | 0.427 | |
| M00300: Putrescine transport system | 0.97 (0.84-1.11) | 0.643 | 36.9 | 0.208 | |  | 0.98 (0.89-1.08) | | 0.644 | | 0.0 | | 0.528 | |
| M00222: Phosphate transport system | 1.08 (0.79-1.46) | 0.643 | 0.0 | 0.357 | |  | 1.02 (0.78-1.34) | | 0.878 | | 0.0 | | 0.389 | |
| M00270: PTS system, trehalose-specific II component | 1.03 (0.91-1.17) | 0.648 | 0.0 | 0.720 | |  | 1 (0.9-1.12) | | 0.950 | | 0.0 | | 0.780 | |
| M00568: Catechol ortho-cleavage, catechol => 3-oxoadipate | 0.98 (0.9-1.06) | 0.649 | 0.0 | 0.572 | |  | 1 (0.92-1.07) | | 0.920 | | 0.0 | | 0.965 | |
| M00439: Oligopeptide transport system | 1.09 (0.76-1.56) | 0.650 | 66.2 | 0.086 | |  | 1.01 (0.8-1.27) | | 0.957 | | 32.4 | | 0.224 | |
| M00221: Putative simple sugar transport system | 1.08 (0.77-1.51) | 0.651 | 14.7 | 0.279 | |  | 1.02 (0.78-1.33) | | 0.898 | | 0.0 | | 0.519 | |
| M00532: Photorespiration | 1.08 (0.77-1.51) | 0.651 | 0.0 | 0.844 | |  | 1.08 (0.8-1.45) | | 0.625 | | 0.0 | | 0.624 | |
| M00316: Manganese transport system | 0.98 (0.9-1.06) | 0.653 | 0.0 | 0.974 | |  | 0.98 (0.86-1.11) | | 0.714 | | 50.8 | | 0.154 | |
| M00096: C5 isoprenoid biosynthesis, non-mevalonate pathway | 1.07 (0.79-1.45) | 0.655 | 0.0 | 0.404 | |  | 1.02 (0.78-1.33) | | 0.882 | | 0.0 | | 0.435 | |
| M00269: PTS system, sucrose-specific II component | 1.03 (0.91-1.16) | 0.659 | 0.0 | 0.403 | |  | 0.98 (0.88-1.1) | | 0.795 | | 0.0 | | 0.897 | |
| M00357: Methanogenesis, acetate => methane | 1.06 (0.81-1.4) | 0.661 | 0.0 | 0.894 | |  | 1.05 (0.82-1.34) | | 0.705 | | 0.0 | | 0.945 | |
| M00032: Lysine degradation, lysine => saccharopine => acetoacetyl-CoA | 1.05 (0.84-1.31) | 0.663 | 0.0 | 0.540 | |  | 1.05 (0.86-1.28) | | 0.625 | | 0.0 | | 0.671 | |
| M00030: Lysine biosynthesis, AAA pathway, 2-oxoglutarate => 2-aminoadipate => lysine | 1.04 (0.86-1.26) | 0.664 | 2.7 | 0.311 | |  | 1.07 (0.76-1.5) | | 0.715 | | 71.3 | | 0.062 | |
| M00194: Maltose/maltodextrin transport system | 1.06 (0.82-1.36) | 0.670 | 0.0 | 0.349 | |  | 1 (0.8-1.26) | | 0.981 | | 0.0 | | 0.411 | |
| M00166: Reductive pentose phosphate cycle, ribulose-5P => glyceraldehyde-3P | 1.06 (0.8-1.4) | 0.671 | 0.0 | 0.955 | |  | 1.04 (0.81-1.33) | | 0.777 | | 0.0 | | 0.878 | |
| M00504: DctB-DctD (C4-dicarboxylate transport) two-component regulatory system | 1.04 (0.87-1.24) | 0.672 | 37.1 | 0.207 | |  | 0.99 (0.9-1.1) | | 0.906 | | 0.0 | | 0.321 | |
| M00129: Ascorbate biosynthesis, animals, glucose-1P => ascorbate | 1.05 (0.83-1.33) | 0.673 | 0.0 | 0.783 | |  | 1.06 (0.86-1.3) | | 0.570 | | 0.0 | | 0.872 | |
| M00345: Formaldehyde assimilation, ribulose monophosphate pathway | 1.07 (0.77-1.49) | 0.675 | 9.4 | 0.294 | |  | 1.02 (0.76-1.37) | | 0.897 | | 13.9 | | 0.281 | |
| M00477: EvgS-EvgA (acid and drug tolerance) two-component regulatory system | 0.98 (0.9-1.07) | 0.677 | 0.0 | 0.957 | |  | 0.98 (0.91-1.06) | | 0.635 | | 0.0 | | 0.761 | |
| M00204: Trehalose/maltose transport system | 0.98 (0.9-1.07) | 0.677 | 0.0 | 0.710 | |  | 0.97 (0.87-1.08) | | 0.588 | | 33.3 | | 0.221 | |
| M00012: Glyoxylate cycle | 1.11 (0.68-1.82) | 0.678 | 57.8 | 0.124 | |  | 1.06 (0.72-1.58) | | 0.756 | | 43.0 | | 0.185 | |
| M00126: Tetrahydrofolate biosynthesis, GTP => THF | 1.06 (0.81-1.39) | 0.680 | 0.0 | 0.465 | |  | 1 (0.79-1.28) | | 0.978 | | 0.0 | | 0.590 | |
| M00257: Hemin transport system | 0.98 (0.87-1.09) | 0.681 | 0.0 | 0.755 | |  | 0.95 (0.76-1.18) | | 0.648 | | 67.6 | | 0.079 | |
| M00190: Iron(III) transport system | 0.97 (0.82-1.14) | 0.682 | 0.0 | 0.454 | |  | 1.03 (0.88-1.19) | | 0.749 | | 0.0 | | 0.768 | |
| M00123: Biotin biosynthesis, pimeloyl-ACP/CoA => biotin | 1.08 (0.76-1.53) | 0.684 | 0.0 | 0.823 | |  | 1.06 (0.78-1.45) | | 0.708 | | 0.0 | | 0.936 | |
| M00359: Aminoacyl-tRNA biosynthesis, eukaryotes | 1.06 (0.79-1.43) | 0.689 | 0.0 | 0.415 | |  | 1.03 (0.79-1.33) | | 0.826 | | 0.0 | | 0.438 | |
| M00273: PTS system, fructose-specific II component | 1.05 (0.82-1.36) | 0.691 | 53.2 | 0.144 | |  | 1.02 (0.81-1.28) | | 0.878 | | 50.6 | | 0.155 | |
| M00325: alpha-Hemolysin/cyclolysin transport system | 0.95 (0.72-1.24) | 0.693 | 62.8 | 0.101 | |  | 0.96 (0.81-1.14) | | 0.649 | | 28.0 | | 0.239 | |
| M00225: Lysine/arginine/ornithine transport system | 1.02 (0.93-1.11) | 0.693 | 0.0 | 0.893 | |  | 1.03 (0.95-1.11) | | 0.461 | | 0.0 | | 0.615 | |
| M00438: Nitrate/nitrite transport system | 0.98 (0.88-1.08) | 0.695 | 0.0 | 0.576 | |  | 0.98 (0.9-1.08) | | 0.717 | | 0.0 | | 0.396 | |
| M00483: NreB-NreC (dissimilatory nitrate/nitrite reduction) two-component regulatory system | 1.02 (0.92-1.13) | 0.698 | 0.0 | 0.681 | |  | 1.03 (0.93-1.13) | | 0.567 | | 0.0 | | 0.602 | |
| M00097: beta-Carotene biosynthesis, GGAP => beta-carotene | 1.03 (0.88-1.21) | 0.700 | 0.0 | 0.719 | |  | 1.06 (0.92-1.22) | | 0.427 | | 0.0 | | 0.521 | |
| M00173: Reductive citrate cycle (Arnon-Buchanan cycle) | 1.07 (0.76-1.51) | 0.704 | 0.0 | 0.485 | |  | 0.98 (0.72-1.33) | | 0.889 | | 0.0 | | 0.552 | |
| M00358: Coenzyme M biosynthesis | 1.04 (0.85-1.28) | 0.708 | 52.7 | 0.146 | |  | 1.05 (0.79-1.4) | | 0.726 | | 78.4 | | 0.031 | |
| M00497: GlnL-GlnG (nitrogen regulation) two-component regulatory system | 0.94 (0.66-1.33) | 0.714 | 77.1 | 0.037 | |  | 0.94 (0.7-1.26) | | 0.687 | | 72.0 | | 0.059 | |
| M00531: Assimilatory nitrate reduction, nitrate => ammonia | 1.06 (0.77-1.48) | 0.716 | 52.6 | 0.147 | |  | 1.1 (0.76-1.6) | | 0.598 | | 65.2 | | 0.090 | |
| M00570: Isoleucine biosynthesis, threonine => 2-oxobutanoate => isoleucine | 1.06 (0.76-1.49) | 0.716 | 0.0 | 0.465 | |  | 1.01 (0.75-1.35) | | 0.941 | | 0.0 | | 0.434 | |
| M00024: Phenylalanine biosynthesis, chorismate => phenylalanine | 1.06 (0.78-1.43) | 0.719 | 0.0 | 0.436 | |  | 1 (0.76-1.31) | | 0.999 | | 0.0 | | 0.422 | |
| M00131: Inositol phosphate metabolism, Ins(1,3,4,5)P4 => Ins(1,3,4)P3 => myo-inositol | 0.96 (0.77-1.2) | 0.720 | 0.0 | 0.589 | |  | 0.98 (0.8-1.19) | | 0.812 | | 0.0 | | 0.421 | |
| M00266: PTS system, maltose/glucose-specific II component | 1.03 (0.89-1.18) | 0.721 | 0.0 | 0.671 | |  | 0.99 (0.87-1.12) | | 0.849 | | 0.0 | | 0.937 | |
| M00008: Entner-Doudoroff pathway, glucose-6P => glyceraldehyde-3P + pyruvate | 1.05 (0.79-1.4) | 0.722 | 0.0 | 0.421 | |  | 0.97 (0.75-1.25) | | 0.817 | | 0.0 | | 0.616 | |
| M00485: KinABCDE-Spo0FA (sporulation control) two-component regulatory system | 0.98 (0.9-1.08) | 0.725 | 0.0 | 0.636 | |  | 0.98 (0.89-1.08) | | 0.728 | | 26.0 | | 0.245 | |
| M00077: Chondroitin sulfate degradation | 0.98 (0.88-1.09) | 0.727 | 0.0 | 0.801 | |  | 0.95 (0.87-1.05) | | 0.319 | | 0.0 | | 0.625 | |
| M00307: Pyruvate oxidation, pyruvate => acetyl-CoA | 1.06 (0.75-1.5) | 0.727 | 0.0 | 0.410 | |  | 1.03 (0.76-1.39) | | 0.841 | | 0.0 | | 0.413 | |
| M00022: Shikimate pathway, phosphoenolpyruvate + erythrose-4P => chorismate | 1.06 (0.77-1.45) | 0.728 | 0.0 | 0.353 | |  | 1.02 (0.77-1.34) | | 0.903 | | 0.0 | | 0.352 | |
| M00361: Nucleotide sugar biosynthesis, eukaryotes | 1.05 (0.78-1.41) | 0.732 | 0.0 | 0.865 | |  | 1.02 (0.79-1.33) | | 0.880 | | 0.0 | | 0.936 | |
| M00258: Putative ABC transport system | 1.05 (0.79-1.39) | 0.734 | 0.0 | 0.341 | |  | 1 (0.78-1.28) | | 0.993 | | 0.0 | | 0.525 | |
| M00013: Malonate semialdehyde pathway, propanoyl-CoA => acetyl-CoA | 0.97 (0.79-1.19) | 0.735 | 0.0 | 0.987 | |  | 0.98 (0.81-1.18) | | 0.820 | | 0.0 | | 0.785 | |
| M00011: Citrate cycle, second carbon oxidation, 2-oxoglutarate => oxaloacetate | 1.06 (0.76-1.48) | 0.736 | 0.0 | 0.727 | |  | 0.98 (0.73-1.32) | | 0.891 | | 0.0 | | 0.713 | |
| M00540: Benzoate degradation, cyclohexanecarboxylic acid =>pimeloyl-CoA | 1.02 (0.93-1.11) | 0.739 | 0.0 | 0.893 | |  | 1.01 (0.93-1.09) | | 0.870 | | 0.0 | | 0.537 | |
| M00186: Tungstate transport system | 0.97 (0.82-1.15) | 0.742 | 53.4 | 0.143 | |  | 0.99 (0.77-1.28) | | 0.967 | | 80.6 | | 0.023 | |
| M00149: Succinate dehydrogenase, prokaryotes | 1.04 (0.81-1.36) | 0.742 | 0.0 | 0.798 | |  | 1.01 (0.8-1.27) | | 0.916 | | 0.0 | | 0.640 | |
| M00003: Gluconeogenesis, oxaloacetate => fructose-6P | 1.05 (0.79-1.4) | 0.743 | 0.0 | 0.518 | |  | 1.01 (0.78-1.3) | | 0.950 | | 0.0 | | 0.460 | |
| M00168: CAM (Crassulacean acid metabolism), dark | 1.04 (0.81-1.34) | 0.747 | 0.0 | 0.991 | |  | 1.06 (0.85-1.31) | | 0.625 | | 0.0 | | 0.828 | |
| M00550: Ascorbate degradation, ascorbate => D-xylulose-5P | 0.98 (0.86-1.11) | 0.747 | 0.0 | 0.646 | |  | 1.01 (0.9-1.13) | | 0.889 | | 0.0 | | 0.989 | |
| M00538: Toluene degradation, toluene => benzoate | 1.03 (0.87-1.22) | 0.748 | 0.0 | 0.594 | |  | 1.01 (0.87-1.17) | | 0.899 | | 0.0 | | 0.735 | |
| M00169: CAM (Crassulacean acid metabolism), light | 1.07 (0.7-1.65) | 0.751 | 57.3 | 0.126 | |  | 1.1 (0.58-2.08) | | 0.769 | | 83.5 | | 0.014 | |
| M00154: Cytochrome c oxidase | 1.04 (0.81-1.33) | 0.754 | 26.7 | 0.243 | |  | 1.04 (0.81-1.34) | | 0.738 | | 44.8 | | 0.178 | |
| M00053: Pyrimidine deoxyribonuleotide biosynthesis, CDP/CTP => dCDP/dCTP,dTDP/dTTP | 1.05 (0.77-1.42) | 0.756 | 0.0 | 0.475 | |  | 1 (0.77-1.31) | | 0.975 | | 0.0 | | 0.473 | |
| M00254: ABC-2 type transport system | 1.05 (0.77-1.43) | 0.757 | 19.5 | 0.265 | |  | 0.99 (0.77-1.26) | | 0.905 | | 0.0 | | 0.400 | |
| M00087: beta-Oxidation | 0.96 (0.75-1.23) | 0.761 | 0.0 | 0.646 | |  | 0.97 (0.78-1.2) | | 0.766 | | 0.0 | | 0.741 | |
| M00161: Photosystem II | 1.04 (0.81-1.34) | 0.766 | 83.3 | 0.014 | |  | 1.04 (0.77-1.39) | | 0.815 | | 88.0 | | 0.004 | |
| M00511: PleC-PleD (cell fate control) two-component regulatory system | 1.03 (0.85-1.24) | 0.772 | 59.1 | 0.118 | |  | 1 (0.85-1.18) | | 0.983 | | 54.9 | | 0.136 | |
| M00344: Formaldehyde assimilation, xylulose monophosphate pathway | 1.05 (0.74-1.49) | 0.774 | 0.0 | 0.681 | |  | 1 (0.74-1.37) | | 0.989 | | 0.0 | | 0.673 | |
| M00360: Aminoacyl-tRNA biosynthesis, prokaryotes | 1.04 (0.77-1.41) | 0.777 | 0.0 | 0.439 | |  | 1.02 (0.78-1.32) | | 0.912 | | 0.0 | | 0.452 | |
| M00378: F420 biosynthesis | 0.98 (0.83-1.14) | 0.779 | 0.0 | 0.821 | |  | 1.01 (0.88-1.16) | | 0.918 | | 0.0 | | 0.579 | |
| M00174: Methane oxidation, methanotroph, methane => formaldehyde | 0.96 (0.73-1.27) | 0.779 | 0.0 | 0.330 | |  | 1 (0.79-1.27) | | 0.998 | | 0.0 | | 0.550 | |
| M00014: Glucuronate pathway (uronate pathway) | 1.04 (0.77-1.41) | 0.780 | 34.2 | 0.218 | |  | 1.09 (0.82-1.47) | | 0.546 | | 44.9 | | 0.178 | |
| M00368: Ethylene biosynthesis, methionine => ethylene | 0.95 (0.65-1.39) | 0.784 | 66.4 | 0.085 | |  | 0.9 (0.6-1.35) | | 0.601 | | 73.3 | | 0.053 | |
| M00176: Assimilatory sulfate reduction, sulfate => H2S | 1.03 (0.81-1.32) | 0.785 | 0.0 | 0.696 | |  | 1.07 (0.86-1.33) | | 0.558 | | 0.0 | | 0.803 | |
| M00227: Glutamine transport system | 1.03 (0.84-1.26) | 0.788 | 76.9 | 0.038 | |  | 0.98 (0.8-1.21) | | 0.875 | | 82.5 | | 0.017 | |
| M00183: RNA polymerase, bacteria | 1.04 (0.77-1.4) | 0.788 | 0.0 | 0.477 | |  | 0.97 (0.74-1.26) | | 0.795 | | 0.0 | | 0.555 | |
| M00472: NarQ-NarP (nitrate respiration) two-component regulatory system | 0.97 (0.77-1.22) | 0.789 | 77.9 | 0.033 | |  | 0.94 (0.74-1.18) | | 0.589 | | 80.2 | | 0.025 | |
| M00317: Manganese/iron transport system | 0.99 (0.9-1.09) | 0.792 | 0.0 | 0.949 | |  | 0.99 (0.91-1.08) | | 0.899 | | 0.0 | | 0.751 | |
| M00134: Polyamine biosynthesis, arginine => ornithine => putrescine | 0.98 (0.88-1.11) | 0.792 | 10.4 | 0.291 | |  | 0.98 (0.89-1.08) | | 0.658 | | 0.0 | | 0.894 | |
| M00083: Fatty acid biosynthesis, elongation | 1.05 (0.71-1.56) | 0.797 | 0.0 | 0.957 | |  | 0.97 (0.68-1.38) | | 0.867 | | 0.0 | | 0.973 | |
| M00436: Sulfonate transport system | 0.98 (0.83-1.16) | 0.797 | 54.9 | 0.137 | |  | 0.97 (0.88-1.08) | | 0.596 | | 8.7 | | 0.295 | |
| M00299: Spermidine/putrescine transport system | 1.03 (0.84-1.25) | 0.798 | 53.1 | 0.144 | |  | 1 (0.88-1.13) | | 0.979 | | 7.2 | | 0.299 | |
| M00151: Cytochrome bc1 complex respiratory unit | 0.97 (0.73-1.27) | 0.801 | 27.3 | 0.241 | |  | 0.98 (0.8-1.21) | | 0.881 | | 1.3 | | 0.314 | |
| M00033: Ectoine biosynthesis, aspartate => ectoine | 1.04 (0.75-1.45) | 0.804 | 0.0 | 0.574 | |  | 0.98 (0.73-1.3) | | 0.865 | | 0.0 | | 0.760 | |
| M00028: Ornithine biosynthesis, glutamate => ornithine | 0.96 (0.72-1.29) | 0.809 | 0.0 | 0.744 | |  | 0.95 (0.73-1.23) | | 0.675 | | 0.0 | | 0.765 | |
| M00490: MalK-MalR (malate transport) two-component regulatory system | 1.02 (0.89-1.16) | 0.809 | 10.2 | 0.291 | |  | 0.99 (0.88-1.11) | | 0.880 | | 0.0 | | 0.406 | |
| M00196: Raffinose/stachyose/melibiose transport system | 1.05 (0.69-1.62) | 0.810 | 54.4 | 0.139 | |  | 1.03 (0.75-1.41) | | 0.867 | | 33.7 | | 0.219 | |
| M00346: Formaldehyde assimilation, serine pathway | 0.96 (0.7-1.32) | 0.814 | 0.0 | 0.545 | |  | 0.93 (0.7-1.22) | | 0.595 | | 0.0 | | 0.516 | |
| M00019: Valine/isoleucine biosynthesis, pyruvate => valine / 2-oxobutanoate => isoleucine | 1.04 (0.74-1.45) | 0.818 | 0.0 | 0.486 | |  | 1 (0.75-1.34) | | 0.995 | | 0.0 | | 0.436 | |
| M00048: Inosine monophosphate biosynthesis, PRPP + glutamine => IMP | 1.03 (0.78-1.38) | 0.820 | 0.0 | 0.423 | |  | 1 (0.77-1.29) | | 0.982 | | 0.0 | | 0.438 | |
| M00512: CckA-CtrA/CpdR (cell cycle control) two-component regulatory system | 1.04 (0.73-1.5) | 0.821 | 76.9 | 0.038 | |  | 0.97 (0.78-1.2) | | 0.760 | | 51.5 | | 0.151 | |
| M00133: Polyamine biosynthesis, arginine => agmatine => putrescine => spermidine | 1.07 (0.59-1.95) | 0.824 | 81.4 | 0.020 | |  | 1.14 (0.61-2.13) | | 0.691 | | 86.3 | | 0.007 | |
| M00018: Threonine biosynthesis, aspartate => homoserine => threonine | 0.96 (0.69-1.35) | 0.824 | 2.8 | 0.310 | |  | 0.91 (0.68-1.23) | | 0.549 | | 0.0 | | 0.341 | |
| M00197: Putative fructooligosaccharide transport system | 1.05 (0.66-1.68) | 0.828 | 56.3 | 0.130 | |  | 1.01 (0.7-1.46) | | 0.947 | | 44.8 | | 0.178 | |
| M00088: Ketone body biosynthesis, acetyl-CoA => acetoacetate/3-hydroxybutyrate/acetone | 0.97 (0.75-1.26) | 0.828 | 0.0 | 0.828 | |  | 1.02 (0.81-1.28) | | 0.875 | | 0.0 | | 0.666 | |
| M00178: Ribosome, bacteria | 1.03 (0.77-1.39) | 0.832 | 0.0 | 0.376 | |  | 0.97 (0.74-1.27) | | 0.837 | | 0.0 | | 0.323 | |
| M00121: Heme biosynthesis, glutamate => protoheme/siroheme | 1.03 (0.77-1.38) | 0.832 | 0.0 | 0.570 | |  | 1.02 (0.79-1.32) | | 0.889 | | 0.0 | | 0.548 | |
| M00319: Manganese/zinc/iron transport system | 0.99 (0.87-1.12) | 0.837 | 46.4 | 0.172 | |  | 1.01 (0.93-1.1) | | 0.826 | | 3.9 | | 0.308 | |
| M00480: VraS-VraR (cell-wall peptidoglycan synthesis) two-component regulatory system | 0.97 (0.69-1.34) | 0.837 | 87.2 | 0.005 | |  | 0.91 (0.63-1.31) | | 0.615 | | 89.3 | | 0.002 | |
| M00475: BarA-UvrY (central carbon metabolism) two-component regulatory system | 1.01 (0.91-1.12) | 0.839 | 0.0 | 0.502 | |  | 1.03 (0.94-1.13) | | 0.521 | | 0.0 | | 0.985 | |
| M00283: PTS system, ascorbate-specific II component | 0.99 (0.87-1.12) | 0.844 | 0.0 | 0.667 | |  | 1 (0.9-1.12) | | 0.965 | | 0.0 | | 0.915 | |
| M00444: PhoQ-PhoP (magnesium transport) two-component regulatory system | 1.01 (0.92-1.11) | 0.844 | 0.0 | 0.777 | |  | 1.01 (0.92-1.1) | | 0.897 | | 0.0 | | 0.819 | |
| M00152: Cytochrome bc1 complex | 0.97 (0.74-1.29) | 0.845 | 68.7 | 0.074 | |  | 0.96 (0.81-1.14) | | 0.653 | | 37.4 | | 0.206 | |
| M00232: General L-amino acid transport system | 1.01 (0.9-1.13) | 0.853 | 0.0 | 0.577 | |  | 1.02 (0.92-1.13) | | 0.699 | | 0.0 | | 0.818 | |
| M00240: Iron complex transport system | 0.97 (0.67-1.39) | 0.855 | 61.8 | 0.106 | |  | 0.96 (0.73-1.26) | | 0.753 | | 49.4 | | 0.160 | |
| M00554: Nucleotide sugar biosynthesis, galactose => UDP-galactose | 1.03 (0.77-1.37) | 0.856 | 57.7 | 0.124 | |  | 0.97 (0.8-1.17) | | 0.721 | | 19.7 | | 0.264 | |
| M00051: Uridine monophosphate biosynthesis, glutamine (+ PRPP) => UMP | 1.03 (0.75-1.41) | 0.857 | 0.0 | 0.344 | |  | 0.98 (0.75-1.3) | | 0.913 | | 0.0 | | 0.368 | |
| M00010: Citrate cycle, first carbon oxidation, oxaloacetate => 2-oxoglutarate | 1.03 (0.72-1.49) | 0.858 | 0.0 | 0.690 | |  | 0.95 (0.68-1.32) | | 0.741 | | 0.0 | | 0.693 | |
| M00002: Glycolysis, core module involving three-carbon compounds | 1.03 (0.76-1.38) | 0.858 | 0.0 | 0.587 | |  | 0.99 (0.76-1.29) | | 0.941 | | 0.0 | | 0.563 | |
| M00328: Hemophore/metalloprotease transport system | 0.98 (0.78-1.23) | 0.859 | 75.9 | 0.041 | |  | 1 (0.81-1.24) | | 0.997 | | 76.7 | | 0.038 | |
| M00243: Manganese/iron transport system | 0.99 (0.89-1.1) | 0.862 | 0.0 | 0.629 | |  | 1.02 (0.92-1.12) | | 0.757 | | 5.8 | | 0.303 | |
| M00374: Dicarboxylate-hydroxybutyrate cycle | 0.97 (0.71-1.34) | 0.865 | 0.0 | 0.670 | |  | 0.97 (0.73-1.29) | | 0.833 | | 0.0 | | 0.469 | |
| M00035: Methionine degradation | 1.03 (0.76-1.39) | 0.868 | 0.0 | 0.401 | |  | 0.98 (0.75-1.27) | | 0.865 | | 0.0 | | 0.363 | |
| M00290: Holo-TFIIH complex | 1.01 (0.86-1.19) | 0.868 | 34.4 | 0.217 | |  | 1.06 (0.88-1.27) | | 0.560 | | 57.4 | | 0.125 | |
| M00394: RNA degradosome | 0.97 (0.72-1.33) | 0.869 | 0.0 | 0.393 | |  | 0.93 (0.71-1.21) | | 0.584 | | 0.0 | | 0.409 | |
| M00453: QseC-QseB (quorum sensing) two-component regulatory system | 0.98 (0.82-1.18) | 0.870 | 43.9 | 0.182 | |  | 0.99 (0.89-1.11) | | 0.921 | | 0.0 | | 0.558 | |
| M00486: CitA-CitB (citrate fermentation) two-component regulatory system | 0.99 (0.83-1.18) | 0.881 | 63.6 | 0.097 | |  | 0.91 (0.76-1.1) | | 0.325 | | 67.0 | | 0.082 | |
| M00025: Tyrosine biosynthesis, chorismate => tyrosine | 0.98 (0.71-1.34) | 0.881 | 0.0 | 0.569 | |  | 0.95 (0.71-1.26) | | 0.726 | | 0.0 | | 0.424 | |
| M00246: Nickel transport system | 1.01 (0.88-1.16) | 0.883 | 0.0 | 0.990 | |  | 0.99 (0.88-1.11) | | 0.836 | | 0.0 | | 0.341 | |
| M00245: Cobalt/nickel transport system | 0.99 (0.86-1.13) | 0.883 | 0.0 | 0.808 | |  | 0.98 (0.85-1.13) | | 0.793 | | 23.1 | | 0.254 | |
| M00308: Semi-phosphorylative Entner-Doudoroff pathway, gluconate => glycerate-3P | 1.01 (0.83-1.24) | 0.884 | 0.0 | 0.430 | |  | 0.99 (0.81-1.21) | | 0.896 | | 19.0 | | 0.267 | |
| M00249: Capsular polysaccharide transport system | 1.01 (0.88-1.16) | 0.887 | 46.9 | 0.170 | |  | 0.98 (0.85-1.13) | | 0.808 | | 59.1 | | 0.118 | |
| M00009: Citrate cycle (TCA cycle, Krebs cycle) | 1.02 (0.74-1.42) | 0.889 | 0.0 | 0.847 | |  | 0.95 (0.71-1.28) | | 0.754 | | 0.0 | | 0.750 | |
| M00078: Heparan sulfate degradation | 1.01 (0.9-1.13) | 0.890 | 0.0 | 0.391 | |  | 0.99 (0.9-1.1) | | 0.894 | | 0.0 | | 0.332 | |
| M00219: AI-2 transport system | 0.98 (0.78-1.24) | 0.892 | 77.2 | 0.036 | |  | 0.95 (0.77-1.16) | | 0.593 | | 73.4 | | 0.053 | |
| M00377: Reductive acetyl-CoA pathway (Wood-Ljungdahl pathway) | 0.98 (0.68-1.41) | 0.895 | 40.8 | 0.194 | |  | 0.96 (0.7-1.33) | | 0.815 | | 41.2 | | 0.192 | |
| M00280: PTS system, glucitol/sorbitol-specific II component | 0.99 (0.89-1.11) | 0.895 | 0.0 | 0.419 | |  | 1.05 (0.85-1.29) | | 0.672 | | 76.4 | | 0.039 | |
| M00488: DcuS-DcuR (C4-dicarboxylate metabolism) two-component regulatory system | 1.02 (0.79-1.31) | 0.896 | 77.1 | 0.037 | |  | 0.95 (0.74-1.23) | | 0.717 | | 79.2 | | 0.029 | |
| M00260: DNA polymerase III complex, bacteria | 0.98 (0.7-1.36) | 0.900 | 0.0 | 0.460 | |  | 0.98 (0.72-1.32) | | 0.889 | | 6.2 | | 0.302 | |
| M00335: Sec (secretion) system | 0.98 (0.73-1.32) | 0.901 | 0.0 | 0.346 | |  | 0.95 (0.73-1.24) | | 0.715 | | 0.0 | | 0.329 | |
| M00189: Molybdate transport system | 0.98 (0.69-1.39) | 0.902 | 33.3 | 0.221 | |  | 1 (0.73-1.37) | | 0.997 | | 34.3 | | 0.217 | |
| M00454: KdpD-KdpE (potassium transport) two-component regulatory system | 1.01 (0.87-1.17) | 0.903 | 0.0 | 0.700 | |  | 1.03 (0.9-1.16) | | 0.694 | | 0.0 | | 0.807 | |
| M00095: C5 isoprenoid biosynthesis, mevalonate pathway | 1.01 (0.8-1.29) | 0.905 | 0.0 | 0.856 | |  | 1.05 (0.85-1.3) | | 0.629 | | 0.0 | | 0.646 | |
| M00179: Ribosome, archaea | 1.02 (0.75-1.38) | 0.906 | 0.0 | 0.442 | |  | 0.96 (0.73-1.26) | | 0.756 | | 0.0 | | 0.403 | |
| M00023: Tryptophan biosynthesis, chorismate => tryptophan | 0.98 (0.68-1.4) | 0.907 | 33.9 | 0.219 | |  | 0.97 (0.7-1.36) | | 0.871 | | 41.6 | | 0.191 | |
| M00125: Riboflavin biosynthesis, GTP => riboflavin/FMN/FAD | 1.02 (0.76-1.36) | 0.909 | 0.0 | 0.609 | |  | 0.99 (0.76-1.29) | | 0.947 | | 0.0 | | 0.577 | |
| M00326: RTX toxin transport system | 0.98 (0.66-1.44) | 0.912 | 77.9 | 0.033 | |  | 1.02 (0.73-1.41) | | 0.929 | | 73.8 | | 0.051 | |
| M00188: NitT/TauT family transport system | 1.04 (0.5-2.17) | 0.913 | 83.9 | 0.013 | |  | 1.18 (0.6-2.31) | | 0.626 | | 83.8 | | 0.013 | |
| M00205: N-Acetylglucosamine transport system | 1.01 (0.88-1.16) | 0.913 | 0.0 | 0.626 | |  | 0.98 (0.86-1.11) | | 0.742 | | 0.0 | | 0.500 | |
| M00537: Xylene degradation, xylene => methylbenzoate | 1.01 (0.86-1.19) | 0.914 | 0.0 | 0.581 | |  | 0.97 (0.84-1.13) | | 0.734 | | 0.0 | | 0.841 | |
| M00315: Uncharacterized ABC transport system | 0.99 (0.89-1.11) | 0.914 | 0.0 | 0.680 | |  | 0.96 (0.86-1.07) | | 0.432 | | 0.0 | | 0.669 | |
| M00212: Ribose transport system | 0.99 (0.82-1.19) | 0.915 | 0.0 | 0.903 | |  | 1.04 (0.87-1.23) | | 0.692 | | 0.0 | | 0.936 | |
| M00017: Methionine biosynthesis, apartate => homoserine => methionine | 1.02 (0.67-1.57) | 0.918 | 40.8 | 0.194 | |  | 0.96 (0.69-1.35) | | 0.826 | | 26.7 | | 0.243 | |
| M00373: Ethylmalonyl pathway | 0.99 (0.76-1.28) | 0.918 | 0.0 | 0.772 | |  | 1.05 (0.83-1.33) | | 0.677 | | 0.0 | | 0.528 | |
| M00119: Pantothenate biosynthesis, valine/L-aspartate => pantothenate | 1.02 (0.74-1.4) | 0.924 | 0.0 | 0.318 | |  | 0.96 (0.73-1.28) | | 0.791 | | 0.0 | | 0.353 | |
| M00275: PTS system, cellobiose-specific II component | 1.01 (0.9-1.13) | 0.926 | 0.0 | 0.373 | |  | 0.99 (0.82-1.19) | | 0.922 | | 65.1 | | 0.091 | |
| M00449: CreC-CreB (phosphate regulation) two-component regulatory system | 1 (0.9-1.1) | 0.926 | 0.0 | 0.961 | |  | 0.98 (0.89-1.07) | | 0.612 | | 0.0 | | 0.887 | |
| M00456: ArcB-ArcA (anoxic redox control) two-component regulatory system | 0.99 (0.76-1.28) | 0.927 | 82.9 | 0.015 | |  | 0.97 (0.77-1.23) | | 0.832 | | 83.9 | | 0.013 | |
| M00451: BasS-BasR (antimicrobial peptide resistance) two-component regulatory system | 1.01 (0.84-1.22) | 0.930 | 57.8 | 0.124 | |  | 0.97 (0.81-1.17) | | 0.762 | | 62.6 | | 0.102 | |
| M00120: Coenzyme A biosynthesis, pantothenate => CoA | 0.98 (0.69-1.41) | 0.932 | 22.2 | 0.257 | |  | 0.94 (0.68-1.3) | | 0.694 | | 23.8 | | 0.252 | |
| M00136: GABA biosynthesis, prokaryotes, putrescine => GABA | 1 (0.9-1.1) | 0.933 | 0.0 | 0.396 | |  | 1.01 (0.92-1.11) | | 0.775 | | 0.0 | | 0.400 | |
| M00571: AlgE-type Mannuronan C-5-Epimerase transport system | 0.98 (0.65-1.48) | 0.933 | 79.5 | 0.027 | |  | 1.02 (0.73-1.42) | | 0.923 | | 73.7 | | 0.051 | |
| M00200: Putative sorbitol/mannitol transport system | 1.01 (0.87-1.17) | 0.934 | 0.0 | 0.458 | |  | 1.03 (0.86-1.24) | | 0.714 | | 22.9 | | 0.255 | |
| M00339: RaxAB-RaxC type I secretion system | 0.99 (0.73-1.33) | 0.935 | 70.9 | 0.064 | |  | 1.03 (0.81-1.31) | | 0.801 | | 61.9 | | 0.105 | |
| M00252: Lipooligosaccharide transport system | 1 (0.9-1.1) | 0.936 | 52.6 | 0.146 | |  | 1.03 (0.9-1.17) | | 0.683 | | 75.1 | | 0.045 | |
| M00551: Benzoate degradation, benzoate => catechol / methylbenzoate => methylcatechol | 1 (0.93-1.07) | 0.939 | 0.0 | 0.868 | |  | 1 (0.94-1.07) | | 0.902 | | 0.0 | | 0.547 | |
| M00061: D-Glucuronate degradation, D-glucuronate => pyruvate + D-glyceraldehyde 3P | 0.99 (0.85-1.17) | 0.940 | 38.8 | 0.201 | |  | 1.01 (0.84-1.21) | | 0.948 | | 57.5 | | 0.125 | |
| M00417: Cytochrome o ubiquinol oxidase | 0.99 (0.8-1.23) | 0.941 | 67.7 | 0.078 | |  | 1.01 (0.81-1.27) | | 0.906 | | 73.8 | | 0.051 | |
| M00117: Ubiquinone biosynthesis, prokaryotes, chorismate => ubiquinone | 0.99 (0.7-1.4) | 0.942 | 0.0 | 0.678 | |  | 0.95 (0.69-1.31) | | 0.754 | | 0.0 | | 0.980 | |
| M00157: F-type ATPase, prokaryotes and chloroplasts | 1.01 (0.71-1.45) | 0.942 | 21.3 | 0.260 | |  | 0.96 (0.69-1.34) | | 0.803 | | 28.5 | | 0.237 | |
| M00086: beta-Oxidation, acyl-CoA synthesis | 1.01 (0.73-1.41) | 0.943 | 37.9 | 0.205 | |  | 1 (0.78-1.27) | | 0.987 | | 14.2 | | 0.280 | |
| M00336: Twin-arginine translocation (Tat) system | 0.99 (0.78-1.26) | 0.944 | 0.0 | 0.821 | |  | 0.98 (0.79-1.21) | | 0.851 | | 0.0 | | 0.725 | |
| M00256: Cell division transport system | 0.99 (0.75-1.31) | 0.945 | 47.3 | 0.168 | |  | 0.96 (0.72-1.29) | | 0.796 | | 59.2 | | 0.117 | |
| M00029: Urea cycle | 1.01 (0.76-1.35) | 0.947 | 0.0 | 0.338 | |  | 0.93 (0.72-1.22) | | 0.615 | | 0.0 | | 0.439 | |
| M00460: MprB-MprA (maintenance of persistent infection) two-component regulatory system | 1 (0.89-1.12) | 0.947 | 0.0 | 0.605 | |  | 1.02 (0.92-1.13) | | 0.740 | | 0.0 | | 0.543 | |
| M00015: Proline biosynthesis, glutamate => proline | 0.99 (0.74-1.33) | 0.950 | 0.0 | 0.607 | |  | 0.95 (0.73-1.24) | | 0.703 | | 0.0 | | 0.560 | |
| M00333: Type IV secretion system | 1.01 (0.77-1.32) | 0.951 | 63.1 | 0.100 | |  | 0.98 (0.78-1.23) | | 0.865 | | 60.3 | | 0.113 | |
| M00229: Arginine transport system | 0.99 (0.84-1.17) | 0.952 | 73.2 | 0.054 | |  | 0.97 (0.81-1.16) | | 0.741 | | 80.4 | | 0.024 | |
| M00199: L-Arabinose/lactose transport system | 1.01 (0.8-1.26) | 0.953 | 69.4 | 0.070 | |  | 1.01 (0.86-1.18) | | 0.930 | | 52.1 | | 0.148 | |
| M00226: Histidine transport system | 1 (0.92-1.08) | 0.958 | 0.0 | 0.967 | |  | 1 (0.93-1.08) | | 0.900 | | 0.0 | | 0.663 | |
| M00112: Tocopherol/tocotorienol biosynthesis | 1.01 (0.64-1.6) | 0.960 | 88.8 | 0.003 | |  | 0.97 (0.58-1.63) | | 0.915 | | 91.6 | | 0.001 | |
| M00519: YesM-YesN two-component regulatory system | 1 (0.9-1.12) | 0.965 | 0.0 | 0.484 | |  | 1 (0.83-1.19) | | 0.963 | | 67.9 | | 0.078 | |
| M00153: Cytochrome d ubiquinol oxidase | 1 (0.85-1.18) | 0.966 | 0.0 | 0.434 | |  | 1.01 (0.87-1.17) | | 0.903 | | 0.0 | | 0.631 | |
| M00093: Phosphatidylethanolamine (PE) biosynthesis, PA => PS => PE | 1.01 (0.74-1.37) | 0.967 | 0.0 | 0.481 | |  | 0.95 (0.72-1.25) | | 0.694 | | 0.0 | | 0.427 | |
| M00214: Methyl-galactoside transport system | 1 (0.82-1.23) | 0.969 | 79.1 | 0.029 | |  | 0.96 (0.78-1.18) | | 0.696 | | 82.3 | | 0.018 | |
| M00140: C1-unit interconversion, prokaryotes | 0.99 (0.63-1.57) | 0.981 | 58.1 | 0.122 | |  | 0.95 (0.64-1.41) | | 0.804 | | 54.2 | | 0.140 | |
| M00533: Homoprotocatechuate degradation, homoprotocatechuate => 2-oxohept-3-enedioate | 1 (0.75-1.34) | 0.982 | 65.1 | 0.090 | |  | 1.05 (0.78-1.43) | | 0.743 | | 73.2 | | 0.054 | |
| M00234: Cystine transport system | 1 (0.87-1.15) | 0.984 | 55.5 | 0.134 | |  | 0.97 (0.82-1.15) | | 0.738 | | 75.9 | | 0.042 | |
| M00524: FixL-FixJ (nitrogen fixation) two-component regulatory system | 1 (0.91-1.1) | 0.985 | 0.0 | 0.690 | |  | 1 (0.92-1.09) | | 0.947 | | 0.0 | | 0.611 | |
| M00172: C4-dicarboxylic acid cycle, NADP - malic enzyme type | 1 (0.77-1.3) | 0.988 | 0.0 | 0.319 | |  | 1 (0.79-1.27) | | 0.993 | | 2.5 | | 0.311 | |
| M00515: FlrB-FlrC (polar flagellar synthesis) two-component regulatory system | 1 (0.83-1.2) | 0.993 | 55.9 | 0.132 | |  | 1.01 (0.89-1.16) | | 0.867 | | 34.1 | | 0.218 | |
| M00459: VicK-VicR (cell wall metabolism) two-component regulatory system | 1 (0.91-1.1) | 0.996 | 0.0 | 0.447 | |  | 1.01 (0.9-1.13) | | 0.905 | | 33.8 | | 0.219 | |
| M00046: Pyrimidine degradation, uracil => beta-alanine, thymine => 3-aminoisobutanoate | 1 (0.87-1.15) | 0.997 | 0.0 | 0.864 | |  | 0.97 (0.85-1.1) | | 0.618 | | 0.0 | | 0.820 | |
| M00026: Histidine biosynthesis, PRPP => histidine | 1 (0.74-1.35) | 0.997 | 0.0 | 0.375 | |  | 0.98 (0.76-1.28) | | 0.904 | | 0.0 | | 0.326 | |
| M00322: Neutral amino acid transport system | 1 (0.72-1.39) | 0.999 | 80.8 | 0.022 | |  | 0.91 (0.58-1.41) | | 0.662 | | 88.8 | | 0.003 | |
| M00498: NtrY-NtrX (nitrogen regulation) two-component regulatory system | 1 (0.81-1.24) | 0.999 | 63.8 | 0.096 | |  | 0.98 (0.79-1.21) | | 0.834 | | 68.1 | | 0.076 | |
| M00150: Fumarate reductase, prokaryotes | 1 (0.92-1.09) | 1.000 | 3.8 | 0.308 | |  | 0.96 (0.89-1.04) | | 0.327 | | 0.0 | | 0.803 | |
| **KEGG Pathway Name** | Meta-analysis for OS^a^ | | | | |  | | Meta-analysis for RFS^a^ | | | | | | |
|  | HR (95%CI) | *P*_meta_ | *I*^2^ | | *P*_het_ |  | | HR (95%CI) | | *P*_meta_ | | *I*^2^ | | *P*_het_ |
| 2.1.4Amidinotransferases | **1.22 (1.07-1.39)** | **3.50E-03** | 0.0 | | 0.985 |  | | **1.19 (1.05-1.34)** | | **0.008** | | 0.0 | | 0.394 |
| 1.14.16With reduced pteridine as one donor, and incorporation of one atom of oxygen into the other donor | **0.88 (0.78-0.98)** | **0.024** | 0.0 | | 0.739 |  | | **0.89 (0.8-0.99)** | | **0.027** | | 0.0 | | 0.804 |
| 5.3.2Interconverting keto- and enol-groups | **0.87 (0.77-0.99)** | **0.034** | 0.0 | | 0.564 |  | | 0.9 (0.8-1.01) | | 0.077 | | 0.0 | | 0.859 |
| 1.9.3With oxygen as acceptor | **0.86 (0.75-0.99)** | **0.040** | 0.0 | | 0.416 |  | | 0.91 (0.77-1.08) | | 0.268 | | 27.4 | | 0.241 |
| 5.4.4Transferring hydroxy groups | **1.26 (1.01-1.58)** | **0.042** | 0.0 | | 0.920 |  | | 1.18 (0.97-1.44) | | 0.098 | | 0.0 | | 0.766 |
| 1.12.1With NAD+ or NADP+ as acceptor | **0.87 (0.77-1)** | **0.047** | 0.0 | | 0.953 |  | | **0.88 (0.78-0.99)** | | **0.038** | | 0.0 | | 1.000 |
| 1.18.1With NAD+ or NADP+ as acceptor | 1.31 (0.99-1.73) | 0.055 | 0.0 | | 0.444 |  | | 1.24 (0.96-1.6) | | 0.096 | | 0.0 | | 0.319 |
| 5.3.4Transposing S-S bonds | 0.88 (0.78-1) | 0.055 | 0.0 | | 0.585 |  | | 0.9 (0.8-1) | | 0.060 | | 0.0 | | 0.969 |
| 1.7.2With a cytochrome as acceptor | 0.91 (0.82-1.01) | 0.067 | 0.0 | | 0.886 |  | | 0.95 (0.87-1.05) | | 0.304 | | 0.0 | | 0.900 |
| 3.5.3In linear amidines | 1.41 (0.96-2.07) | 0.076 | 0.0 | | 0.421 |  | | 1.21 (0.76-1.92) | | 0.432 | | 43.3 | | 0.184 |
| 4.3.99Other carbon-nitrogen lyases | 0.9 (0.81-1.01) | 0.081 | 14.9 | | 0.278 |  | | 0.92 (0.84-1) | | 0.063 | | 0.0 | | 0.738 |
| 5.1.2Acting on hydroxy acids and derivatives | 0.9 (0.79-1.02) | 0.093 | 14.4 | | 0.280 |  | | 0.96 (0.82-1.13) | | 0.653 | | 55.5 | | 0.134 |
| 1.2.5With a quinone or similar compound as acceptor | 1.2 (0.97-1.48) | 0.096 | 0.0 | | 0.630 |  | | 1.17 (0.97-1.42) | | 0.094 | | 0.0 | | 0.598 |
| 1.4.4With a disulfide as acceptor | 1.25 (0.96-1.63) | 0.096 | 7.6 | | 0.298 |  | | 1.26 (0.92-1.72) | | 0.153 | | 44.8 | | 0.178 |
| 1.6.1With NAD+ or NADP+ as acceptor | 0.7 (0.45-1.08) | 0.107 | 64.7 | | 0.092 |  | | 0.75 (0.46-1.22) | | 0.248 | | 77.8 | | 0.034 |
| 1.5.5With a quinone or similar compound as acceptor | 0.9 (0.78-1.03) | 0.133 | 15.8 | | 0.276 |  | | 0.94 (0.84-1.05) | | 0.241 | | 0.0 | | 0.491 |
| 1.1.5With a quinone or similar compound as acceptor | 1.27 (0.93-1.75) | 0.137 | 0.0 | | 0.399 |  | | 1.19 (0.89-1.59) | | 0.240 | | 1.3 | | 0.314 |
| 3.5.2In cyclic amides | 1.34 (0.91-1.97) | 0.139 | 0.0 | | 0.886 |  | | 1.15 (0.82-1.61) | | 0.427 | | 0.0 | | 0.633 |
| 3.4.16Serine-type carboxypeptidases | 1.32 (0.91-1.9) | 0.143 | 0.0 | | 0.832 |  | | 1.17 (0.84-1.63) | | 0.358 | | 0.0 | | 0.397 |
| 2.6.99Transferring other nitrogenous groups | 0.91 (0.81-1.04) | 0.161 | 0.0 | | 0.368 |  | | 0.93 (0.83-1.04) | | 0.218 | | 0.0 | | 0.474 |
| 3.5.5In nitriles | 0.92 (0.82-1.03) | 0.167 | 3.7 | | 0.308 |  | | 0.97 (0.79-1.2) | | 0.793 | | 69.9 | | 0.068 |
| 2.10.1Molybdenumtransferases or tungstentransferases withsulfide groups as acceptors | 0.9 (0.78-1.04) | 0.169 | 0.0 | | 0.519 |  | | 0.94 (0.82-1.09) | | 0.422 | | 9.7 | | 0.293 |
| 1.8.7With an iron-sulfur protein as acceptor | 1.13 (0.95-1.34) | 0.171 | 0.0 | | 0.897 |  | | 1.13 (0.97-1.31) | | 0.122 | | 0.0 | | 0.632 |
| 1.7.7With an iron-sulfur protein as acceptor | 1.14 (0.94-1.38) | 0.172 | 32.7 | | 0.223 |  | | 1.19 (1.05-1.34) | | 0.005 | | 0.0 | | 0.355 |
| 5.3.99Other intramolecular oxidoreductases | 0.92 (0.82-1.04) | 0.186 | 0.0 | | 0.827 |  | | 0.92 (0.83-1.02) | | 0.104 | | 0.0 | | 0.524 |
| 1.16.3With oxygen as acceptor | 1.13 (0.93-1.37) | 0.217 | 0.0 | | 0.998 |  | | 1.1 (0.93-1.31) | | 0.264 | | 0.0 | | 0.641 |
| 3.4.25Threonine endopeptidases | 0.88 (0.72-1.08) | 0.218 | 0.0 | | 0.324 |  | | 0.9 (0.73-1.12) | | 0.362 | | 26.7 | | 0.243 |
| 2.7.-Transferring phosphorus-containing groups | 0.92 (0.81-1.05) | 0.218 | 0.0 | | 0.487 |  | | 0.94 (0.84-1.05) | | 0.288 | | 0.0 | | 0.405 |
| 5.4.1Transferring acyl groups | 0.91 (0.79-1.06) | 0.227 | 0.0 | | 0.685 |  | | 0.96 (0.84-1.1) | | 0.592 | | 0.0 | | 0.959 |
| 4.-.- | 1.22 (0.88-1.68) | 0.231 | 34.7 | | 0.216 |  | | 1.17 (0.93-1.47) | | 0.171 | | 0.0 | | 0.348 |
| 3.4.-Acting on peptide bonds (peptidases) | 0.84 (0.64-1.12) | 0.237 | 47.5 | | 0.167 |  | | 0.86 (0.68-1.09) | | 0.204 | | 39.6 | | 0.198 |
| 1.21.4With a disulfide as acceptor | 1.07 (0.96-1.18) | 0.241 | 0.0 | | 0.845 |  | | 1.01 (0.87-1.17) | | 0.918 | | 56.6 | | 0.129 |
| 6.3.1Acid-D-ammonia (or amine) ligases (amide synthases) | 1.24 (0.87-1.77) | 0.242 | 0.0 | | 0.992 |  | | 1.15 (0.84-1.58) | | 0.372 | | 0.0 | | 0.952 |
| 3.5.-Acting on carbon-nitrogen bonds, other than peptidebonds | 1.06 (0.96-1.17) | 0.244 | 0.0 | | 0.993 |  | | 1 (0.92-1.1) | | 0.926 | | 0.0 | | 0.452 |
| 4.1.2Aldehyde-lyases | 1.28 (0.85-1.92) | 0.245 | 0.0 | | 0.920 |  | | 1.1 (0.76-1.6) | | 0.601 | | 0.0 | | 0.954 |
| 1.13.11With incorporation of two atoms of oxygen | 0.91 (0.77-1.07) | 0.245 | 31.0 | | 0.229 |  | | 0.95 (0.85-1.06) | | 0.380 | | 0.0 | | 0.386 |
| 1.1.-Acting on the CH-OH group of donors | 0.95 (0.86-1.04) | 0.259 | 0.0 | | 0.766 |  | | 0.98 (0.9-1.07) | | 0.661 | | 0.0 | | 0.738 |
| 2.6.-Transferring nitrogenous groups | 1.15 (0.9-1.48) | 0.260 | 0.0 | | 0.821 |  | | 1.06 (0.85-1.32) | | 0.629 | | 0.0 | | 0.336 |
| 1.3.5With a quinone or related compound as acceptor | 1.13 (0.91-1.4) | 0.262 | 0.0 | | 0.527 |  | | 1.17 (0.96-1.41) | | 0.116 | | 0.0 | | 0.344 |
| 2.8.1Sulfurtransferases | 1.24 (0.85-1.82) | 0.264 | 0.0 | | 0.336 |  | | 1.12 (0.8-1.57) | | 0.511 | | 0.0 | | 0.404 |
| 3.1.13Exoribonucleases producing 5-phosphomonoesters | 1.23 (0.85-1.77) | 0.265 | 46.4 | | 0.172 |  | | 1.17 (0.87-1.58) | | 0.310 | | 41.3 | | 0.192 |
| 1.7.99With other acceptors | 0.89 (0.72-1.1) | 0.271 | 0.0 | | 0.560 |  | | 0.93 (0.77-1.12) | | 0.446 | | 0.0 | | 0.706 |
| 1.17.99With other acceptors | 0.94 (0.85-1.05) | 0.272 | 0.0 | | 0.430 |  | | 0.97 (0.85-1.11) | | 0.662 | | 50.8 | | 0.154 |
| 1.5.-Acting on the CH-NH group of donors | 0.91 (0.76-1.08) | 0.272 | 0.0 | | 0.600 |  | | 0.87 (0.71-1.07) | | 0.191 | | 33.4 | | 0.220 |
| 2.2.1Transketolases and transaldolases | 1.3 (0.82-2.06) | 0.273 | 0.0 | | 0.636 |  | | 1.08 (0.71-1.65) | | 0.718 | | 0.0 | | 0.747 |
| 6.5.1Ligases that form phosphoric-ester bonds (only sub-subclass identified to date) | 1.23 (0.84-1.81) | 0.283 | 0.0 | | 0.555 |  | | 1.08 (0.77-1.51) | | 0.666 | | 0.0 | | 0.911 |
| 1.-.- | 0.92 (0.78-1.08) | 0.298 | 0.0 | | 0.726 |  | | 0.97 (0.83-1.14) | | 0.701 | | 0.0 | | 0.905 |
| 3.1.4Phosphoric-diester hydrolases | 1.21 (0.84-1.74) | 0.298 | 0.0 | | 0.617 |  | | 1.08 (0.78-1.5) | | 0.648 | | 0.0 | | 0.757 |
| 1.4.3With oxygen as acceptor | 1.2 (0.85-1.69) | 0.304 | 0.0 | | 0.378 |  | | 1.1 (0.81-1.5) | | 0.555 | | 0.0 | | 0.617 |
| 5.99.1Sole sub-subclass for isomerases that do not belonginthe other subclasses | 1.23 (0.82-1.84) | 0.310 | 0.0 | | 0.899 |  | | 1.06 (0.74-1.52) | | 0.733 | | 0.0 | | 0.571 |
| 1.6.99With other acceptors | 1.19 (0.85-1.68) | 0.316 | 0.0 | | 0.412 |  | | 1.02 (0.74-1.39) | | 0.920 | | 0.0 | | 0.867 |
| 1.4.9With a copper protein as acceptor | 0.95 (0.87-1.05) | 0.318 | 0.0 | | 0.719 |  | | 0.96 (0.88-1.04) | | 0.304 | | 0.0 | | 0.843 |
| 4.2.2Acting on polysaccharides | 1.07 (0.94-1.21) | 0.319 | 0.0 | | 0.563 |  | | 1.01 (0.91-1.13) | | 0.798 | | 0.0 | | 0.511 |
| 3.4.11Aminopeptidases | 1.22 (0.81-1.85) | 0.340 | 0.0 | | 0.659 |  | | 1.02 (0.7-1.49) | | 0.913 | | 0.0 | | 0.823 |
| 1.2.3With oxygen as acceptor | 1.05 (0.95-1.17) | 0.340 | 30.8 | | 0.229 |  | | 0.98 (0.77-1.24) | | 0.847 | | 79.7 | | 0.027 |
| 2.3.2Aminoacyltransferases | 0.94 (0.83-1.07) | 0.342 | 0.0 | | 0.661 |  | | 0.96 (0.85-1.08) | | 0.522 | | 0.0 | | 0.909 |
| 3.4.21Serine endopeptidases | 1.23 (0.8-1.88) | 0.342 | 0.0 | | 0.675 |  | | 0.98 (0.66-1.45) | | 0.909 | | 0.0 | | 0.982 |
| 3.4.13Dipeptidases | 1.12 (0.89-1.4) | 0.345 | 0.0 | | 0.891 |  | | 1.05 (0.87-1.28) | | 0.597 | | 0.0 | | 0.589 |
| 1.4.2With a cytochrome as acceptor | 1.05 (0.95-1.17) | 0.345 | 0.0 | | 0.610 |  | | 1.07 (0.96-1.19) | | 0.199 | | 12.7 | | 0.284 |
| 1.5.3With oxygen as acceptor | 1.11 (0.89-1.38) | 0.353 | 49.4 | | 0.160 |  | | 1.11 (0.93-1.32) | | 0.265 | | 36.8 | | 0.209 |
| 1.14.-Acting on paired donors, with O2 as oxidant and incorporation or reduction of oxygen. The oxygen incorporated need not be derived from O2 | 1.08 (0.92-1.27) | 0.356 | 0.0 | | 0.747 |  | | 1.05 (0.91-1.21) | | 0.541 | | 0.0 | | 0.822 |
| 1.5.99With other acceptors | 0.93 (0.8-1.09) | 0.356 | 0.0 | | 0.524 |  | | 0.95 (0.83-1.1) | | 0.493 | | 0.0 | | 0.512 |
| 1.10.9With a copper protein as acceptor | 1.11 (0.89-1.39) | 0.364 | 57.5 | | 0.125 |  | | 1.07 (0.82-1.41) | | 0.612 | | 72.6 | | 0.056 |
| 2.6.1Transaminases | 1.21 (0.8-1.82) | 0.373 | 0.0 | | 0.440 |  | | 1.02 (0.7-1.49) | | 0.904 | | 0.0 | | 0.612 |
| 4.2.1Hydro-lyases | 1.24 (0.77-1.98) | 0.375 | 0.0 | | 0.748 |  | | 1.08 (0.7-1.66) | | 0.718 | | 0.0 | | 0.718 |
| 3.4.15Peptidyl-dipeptidases | 1.18 (0.81-1.72) | 0.380 | 39.6 | | 0.198 |  | | 1.09 (0.84-1.4) | | 0.535 | | 0.0 | | 0.438 |
| 1.7.3With oxygen as acceptor | 1.08 (0.91-1.29) | 0.380 | 0.0 | | 0.924 |  | | 1.05 (0.9-1.23) | | 0.530 | | 0.0 | | 0.768 |
| 3.2.-Glycosylases | 1.13 (0.86-1.48) | 0.381 | 0.0 | | 0.426 |  | | 1.05 (0.82-1.34) | | 0.682 | | 0.0 | | 0.336 |
| 2.5.1Transferring alkyl or aryl groups, other than methylgroups (only sub-subclass identified to date) | 1.21 (0.78-1.88) | 0.390 | 0.0 | | 0.711 |  | | 1.03 (0.69-1.54) | | 0.888 | | 0.0 | | 0.947 |
| 1.14.14With reduced flavin or flavoprotein as one donor,andincorporation of one atom of oxygen into the other donor | 0.94 (0.81-1.09) | 0.391 | 12.2 | | 0.286 |  | | 0.96 (0.86-1.08) | | 0.542 | | 0.3 | | 0.317 |
| 1.3.8With a flavin as acceptor | 1.11 (0.88-1.4) | 0.392 | 0.0 | | 0.581 |  | | 1.11 (0.9-1.37) | | 0.324 | | 0.0 | | 0.569 |
| 4.1.3Oxo-acid-lyases | 1.18 (0.8-1.72) | 0.408 | 0.0 | | 0.637 |  | | 1.09 (0.77-1.55) | | 0.631 | | 0.0 | | 0.650 |
| 1.13.12With incorporation of one atom of oxygen (internalmonooxygenases or internal mixed-function oxidases) | 0.94 (0.81-1.09) | 0.412 | 0.0 | | 0.742 |  | | 0.98 (0.85-1.12) | | 0.731 | | 0.0 | | 0.721 |
| 4.6.1Phosphorus-oxygen lyases (only sub-subclass identifiedto date) | 1.1 (0.87-1.39) | 0.412 | 0.0 | | 0.997 |  | | 1.14 (0.93-1.4) | | 0.203 | | 0.0 | | 0.854 |
| 1.14.11With 2-oxoglutarate as one donor, and incorporation of one atom of oxygen into each donor | 0.95 (0.85-1.07) | 0.414 | 0.2 | | 0.317 |  | | 0.99 (0.88-1.12) | | 0.881 | | 15.0 | | 0.278 |
| 1.8.4With a disulfide as acceptor | 1.17 (0.8-1.71) | 0.418 | 0.0 | | 0.725 |  | | 1.03 (0.74-1.45) | | 0.848 | | 0.0 | | 0.804 |
| 3.4.19Omega peptidases | 1.06 (0.92-1.23) | 0.429 | 0.0 | | 0.704 |  | | 1.03 (0.9-1.18) | | 0.670 | | 0.0 | | 0.538 |
| 1.18.6With dinitrogen as acceptor | 1.06 (0.92-1.21) | 0.432 | 12.7 | | 0.285 |  | | 1.09 (0.91-1.31) | | 0.353 | | 55.9 | | 0.132 |
| 3.1.2Thioester hydrolases | 1.15 (0.81-1.63) | 0.434 | 0.0 | | 0.404 |  | | 1.02 (0.74-1.41) | | 0.903 | | 0.0 | | 0.356 |
| 3.1.27Endoribonucleases producing 3-phosphomonoesters | 0.96 (0.88-1.06) | 0.437 | 0.0 | | 0.520 |  | | 0.97 (0.85-1.12) | | 0.707 | | 43.9 | | 0.182 |
| 5.-.- | 1.06 (0.91-1.23) | 0.442 | 0.0 | | 0.443 |  | | 1.02 (0.8-1.3) | | 0.863 | | 64.4 | | 0.094 |
| 2.8.2Sulfotransferases | 0.96 (0.85-1.07) | 0.445 | 0.0 | | 0.657 |  | | 0.94 (0.78-1.13) | | 0.524 | | 54.9 | | 0.137 |
| 2.7.13Protein-histidine kinases | 1.26 (0.7-2.26) | 0.445 | 21.0 | | 0.260 |  | | 1.18 (0.69-2.01) | | 0.551 | | 22.1 | | 0.257 |
| 1.4.99With other acceptors | 0.96 (0.85-1.07) | 0.447 | 0.0 | | 0.493 |  | | 0.99 (0.89-1.1) | | 0.892 | | 0.0 | | 0.553 |
| 1.3.3With oxygen as acceptor | 1.11 (0.85-1.44) | 0.453 | 0.0 | | 0.671 |  | | 1.09 (0.87-1.38) | | 0.445 | | 0.0 | | 0.776 |
| 2.3.1Transferring groups other than aminoacyl groups | 1.19 (0.75-1.9) | 0.453 | 0.0 | | 0.782 |  | | 1.07 (0.7-1.63) | | 0.765 | | 0.0 | | 0.633 |
| 3.3.2Ether hydrolases | 1.04 (0.94-1.16) | 0.453 | 0.0 | | 0.901 |  | | 1.01 (0.91-1.11) | | 0.894 | | 0.0 | | 0.730 |
| 3.2.1Glycosidases, i.e. enzymes that hydrolyse O- and S-glycosyl compounds | 1.17 (0.78-1.76) | 0.460 | 0.0 | | 0.618 |  | | 1.02 (0.7-1.48) | | 0.928 | | 0.0 | | 0.844 |
| 4.3.1Ammonia-lyases | 1.2 (0.73-1.97) | 0.469 | 24.9 | | 0.249 |  | | 1 (0.69-1.46) | | 0.996 | | 0.0 | | 0.443 |
| 5.1.1Acting on amino acids and derivatives | 1.17 (0.77-1.78) | 0.469 | 0.0 | | 0.874 |  | | 1.06 (0.73-1.56) | | 0.757 | | 0.0 | | 0.951 |
| 1.1.1With NAD+ or NADP+ as acceptor | 1.18 (0.74-1.88) | 0.477 | 0.0 | | 0.629 |  | | 1.02 (0.67-1.56) | | 0.929 | | 0.0 | | 0.645 |
| 5.4.99Transferring other groups | 1.18 (0.74-1.88) | 0.486 | 0.0 | | 0.512 |  | | 1.04 (0.68-1.59) | | 0.856 | | 0.0 | | 0.378 |
| 3.4.24Metalloendopeptidases | 1.17 (0.75-1.81) | 0.488 | 0.0 | | 0.899 |  | | 1.1 (0.74-1.63) | | 0.639 | | 0.0 | | 0.626 |
| 4.3.2Amidine-lyases | 1.15 (0.78-1.7) | 0.490 | 0.0 | | 0.326 |  | | 0.94 (0.65-1.35) | | 0.722 | | 0.0 | | 0.559 |
| 1.2.99With other acceptors | 0.97 (0.89-1.06) | 0.493 | 9.7 | | 0.293 |  | | 1.01 (0.88-1.17) | | 0.878 | | 58.6 | | 0.120 |
| 1.3.98With other, known, acceptors | 1.07 (0.88-1.31) | 0.494 | 40.9 | | 0.193 |  | | 1.09 (0.97-1.22) | | 0.169 | | 0.0 | | 0.885 |
| 3.1.22Endodeoxyribonucleases producing 3-phosphomonoesters | 0.93 (0.76-1.14) | 0.497 | 0.0 | | 0.386 |  | | 0.92 (0.76-1.1) | | 0.359 | | 0.0 | | 0.661 |
| 1.14.15With reduced iron-sulfur protein as one donor, andincorporation of one atom of oxygen into the other donor | 1.03 (0.95-1.11) | 0.499 | 0.0 | | 0.485 |  | | 1.07 (0.98-1.17) | | 0.141 | | 25.1 | | 0.248 |
| 1.4.1With NAD+ or NADP+ as acceptor | 1.13 (0.79-1.61) | 0.501 | 0.0 | | 0.451 |  | | 1.06 (0.77-1.48) | | 0.713 | | 0.0 | | 0.392 |
| 1.20.9With a copper protein as acceptor | 0.92 (0.72-1.18) | 0.507 | 71.0 | | 0.063 |  | | 0.96 (0.88-1.06) | | 0.428 | | 0.0 | | 0.332 |
| 2.7.1Phosphotransferases with an alcohol group as acceptor | 1.17 (0.74-1.84) | 0.507 | 3.2 | | 0.309 |  | | 1.01 (0.68-1.5) | | 0.964 | | 0.0 | | 0.396 |
| 6.3.2Acid-D-amino-acid ligases (peptide synthases) | 1.14 (0.76-1.71) | 0.513 | 0.0 | | 0.334 |  | | 1.07 (0.74-1.53) | | 0.718 | | 0.0 | | 0.349 |
| 6.1.1Ligases forming aminoacyl-tRNA and related compounds | 1.14 (0.75-1.72) | 0.541 | 0.0 | | 0.371 |  | | 1.01 (0.69-1.47) | | 0.962 | | 0.0 | | 0.496 |
| 2.7.7Nucleotidyltransferases | 1.15 (0.73-1.84) | 0.543 | 0.0 | | 0.958 |  | | 1.01 (0.66-1.53) | | 0.981 | | 0.0 | | 0.998 |
| 1.2.4With a disulfide as acceptor | 1.14 (0.75-1.74) | 0.547 | 53.3 | | 0.143 |  | | 1.1 (0.73-1.66) | | 0.646 | | 57.1 | | 0.127 |
| 3.5.1In linear amides | 1.14 (0.75-1.72) | 0.549 | 0.0 | | 0.552 |  | | 0.94 (0.64-1.38) | | 0.758 | | 0.0 | | 0.956 |
| 4.2.3Acting on phosphates | 1.12 (0.78-1.61) | 0.550 | 0.0 | | 0.482 |  | | 1.05 (0.74-1.48) | | 0.793 | | 7.7 | | 0.298 |
| 5.4.2Phosphotransferases (phosphomutases) | 1.14 (0.74-1.77) | 0.555 | 0.0 | | 0.371 |  | | 1.01 (0.69-1.49) | | 0.946 | | 0.0 | | 0.484 |
| 1.14.19With oxidation of a pair of donors resulting in the reduction of O2 to two molecules of water | 1.09 (0.82-1.46) | 0.559 | 78.4 | | 0.031 |  | | 1.12 (0.89-1.41) | | 0.350 | | 71.8 | | 0.059 |
| 5.1.99Acting on other compounds | 0.97 (0.87-1.08) | 0.562 | 0.0 | | 0.855 |  | | 0.98 (0.88-1.08) | | 0.659 | | 0.0 | | 0.612 |
| 5.3.3Transposing C=C bonds | 1.1 (0.79-1.53) | 0.573 | 0.0 | | 0.888 |  | | 1.14 (0.85-1.52) | | 0.383 | | 0.0 | | 0.975 |
| 2.1.3Carboxy- and carbamoyltransferases | 1.17 (0.67-2.05) | 0.575 | 45.7 | | 0.175 |  | | 1.03 (0.68-1.54) | | 0.904 | | 21.5 | | 0.259 |
| 1.3.7With an iron-sulfur protein as acceptor | 1.03 (0.93-1.13) | 0.578 | 0.0 | | 0.445 |  | | 1.07 (0.98-1.17) | | 0.132 | | 0.0 | | 0.367 |
| 1.3.1With NAD+ or NADP+ as acceptor | 1.15 (0.69-1.94) | 0.585 | 0.0 | | 0.774 |  | | 1.01 (0.63-1.64) | | 0.959 | | 0.0 | | 0.614 |
| 3.1.11Exodeoxyribonucleases producing 5-phosphomonoesters | 1.11 (0.75-1.65) | 0.591 | 0.0 | | 0.590 |  | | 1.02 (0.72-1.46) | | 0.902 | | 0.0 | | 0.565 |
| 3.1.1Carboxylic-ester hydrolases | 0.88 (0.54-1.43) | 0.599 | 0.0 | | 0.323 |  | | 0.83 (0.54-1.26) | | 0.377 | | 0.0 | | 0.331 |
| 5.5.1Intramolecular lyases (only sub-subclass identifiedtodate) | 1.03 (0.92-1.15) | 0.607 | 0.0 | | 0.967 |  | | 1.06 (0.95-1.17) | | 0.292 | | 0.0 | | 0.843 |
| 6.3.3Cyclo-ligases | 1.1 (0.77-1.57) | 0.608 | 0.0 | | 0.971 |  | | 1.07 (0.78-1.46) | | 0.694 | | 0.0 | | 0.952 |
| 3.-.- | 1.03 (0.91-1.17) | 0.611 | 25.4 | | 0.247 |  | | 1.01 (0.79-1.28) | | 0.958 | | 81.8 | | 0.019 |
| 1.8.1With NAD+ or NADP+ as acceptor | 1.12 (0.71-1.77) | 0.614 | 0.0 | | 0.356 |  | | 1.06 (0.71-1.59) | | 0.776 | | 0.0 | | 0.414 |
| 1.2.1With NAD+ or NADP+ as acceptor | 1.12 (0.72-1.73) | 0.615 | 0.0 | | 0.643 |  | | 0.99 (0.67-1.45) | | 0.941 | | 0.0 | | 0.693 |
| 4.99.1Sole sub-subclass for lyases that do not belong intheother subclasses | 1.11 (0.72-1.7) | 0.632 | 0.0 | | 0.966 |  | | 1.09 (0.75-1.6) | | 0.643 | | 0.0 | | 0.890 |
| 3.5.99In other compounds | 1.04 (0.87-1.25) | 0.644 | 0.0 | | 0.460 |  | | 1.03 (0.89-1.19) | | 0.655 | | 0.0 | | 0.490 |
| 2.7.4Phosphotransferases with a phosphate group as acceptor | 1.11 (0.7-1.77) | 0.647 | 0.0 | | 0.726 |  | | 0.97 (0.64-1.47) | | 0.892 | | 0.0 | | 0.712 |
| 1.8.99With other acceptors | 0.97 (0.87-1.1) | 0.669 | 0.0 | | 0.530 |  | | 1.03 (0.91-1.18) | | 0.634 | | 16.7 | | 0.273 |
| 1.10.2With a cytochrome as acceptor | 0.96 (0.78-1.17) | 0.670 | 47.8 | | 0.166 |  | | 0.94 (0.83-1.06) | | 0.310 | | 5.8 | | 0.303 |
| 1.17.4With a disulfide as acceptor | 1.08 (0.76-1.54) | 0.672 | 0.0 | | 0.975 |  | | 1.02 (0.75-1.39) | | 0.891 | | 0.0 | | 0.796 |
| 1.97.1Sole sub-subclass for oxidoreductases that do not belong in the other subclasses | 1.06 (0.82-1.36) | 0.672 | 46.4 | | 0.172 |  | | 1.03 (0.85-1.24) | | 0.767 | | 13.9 | | 0.281 |
| 5.4.3Transferring amino groups | 1.08 (0.75-1.55) | 0.672 | 0.0 | | 0.600 |  | | 0.99 (0.71-1.38) | | 0.964 | | 4.0 | | 0.307 |
| 4.3.-Carbon-nitrogen lyases | 1.02 (0.94-1.1) | 0.675 | 0.0 | | 0.772 |  | | 1.06 (0.98-1.14) | | 0.151 | | 0.0 | | 0.592 |
| 4.4.1Carbon-sulfur lyases (only sub-subclass identified to date) | 0.87 (0.45-1.69) | 0.680 | 61.2 | | 0.109 |  | | 0.77 (0.44-1.33) | | 0.343 | | 53.2 | | 0.144 |
| 1.6.5With a quinone or similar compound as acceptor | 0.92 (0.62-1.38) | 0.687 | 42.1 | | 0.189 |  | | 0.89 (0.68-1.16) | | 0.378 | | 0.0 | | 0.376 |
| 6.3.5Carbon-nitrogen ligases with glutamine as amido-N-donor | 1.09 (0.72-1.65) | 0.691 | 0.0 | | 0.679 |  | | 0.98 (0.68-1.43) | | 0.923 | | 0.0 | | 0.895 |
| 1.1.99With other acceptors | 0.97 (0.82-1.14) | 0.694 | 0.0 | | 0.827 |  | | 0.99 (0.85-1.15) | | 0.909 | | 0.0 | | 0.774 |
| 1.12.99With other acceptors | 0.98 (0.91-1.06) | 0.696 | 0.0 | | 0.596 |  | | 0.99 (0.93-1.06) | | 0.850 | | 0.0 | | 0.557 |
| 4.2.99Other carbon-oxygen lyases | 1.09 (0.71-1.66) | 0.704 | 44.1 | | 0.181 |  | | 1.02 (0.75-1.4) | | 0.896 | | 17.6 | | 0.271 |
| 2.7.6Diphosphotransferases | 1.08 (0.72-1.62) | 0.712 | 5.0 | | 0.305 |  | | 0.93 (0.65-1.34) | | 0.705 | | 0.0 | | 0.401 |
| 6.2.1Acid-thiol ligases | 0.93 (0.63-1.38) | 0.712 | 0.0 | | 0.597 |  | | 0.88 (0.62-1.27) | | 0.507 | | 0.0 | | 0.586 |
| 1.2.7With an iron-sulfur protein as acceptor | 0.94 (0.69-1.29) | 0.714 | 67.4 | | 0.080 |  | | 0.98 (0.67-1.44) | | 0.933 | | 81.3 | | 0.021 |
| 3.1.7Diphosphoric-monoester hydrolases | 0.96 (0.75-1.22) | 0.715 | 73.3 | | 0.053 |  | | 0.95 (0.74-1.2) | | 0.646 | | 77.6 | | 0.035 |
| 2.1.2Hydroxymethyl-, formyl- and related transferases | 1.09 (0.69-1.73) | 0.715 | 17.4 | | 0.271 |  | | 0.95 (0.66-1.38) | | 0.806 | | 0.0 | | 0.382 |
| 3.3.1Thioether and trialkylsulfonium hydrolases | 0.93 (0.65-1.34) | 0.715 | 67.0 | | 0.082 |  | | 0.98 (0.7-1.36) | | 0.893 | | 65.7 | | 0.088 |
| 2.3.3Acyl groups converted into alkyl groups on transfer | 1.09 (0.68-1.73) | 0.724 | 0.0 | | 0.500 |  | | 1.03 (0.67-1.56) | | 0.905 | | 0.0 | | 0.378 |
| 3.8.1In carbon-halide compounds | 0.98 (0.85-1.12) | 0.724 | 0.0 | | 0.933 |  | | 1.01 (0.89-1.15) | | 0.894 | | 0.0 | | 0.794 |
| 3.1.6Sulfuric-ester hydrolases | 0.98 (0.87-1.1) | 0.728 | 14.6 | | 0.279 |  | | 1 (0.91-1.1) | | 0.991 | | 0.0 | | 0.373 |
| 1.17.1With NAD+ or NADP+ as acceptor | 1.06 (0.77-1.45) | 0.730 | 0.0 | | 0.388 |  | | 1.02 (0.76-1.38) | | 0.879 | | 13.4 | | 0.283 |
| 1.7.1With NAD+ or NADP+ as acceptor | 0.97 (0.83-1.14) | 0.732 | 0.0 | | 0.327 |  | | 0.98 (0.85-1.13) | | 0.788 | | 0.0 | | 0.370 |
| 1.16.1With NAD+ or NADP+ as acceptor | 1.02 (0.93-1.11) | 0.732 | 0.0 | | 0.857 |  | | 1.03 (0.95-1.12) | | 0.485 | | 0.0 | | 0.634 |
| 1.12.5With a quinone or similar compound as acceptor | 1.02 (0.9-1.16) | 0.739 | 0.0 | | 0.978 |  | | 0.97 (0.86-1.09) | | 0.630 | | 0.0 | | 0.865 |
| 2.-.- | 0.95 (0.72-1.26) | 0.741 | 0.0 | | 0.630 |  | | 0.93 (0.71-1.2) | | 0.555 | | 0.0 | | 0.694 |
| 1.14.12With NADH or NADPH as one donor, and incorporationoftwo atoms of oxygen into the other donor | 1.02 (0.89-1.17) | 0.742 | 14.0 | | 0.281 |  | | 1.05 (0.94-1.17) | | 0.418 | | 0.0 | | 0.444 |
| 3.1.21Endodeoxyribonucleases producing 5-phosphomonoesters | 0.91 (0.51-1.63) | 0.748 | 82.0 | | 0.019 |  | | 0.85 (0.48-1.51) | | 0.578 | | 84.4 | | 0.011 |
| 3.1.5Triphosphoric-monoester hydrolases | 1.03 (0.87-1.22) | 0.752 | 0.0 | | 0.353 |  | | 1.02 (0.87-1.19) | | 0.850 | | 0.0 | | 0.648 |
| 3.7.1In ketonic substances | 0.95 (0.69-1.3) | 0.757 | 0.0 | | 0.652 |  | | 1 (0.75-1.32) | | 0.977 | | 0.0 | | 0.987 |
| 1.11.1Peroxidases | 1.07 (0.68-1.68) | 0.760 | 0.0 | | 0.984 |  | | 0.97 (0.64-1.47) | | 0.892 | | 0.0 | | 0.727 |
| 1.20.4With disulfide as acceptor | 0.95 (0.69-1.31) | 0.766 | 77.7 | | 0.034 |  | | 0.98 (0.78-1.23) | | 0.846 | | 67.4 | | 0.080 |
| 1.11.2Peroxygenases | 0.97 (0.79-1.19) | 0.767 | 40.9 | | 0.193 |  | | 0.93 (0.71-1.22) | | 0.617 | | 68.4 | | 0.075 |
| 3.4.22Cysteine endopeptidases | 1.05 (0.76-1.44) | 0.774 | 82.5 | | 0.017 |  | | 1.01 (0.81-1.26) | | 0.932 | | 69.4 | | 0.071 |
| 4.1.99Other carbon-carbon lyases | 0.94 (0.6-1.47) | 0.777 | 0.0 | | 0.613 |  | | 0.91 (0.61-1.35) | | 0.632 | | 0.0 | | 0.546 |
| 2.8.3CoA-transferases | 1.04 (0.77-1.41) | 0.779 | 70.9 | | 0.064 |  | | 1.05 (0.84-1.33) | | 0.664 | | 57.4 | | 0.125 |
| 3.4.23Aspartic endopeptidases | 1.06 (0.72-1.54) | 0.782 | 0.0 | | 0.976 |  | | 0.93 (0.66-1.31) | | 0.669 | | 0.0 | | 0.690 |
| 1.14.99Miscellaneous | 0.98 (0.85-1.13) | 0.782 | 0.0 | | 0.571 |  | | 1.03 (0.87-1.22) | | 0.733 | | 36.6 | | 0.209 |
| 5.3.1Interconverting aldoses and ketoses, and related compounds | 1.09 (0.58-2.03) | 0.791 | 35.7 | | 0.213 |  | | 0.96 (0.56-1.67) | | 0.891 | | 34.0 | | 0.218 |
| 3.5.4In cyclic amidines | 1.06 (0.68-1.66) | 0.795 | 0.0 | | 0.654 |  | | 0.98 (0.65-1.47) | | 0.918 | | 0.0 | | 0.550 |
| 1.8.5With a quinone or similar compound as acceptor | 1.01 (0.92-1.12) | 0.798 | 0.0 | | 0.985 |  | | 0.95 (0.87-1.04) | | 0.302 | | 0.0 | | 0.936 |
| 3.4.17Metallocarboxypeptidases | 1.02 (0.89-1.17) | 0.800 | 0.0 | | 0.437 |  | | 0.98 (0.87-1.12) | | 0.796 | | 0.0 | | 0.538 |
| 2.7.8Transferases for other substituted phosphate groups | 1.05 (0.71-1.55) | 0.811 | 0.0 | | 0.913 |  | | 0.93 (0.65-1.35) | | 0.717 | | 0.0 | | 0.866 |
| 6.4.1Ligases that form carbon-carbon bonds (only sub-subclass identified to date) | 0.96 (0.67-1.37) | 0.822 | 0.0 | | 0.413 |  | | 0.97 (0.71-1.33) | | 0.849 | | 0.0 | | 0.593 |
| 2.9.1Selenotransferases | 1.02 (0.87-1.18) | 0.823 | 42.4 | | 0.188 |  | | 1.01 (0.88-1.17) | | 0.844 | | 44.9 | | 0.178 |
| 1.1.98With other, known, acceptors | 0.98 (0.84-1.15) | 0.831 | 35.5 | | 0.213 |  | | 1 (0.84-1.2) | | 0.987 | | 55.4 | | 0.134 |
| 2.7.11Protein-serine/threonine kinases | 0.94 (0.52-1.7) | 0.832 | 82.1 | | 0.018 |  | | 0.86 (0.53-1.42) | | 0.565 | | 76.7 | | 0.038 |
| 1.21.-Acting on X-H and Y-H to form an X-Y bond | 1.01 (0.9-1.13) | 0.841 | 0.0 | | 0.831 |  | | 1.01 (0.92-1.12) | | 0.801 | | 0.0 | | 0.599 |
| 2.7.9Phosphotransferases with paired acceptors | 1.03 (0.76-1.39) | 0.849 | 0.0 | | 0.319 |  | | 1 (0.76-1.31) | | 0.979 | | 0.0 | | 0.410 |
| 1.1.2With a cytochrome as acceptor | 0.98 (0.81-1.19) | 0.863 | 0.0 | | 0.781 |  | | 1.01 (0.85-1.2) | | 0.875 | | 0.0 | | 0.802 |
| 6.6.1Forming coordination complexes | 0.99 (0.84-1.16) | 0.879 | 0.0 | | 0.457 |  | | 1.07 (0.93-1.24) | | 0.336 | | 0.0 | | 0.876 |
| 3.6.3Acting on acid anhydrides to catalyse transmembranemovement of substances | 1.06 (0.51-2.21) | 0.883 | 62.8 | | 0.101 |  | | 0.94 (0.45-1.95) | | 0.866 | | 68.2 | | 0.076 |
| 3.1.26Endoribonucleases producing 5-phosphomonoesters | 1.05 (0.55-1.99) | 0.888 | 46.6 | | 0.171 |  | | 0.88 (0.53-1.48) | | 0.639 | | 33.6 | | 0.220 |
| 2.7.3Phosphotransferases with a nitrogenous group as acceptor | 0.98 (0.77-1.25) | 0.893 | 10.0 | | 0.292 |  | | 0.94 (0.76-1.16) | | 0.566 | | 0.0 | | 0.403 |
| 2.4.99Transferring other glycosyl groups | 0.95 (0.41-2.2) | 0.902 | 76.5 | | 0.039 |  | | 0.99 (0.54-1.84) | | 0.979 | | 63.8 | | 0.097 |
| 3.11.1Acting on carbon-phosphorus bonds (only sub-subclass identified to date) | 0.99 (0.88-1.12) | 0.903 | 0.0 | | 0.578 |  | | 1.03 (0.92-1.15) | | 0.652 | | 0.0 | | 0.734 |
| 2.4.2Pentosyltransferases | 1.04 (0.55-1.94) | 0.906 | 55.8 | | 0.132 |  | | 0.93 (0.56-1.54) | | 0.772 | | 46.7 | | 0.171 |
| 1.1.3With oxygen as acceptor | 0.99 (0.8-1.22) | 0.918 | 0.0 | | 0.392 |  | | 1 (0.77-1.31) | | 0.974 | | 49.3 | | 0.160 |
| 1.3.99With other acceptors | 1.03 (0.63-1.67) | 0.919 | 0.0 | | 0.378 |  | | 0.89 (0.5-1.59) | | 0.692 | | 37.2 | | 0.207 |
| 3.2.2Hydrolysing N-glycosyl compounds | 1.02 (0.68-1.53) | 0.919 | 15.2 | | 0.278 |  | | 0.96 (0.69-1.34) | | 0.804 | | 1.1 | | 0.315 |
| 3.13.1Acting on carbon-sulfur bonds (only sub-subclass identified to date) | 1 (0.92-1.09) | 0.931 | 0.0 | | 0.458 |  | | 1 (0.93-1.09) | | 0.949 | | 0.0 | | 0.668 |
| 1.10.3With oxygen as acceptor | 0.99 (0.82-1.2) | 0.935 | 22.5 | | 0.256 |  | | 0.98 (0.84-1.14) | | 0.804 | | 0.0 | | 0.334 |
| 3.1.31Endoribonucleases that are active with either ribo-ordeoxyribonucleic acids and produce 3-phosphomonoesters | 0.99 (0.86-1.15) | 0.936 | 48.3 | | 0.164 |  | | 1.02 (0.92-1.12) | | 0.749 | | 13.8 | | 0.282 |
| 6.3.4Other carbon-nitrogen ligases | 1.02 (0.65-1.6) | 0.939 | 19.6 | | 0.265 |  | | 0.92 (0.61-1.38) | | 0.685 | | 18.2 | | 0.269 |
| 3.6.4Acting on acid anhydrides to facilitate cellular andsubcellular movement | 1.02 (0.61-1.71) | 0.943 | 37.4 | | 0.206 |  | | 0.92 (0.62-1.39) | | 0.705 | | 21.4 | | 0.259 |
| 5.1.3Acting on carbohydrates and derivatives | 0.97 (0.44-2.15) | 0.946 | 51.6 | | 0.151 |  | | 0.85 (0.37-1.99) | | 0.711 | | 65.8 | | 0.087 |
| 2.7.2Phosphotransferases with a carboxy group as acceptor | 0.99 (0.67-1.46) | 0.947 | 0.0 | | 0.328 |  | | 0.86 (0.6-1.23) | | 0.400 | | 0.0 | | 0.452 |
| 4.1.1Carboxy-lyases | 1.02 (0.63-1.63) | 0.951 | 1.4 | | 0.314 |  | | 0.93 (0.55-1.56) | | 0.778 | | 29.5 | | 0.234 |
| 3.6.1In phosphorus-containing anhydrides | 1.02 (0.57-1.82) | 0.957 | 48.2 | | 0.165 |  | | 0.92 (0.48-1.75) | | 0.800 | | 64.2 | | 0.095 |
| 1.15.1Acting on superoxide as acceptor (only sub-subclassidentified to date) | 0.99 (0.65-1.51) | 0.959 | 0.0 | | 0.833 |  | | 0.9 (0.61-1.31) | | 0.570 | | 0.0 | | 0.626 |
| 1.17.7With an iron-sulfur protein as acceptor | 0.99 (0.69-1.43) | 0.959 | 0.0 | | 0.433 |  | | 0.9 (0.65-1.25) | | 0.537 | | 0.0 | | 0.445 |
| 2.1.1Methyltransferases | 0.99 (0.62-1.59) | 0.982 | 1.2 | | 0.314 |  | | 0.89 (0.54-1.45) | | 0.630 | | 24.4 | | 0.250 |
| 3.1.3Phosphoric-monoester hydrolases | 1 (0.7-1.45) | 0.983 | 1.5 | | 0.314 |  | | 0.93 (0.49-1.75) | | 0.821 | | 67.2 | | 0.081 |
| 1.14.13With NADH or NADPH as one donor, and incorporationofone atom of oxygen into the other donor | 1.01 (0.44-2.31) | 0.987 | 81.7 | | 0.019 |  | | 1.02 (0.54-1.93) | | 0.958 | | 72.4 | | 0.057 |
| 3.4.14Dipeptidyl-peptidases and tripeptidyl-peptidases | 1 (0.88-1.14) | 0.992 | 8.3 | | 0.296 |  | | 0.98 (0.88-1.09) | | 0.693 | | 0.0 | | 0.341 |
| 2.4.1Hexosyltransferases | 1 (0.54-1.85) | 0.997 | 59.3 | | 0.117 |  | | 0.93 (0.53-1.63) | | 0.787 | | 61.3 | | 0.108 |
| 5.2.1cis-trans Isomerases (only sub-subclass identified to date) | 1 (0.7-1.42) | 0.998 | 0.0 | | 0.407 |  | | 0.9 (0.65-1.25) | | 0.530 | | 0.0 | | 0.646 |
| 1.5.1With NAD+ or NADP+ as acceptor | 1 (0.63-1.6) | 0.998 | 13.2 | | 0.283 |  | | 0.87 (0.49-1.52) | | 0.615 | | 46.3 | | 0.172 |

^a^ Multivariate model adjusting for age, gender, tumor stage, and cigarette smoking in Cox proportional hazards models.

**Supplemental Table 4. Spearman correlation of the clr-corrected abundance of survival-associated bacterial species and the differentially immunologically expressed genes with *P* < 0.05.**

| Taxa | Gene | TCGA | | ICGC | | Gene Category |
| --- | --- | --- | --- | --- | --- | --- |
|  |  | *r* | *P* | *r* | *P* |  |
| *Elizabethkingia anophelis* | *AKT3* | 0.19 | 0.024 | -0.29 | 0.028 | BCR signaling Pathway, TCR signaling Pathway |
| *Acinetobacter johnsonii* | *ANGPTL1* | -0.18 | 0.037 | -0.30 | 0.019 | Cytokine Receptors |
| *Acinetobacter johnsonii* | *APOBEC3F* | -0.24 | 5.07E-03 | 0.31 | 0.017 | Antimicrobials |
| *Acinetobacter johnsonii* | *AVPR2* | -0.25 | 3.20E-03 | -0.27 | 0.042 | Cytokine Receptors |
| *Pseudomonas luteola* | *AVPR2* | -0.24 | 5.03E-03 | 0.30 | 0.020 | Cytokine Receptors |
| *Shigella flexneri* | *AVPR2* | -0.18 | 0.035 | -0.30 | 0.023 | Cytokine Receptors |
| *Acinetobacter johnsonii* | *BDNF* | -0.18 | 0.029 | -0.27 | 0.042 | Cytokines |
| *Elizabethkingia anophelis* | *BID* | -0.19 | 0.028 | 0.28 | 0.029 | Natural Killer Cell Cytotoxicity |
| *Escherichia coli* | *BMP3* | 0.20 | 0.018 | -0.27 | 0.041 | Cytokines, TGFb Family Member |
| *Pseudomonas luteola* | *BMPR1A* | 0.19 | 0.028 | 0.26 | 0.048 | Cytokine Receptors, TGFb Family Member Receptor |
| *Hymenobacter sp. IS2118* | *CANX* | 0.26 | 2.21E-03 | -0.26 | 0.048 | Antigen Processing and Presentation |
| *Chelatococcus sambhunathii* | *CCR4* | -0.18 | 0.035 | -0.26 | 0.047 | Cytokine Receptors, Chemokine Receptors, Antimicrobials |
| *Arthrobacter sp. L77* | *CD244* | -0.24 | 4.45E-03 | 0.28 | 0.035 | Natural Killer Cell Cytotoxicity |
| *Arthrobacter sp. L77* | *CD74* | -0.17 | 0.042 | -0.33 | 0.011 | Antigen Processing and Presentation |
| *Hymenobacter sp. IS2118* | *CETP* | -0.17 | 0.040 | -0.33 | 0.011 | Antimicrobials |
| *Pseudomonas luteola* | *CETP* | -0.20 | 0.020 | -0.33 | 0.012 | Antimicrobials |
| *Hymenobacter sp. IS2118* | *CGB7* | -0.20 | 0.016 | 0.30 | 0.022 | Cytokines |
| *Hymenobacter sp. IS2118* | *CORT* | -0.18 | 0.034 | 0.28 | 0.030 | Cytokines |
| *Chelatococcus sambhunathii* | *CSRP1* | -0.18 | 0.037 | 0.31 | 0.017 | Antimicrobials |
| *Pseudomonas luteola* | *DCK* | 0.30 | 2.98E-04 | -0.34 | 9.80E-03 | Antimicrobials |
| *Pseudomonas luteola* | *EGFR* | 0.33 | 8.80E-05 | -0.37 | 3.93E-03 | Cytokine Receptors |
| *Pseudomonas luteola* | *F2RL1* | 0.21 | 0.014 | -0.28 | 0.033 | Antimicrobials |
| *Elizabethkingia anophelis* | *FGF8* | -0.22 | 9.05E-03 | 0.28 | 0.034 | Cytokines |
| *Pseudomonas luteola* | *FGF8* | -0.17 | 0.041 | 0.28 | 0.032 | Cytokines |
| *Streptococcus infantis* | *FGFR3* | -0.21 | 0.013 | 0.28 | 0.031 | Cytokine Receptors |
| *Pseudomonas luteola* | *FLT3LG* | -0.28 | 6.92E-04 | 0.26 | 0.044 | Cytokines |
| *Acinetobacter johnsonii* | *FLT4* | -0.22 | 9.46E-03 | 0.34 | 8.59E-03 | Cytokine Receptors |
| *Arthrobacter sp. L77* | *FYN* | -0.17 | 0.044 | -0.33 | 0.012 | TCR signaling Pathway, Natural Killer Cell Cytotoxicity |
| *Gardnerella vaginalis* | *GDF6* | 0.17 | 0.041 | -0.26 | 0.043 | TGFb Family Member, Cytokines |
| *Alcaligenes faecalis* | *GFAP* | -0.19 | 0.023 | 0.26 | 0.048 | Antimicrobials |
| *Streptococcus infantis* | *GHRHR* | -0.22 | 9.62E-03 | 0.46 | 3.14E-04 | Cytokine Receptors |
| *Alcaligenes faecalis* | *GMFB* | 0.21 | 0.013 | -0.28 | 0.033 | Cytokines |
| *Pseudomonas luteola* | *GNAI1* | 0.25 | 2.98E-03 | -0.29 | 0.026 | Antimicrobials |
| *Elizabethkingia anophelis* | *HNF4A* | 0.17 | 0.049 | 0.39 | 2.17E-03 | Cytokine Receptors |
| *Elizabethkingia anophelis* | *HSP90AA1* | 0.27 | 1.09E-03 | -0.26 | 0.045 | Antigen Processing and Presentation |
| *Elizabethkingia anophelis* | *HSPA8* | 0.17 | 0.042 | -0.27 | 0.040 | Antigen Processing and Presentation |
| *Hymenobacter sp. IS2118* | *IFNA5* | -0.20 | 0.017 | 0.27 | 0.040 | Natural Killer Cell Cytotoxicity, Interferons, Cytokines, Antigen Processing and Presentation |
| *Pseudomonas luteola* | *IL11RA* | -0.18 | 0.030 | 0.39 | 2.55E-03 | Interleukins Receptor, Cytokine Receptors, Interleukins Receptor |
| *Hymenobacter sp. IS2118* | *IL17RC* | -0.27 | 1.23E-03 | -0.27 | 0.036 | Interleukins Receptor |
| *Mycobacterium sp. Root265* | *IL2* | -0.17 | 0.048 | -0.31 | 0.016 | TCR signaling Pathway, Interleukins, Cytokines, Antimicrobials |
| *Gardnerella vaginalis* | *IL2RA* | 0.18 | 0.032 | 0.36 | 4.71E-03 | Interleukins Receptor, Cytokine Receptors |
| *Pseudomonas luteola* | *IL7* | 0.21 | 0.014 | 0.30 | 0.023 | Interleukins, Cytokines |
| *Pseudomonas luteola* | *IRF3* | -0.17 | 0.049 | 0.31 | 0.016 | Antimicrobials |
| *Hymenobacter sp. IS2118* | *ISG20L2* | 0.18 | 0.034 | -0.29 | 0.026 | Antimicrobials |
| *Candidatus Burkholderia crenata* | *JAK2* | -0.18 | 0.038 | 0.26 | 0.043 | Antimicrobials |
| *Pseudomonas luteola* | *KIR3DL3* | 0.17 | 0.045 | 0.29 | 0.028 | Antigen Processing and Presentation |
| *Pseudomonas luteola* | *KLKB1* | -0.17 | 0.050 | 0.28 | 0.033 | Antimicrobials |
| *Pseudomonas luteola* | *KRAS* | 0.31 | 2.13E-04 | 0.26 | 0.045 | TCR signaling Pathway, BCR signaling Pathway |
| *Pseudomonas luteola* | *LMBR1* | 0.24 | 4.46E-03 | -0.29 | 0.026 | Antimicrobials |
| *Elizabethkingia anophelis* | *MAPK8* | 0.26 | 2.09E-03 | -0.28 | 0.035 | Antimicrobials |
| *Gardnerella vaginalis* | *MR1* | 0.21 | 0.011 | 0.27 | 0.038 | Antigen Processing and Presentation |
| *Streptococcus infantis* | *NFKBIB* | -0.24 | 4.87E-03 | -0.31 | 0.018 | TCR signaling Pathway, BCR signaling Pathway |
| *Elizabethkingia anophelis* | *NRP1* | 0.20 | 0.019 | -0.28 | 0.033 | Cytokine Receptors |
| *Pseudomonas luteola* | *NRP1* | 0.19 | 0.025 | -0.33 | 0.012 | Cytokine Receptors |
| *Gardnerella vaginalis* | *NUDT6* | -0.19 | 0.021 | 0.35 | 7.20E-03 | Cytokines |
| *Streptococcus infantis* | *ORM1* | -0.21 | 0.011 | -0.39 | 2.33E-03 | Antimicrobials |
| *Gardnerella vaginalis* | *OXT* | -0.19 | 0.024 | 0.27 | 0.042 | Cytokines |
| *Gardnerella vaginalis* | *PAK2* | 0.23 | 6.20E-03 | -0.26 | 0.050 | TCR signaling Pathway |
| *Hymenobacter sp. IS2118* | *PIK3CA* | 0.21 | 0.015 | -0.31 | 0.018 | TCR signaling Pathway, BCR signaling Pathway, Natural Killer Cell Cytotoxicity |
| *Pseudomonas luteola* | *PIK3CA* | 0.30 | 3.89E-04 | -0.31 | 0.016 | TCR signaling Pathway, BCR signaling Pathway, Natural Killer Cell Cytotoxicity |
| *Gardnerella vaginalis* | *PLXNC1* | 0.21 | 0.012 | 0.30 | 0.023 | Cytokine Receptors, Chemokine Receptors |
| *Gardnerella vaginalis* | *POMC* | -0.20 | 0.019 | -0.29 | 0.027 | Cytokines |
| *Chelatococcus sambhunathii* | *PPARA* | -0.17 | 0.045 | 0.29 | 0.029 | Cytokine Receptors |
| *Pseudomonas luteola* | *PPARA* | 0.36 | 1.25E-05 | 0.32 | 0.012 | Cytokine Receptors |
| *Shigella flexneri* | *PSMC4* | -0.18 | 0.036 | 0.38 | 2.98E-03 | Antigen Processing and Presentation |
| *Pseudomonas luteola* | *PSMC6* | 0.20 | 0.017 | -0.29 | 0.028 | Antigen Processing and Presentation |
| *Shigella flexneri* | *PSMD6* | -0.22 | 8.11E-03 | 0.38 | 2.76E-03 | Antigen Processing and Presentation |
| *Acinetobacter johnsonii* | *PTGDR* | -0.25 | 2.46E-03 | 0.28 | 0.031 | Cytokine Receptors, Antimicrobials |
| *Elizabethkingia anophelis* | *PYY* | -0.28 | 7.07E-04 | 0.29 | 0.026 | Cytokines |
| *Escherichia coli* | *RELA* | 0.23 | 7.35E-03 | 0.36 | 5.37E-03 | TCR signaling Pathway, BCR signaling Pathway, Antimicrobials |
| *Acinetobacter johnsonii* | *SEMA3B* | -0.21 | 0.014 | -0.30 | 0.020 | Cytokines, Chemokines |
| *Pseudomonas luteola* | *SEMA6C* | -0.20 | 0.017 | 0.29 | 0.024 | Cytokines, Chemokines |
| *Candidatus Burkholderia crenata* | *SEMA6D* | -0.18 | 0.038 | -0.32 | 0.015 | Cytokines, Chemokines |
| *Shigella flexneri* | *SPAG11B* | 0.18 | 0.034 | 0.26 | 0.047 | Antimicrobials |
| *Chelatococcus sambhunathii* | *STAT3* | -0.23 | 6.56E-03 | -0.26 | 0.046 | Antimicrobials |
| *Alcaligenes faecalis* | *SYK* | 0.22 | 8.59E-03 | 0.37 | 4.44E-03 | TCR signaling Pathway, Natural Killer Cell Cytotoxicity |
| *Elizabethkingia anophelis* | *THBS1* | 0.20 | 0.016 | -0.35 | 6.46E-03 | Antigen Processing and Presentation |
| *Pseudomonas luteola* | *THBS1* | 0.18 | 0.032 | -0.42 | 9.93E-04 | Antigen Processing and Presentation |
| *Shigella flexneri* | *THRA* | 0.20 | 0.018 | -0.26 | 0.045 | Cytokine Receptors |
| *Alcaligenes faecalis* | *THRB* | 0.25 | 3.23E-03 | 0.31 | 0.019 | Cytokine Receptors |
| *Elizabethkingia anophelis* | *THRB* | 0.24 | 4.44E-03 | 0.26 | 0.043 | Cytokine Receptors |
| *Hymenobacter sp. IS2118* | *THRB* | 0.23 | 6.14E-03 | 0.33 | 0.011 | Cytokine Receptors |
| *Pseudomonas luteola* | *THRB* | 0.25 | 2.84E-03 | 0.28 | 0.033 | Cytokine Receptors |
| *Hymenobacter sp. IS2118* | *TNFRSF14* | -0.21 | 0.014 | 0.38 | 3.56E-03 | TNF Family Members Receptors, Cytokine Receptors |
| *Pseudomonas luteola* | *TNFRSF21* | 0.22 | 0.010 | 0.29 | 0.025 | TNF Family Members Receptors, Cytokine Receptors |
| *Acinetobacter johnsonii* | *TNFRSF25* | -0.21 | 0.011 | -0.32 | 0.014 | TNF Family Members Receptors, Cytokine Receptors |
| *Escherichia coli* | *TNFSF15* | 0.22 | 0.010 | -0.30 | 0.023 | TNF Family Members, Cytokines |
| *Alcaligenes faecalis* | *TNFSF8* | 0.17 | 0.045 | -0.31 | 0.017 | TNF Family Members, Cytokines |
| *Chelatococcus sambhunathii* | *TNFSF8* | -0.17 | 0.048 | -0.36 | 5.51E-03 | TNF Family Members, Cytokines |
| *Pseudomonas luteola* | *TRH* | -0.19 | 0.025 | 0.29 | 0.029 | Cytokines |
| *Alcaligenes faecalis* | *TSHB* | 0.17 | 0.043 | 0.39 | 2.24E-03 | Cytokines |
| *Pseudomonas luteola* | *UBXN1* | -0.19 | 0.026 | 0.28 | 0.031 | Antigen Processing and Presentation |
| *Acinetobacter lwoffii* | *UCN* | -0.27 | 1.56E-03 | -0.28 | 0.034 | Cytokines |
| *Pseudomonas luteola* | *WFIKKN1* | -0.23 | 6.68E-03 | -0.33 | 0.010 | Antimicrobials |
| *Streptococcus infantis* | *WNT5A* | 0.18 | 0.031 | 0.36 | 5.76E-03 | Antimicrobials |
| *Acinetobacter johnsonii* | *ZAP70* | -0.24 | 4.09E-03 | -0.26 | 0.047 | TCR signaling Pathway, Natural Killer Cell Cytotoxicity |
| *Pseudomonas luteola* | *ZAP70* | -0.18 | 0.033 | 0.28 | 0.029 | TCR signaling Pathway, Natural Killer Cell Cytotoxicity |

**Supplemental Figure 1. Microbial community composition, by Cigarette smoking.** The α-diversity (Chao1 index, Shannon index, and Simpson index) and β-diversity (Jensen-Shannon divergence) by cigarette smoking in Combined cohorts (A), TCGA cohort (B), and ICGC cohort (C). Statistical significance for α-diversity presented in each panel was calculated using the two-sided unpaired Wilcoxon rank test (two groups) and linear regression with adjustment of age, sex, tumor stage, or cohort. Statistical significance for β-diversity presented in each panel was determined using permutational multivariate analysis of variance (PERMANOVA) with unadjusted and adjustment of age, sex, tumor stage, or cohort.

**Supplemental Figure 2. Microbial community composition, by Tumor stage.** The α-diversity (Chao1 index, Shannon index and Simpson index) and β-diversity (Jensen-Shannon divergence) by tumor stage in Combined cohorts (A), TCGA cohort (B), and ICGC cohort (C). Statistical significance for α-diversity presented in each panel was calculated using the two-sided unpaired Wilcoxon rank test (two groups) and linear regression with adjustment of age, sex, cigarette smoking, or cohort. Statistical significance for β-diversity presented in each panel was determined using permutational multivariate analysis of variance (PERMANOVA) with unadjusted and adjustment of age, sex, cigarette smoking, or cohort.

**Supplemental Figure 3. Microbial community composition, by Tumor grade.** The α-diversity (Chao1 index, Shannon index and Simpson index) and β-diversity (Jensen-Shannon divergence) by tumor grade in Combined cohorts (A), TCGA cohort (B), and ICGC cohort (C). Statistical significance for α-diversity presented in each panel was calculated using the two-sided unpaired Wilcoxon rank test (two groups) and linear regression with adjustment of age, sex, tumor stage, cigarette smoking, or cohort. Statistical significance for β-diversity presented in each panel was determined using permutational multivariate analysis of variance (PERMANOVA) with unadjusted and adjustment of age, sex, tumor stage, cigarette smoking, or cohort.

**Supplemental Figure 4. Microbial community composition, by chemotherapy** **and radiation therapy.** The α-diversity (Chao1 index, Shannon index and Simpson index) and β-diversity (Jensen-Shannon divergence) by chemotherapy (A) and radiation therapy (B) in TCGA cohort. For α-diversity analysis, crude *P*-values presented in each panel were calculated using the two-sided unpaired Wilcoxon rank test (two groups), and adjusted *P*-values presented in each panel were calculated using linear regression with adjustment of age, sex, tumor stage, cigarette smoking, chemotherapy or radiation therapy. For β-diversity analysis, statistical significance presented in each panel was determined using permutational multivariate analysis of variance (PERMANOVA) with unadjusted and adjustment of age, sex, tumor stage, cigarette smoking, chemotherapy, or radiation therapy.

**Supplemental Figure 5. Microbial community composition, by Mortality.** The α-diversity (Chao1 index, Shannon index, and Simpson index) and β-diversity (Jensen-Shannon divergence) by Mortality status in Combined cohorts (A), TCGA cohort (B), and ICGC cohort (C). Statistical significance for α-diversity presented in each panel was calculated using the two-sided unpaired Wilcoxon rank test (two groups) and linear regression with adjustment of age, sex, tumor stage, cigarette smoking, or cohort. Statistical significance for β-diversity presented in each panel was determined using permutational multivariate analysis of variance (PERMANOVA) with unadjusted and adjustment of age, sex, tumor stage, cigarette smoking, or cohort.

**Supplemental Figure 6. Microbial community composition, by Relapse.** The α-diversity (Chao1 index, Shannon index and Simpson index) and β-diversity (Jensen-Shannon divergence) by Relapse status in Combined cohorts (A), TCGA cohort (B), and ICGC cohort (C). Statistical significance for α-diversity presented in each panel was calculated using the two-sided unpaired Wilcoxon rank test (two groups) and linear regression with adjustment of age, sex, tumor stage, cigarette smoking, or cohort. Statistical significance for β-diversity presented in each panel was determined using permutational multivariate analysis of variance (PERMANOVA) with unadjusted and adjustment of age, sex, tumor stage, cigarette smoking, or cohort.

**Supplemental Figure 7. Comparison of tumor microbial species with survival in TCGA cohort with adjustment of standard and additional covariates.** Cox proportional hazards model comparison for identified 11 OS-associated Species (A) and 9 RFS-associated species (B) with early-stage PDAC patients in TCGA. Standard covariates include sex, age, cigarette smoking, and tumor stage; additional covariates include sex, age, cigarette smoking, tumor stage, chemotherapy, and radiation therapy.

**Supplemental Figure 8. Microbial Risk Score (MRS) with overall survival and relapse-free survival in early-stage PDAC by TCGA and ICGC.** Kaplan-Meier estimates for survival probability of MRS_Mortality_ and overall survival in TCGA (A) and ICGC (B) cohorts. Blue represents the high-risk patients, and green represents the low-risk (based on median) summary-weighted MRS index for OS-associated species. The dotted line represents the median survival time of each group. Kaplan-Meier curve of MRS_Relapse_ and relapse-free survival in TCGA (C) and ICGC (D) cohorts. Pink represents the high-risk patients, and grey-blue represents the low-risk (based on median) summary-weighted MRS index for relapse-associated species. The dotted line represents the median survival time of each group.

**Supplemental Figure 9. Associations between selected tumor microbial species and MRSs with early-stage PDAC OS and RFS, by cigarette smoking.** Tumor tissue microbiome among patients with PDAC OS (A) and RFS (B) stratified by cigarette smoking in the meta-analysis of TCGA and ICGC cohorts.

**Supplemental Figure 10. Associations between selected tumor microbial species and MRSs with early-stage PDAC OS and RFS, by tumor stage and grade.** Tumor tissue microbiome among patients with PDAC OS and RFS stratified by tumor stage (A-B) and grade (C-D) in the meta-analysis of TCGA and ICGC cohorts.

**Supplemental Figure 11. Associations between selected tumor microbial species and MRSs with early-stage PDAC OS and RFS, by therapy strategies in TCGA.** Tumor tissue microbiome among early-stage resected patients with PDAC OS and RFS (A-B) stratified by chemotherapy, first-line gemcitabine therapy (C-D), and radiation therapy (E-F).

**Supplemental Figure 12. Contribution of bacterial species and functional modules and pathways in PDAC early-stage patients.** (A) Association of microbial modules with PDAC OS and RFS with *P*-meta<0.05. (B) Spearman’s correlations are shown for bacterial species vs. modules. (C) Association of microbial pathways with PDAC OS and RFS with *P*<0.05. (D) Spearman’s correlations are shown for bacterial species vs. pathways. Only OS-associated and RFS-associated taxa are shown, and only microbial modules and pathways selected in the meta-analysis (*P*-meta<0.05) of TCGA and ICGC cohorts are shown. Taxa and modules with relative abundance were used for correlation analysis. *p<0.05. The color of the bar indicates the meta Hazard Ratio (HR) of Species in Cox PH model, ranging from HR <1, in green, to HR>1, in yellow.

**Supplemental Figure 13. The workflow for identifying prognostic microbial species across TCGA and ICGC cohorts.**

**
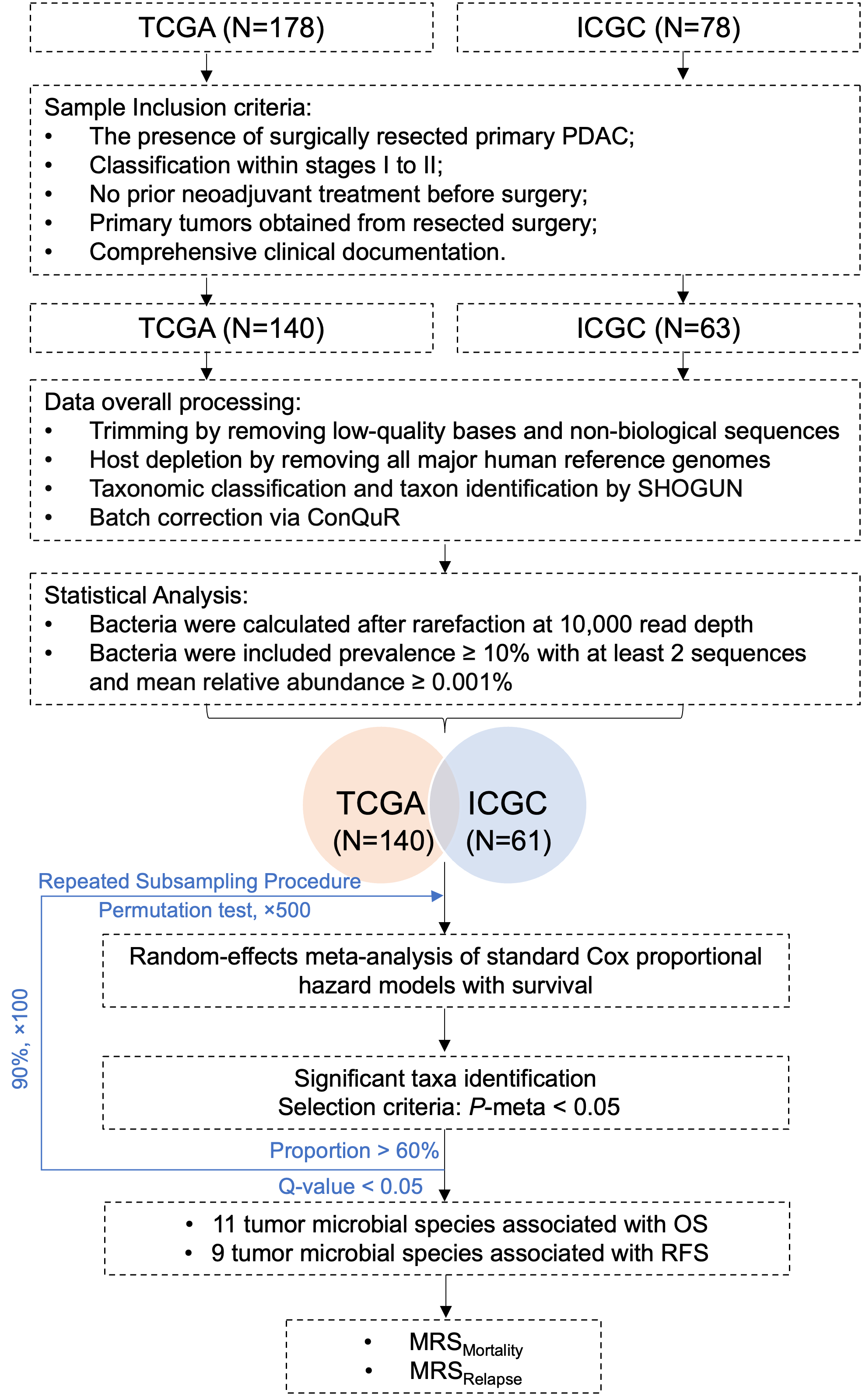
**

**Supplemental Figure 14. Bacterial sequence reads before and after host depletion.** After a rigorous “exhaustive” filtering process with human sequence removal, two samples in ICGC showed no mapping of reads to reference genomes.

**Supplemental Figure 15. Bacterial rarefaction analysis curves between PDAC early-stage patients.** The rarefaction analysis curves comparing the Shannon index for RNA-seq data by TCGA and ICGC cohorts. The 10,000 sequence threshold was chosen according to the rarefaction curves.
